# Manipulation of the carbon storage regulator system for metabolite remodeling and biofuel production in *Escherichia coli*

**DOI:** 10.1186/1475-2859-11-79

**Published:** 2012-06-13

**Authors:** Adrienne E McKee, Becky J Rutherford, Dylan C Chivian, Edward K Baidoo, Darmawi Juminaga, Dwight Kuo, Peter I Benke, Jeffrey A Dietrich, Suzanne M Ma, Adam P Arkin, Christopher J Petzold, Paul D Adams, Jay D Keasling, Swapnil R Chhabra

**Affiliations:** 1 Joint BioEnergy Institute, Emeryville, CA, USA; 2 Physical Biosciences Division, Lawrence Berkeley National Laboratory, Berkeley, CA, USA; 3 Department of Bioengineering, University of California, Berkeley, CA, USA; 4 Department of Chemical and Biomolecular Engineering, University of California, Berkeley, CA, USA

**Keywords:** Metabolic engineering, Global regulators, Heterologous pathway, Carbon storage, Biofuels, Metabolomics, Proteomics

## Abstract

**Background:**

Microbial engineering strategies that elicit global metabolic perturbations have the capacity to increase organism robustness for targeted metabolite production. In particular, perturbations to regulators of cellular systems that impact glycolysis and amino acid production while simultaneously decreasing fermentation by-products such as acetate and CO_2_ make ideal targets. Intriguingly, perturbation of the Carbon Storage Regulator (Csr) system has been previously implicated in large changes in central carbon metabolism in *E. coli*. Therefore, we hypothesized that perturbation of the Csr system through the CsrA-CsrB ribonucleoprotein complex might increase production of biofuels and their intermediates from heterologous pathways.

**Results:**

We engaged the CsrA-CsrB ribonucleoprotein complex of *E. coli* via overexpression of CsrB. CsrB is a 350-nucleotide non-coding RNA that antagonizes CsrA, an RNA-binding protein that regulates translation of specific mRNA targets. By using shotgun proteomics and targeted metabolomics we established that elevation of CsrB levels leads to alterations in metabolite and protein levels in glycolysis, the TCA cycle and amino acid levels. Consequently, we show that such changes can be suitably applied to improve the production of desired compounds through the native fatty acid and heterologous *n*-butanol and isoprenoid pathways by up to two-fold. We also observed concomitant decreases in undesirable fermentation by-products such as acetate and CO_2_.

**Conclusions:**

We have demonstrated that simple engineering of the RNA-based Csr global regulatory system constitutes a novel approach to obtaining pathway-independent improvements within engineered hosts. Additionally, since Csr is conserved across most prokaryotic species, this approach may also be amenable to a wide variety of production hosts.

## Background

Strategies for increasing organism robustness and product formation by manipulation of *non*-pathway components have gained traction in recent years [1]. Prominent examples include the reprogramming of transcriptional machinery achieved by varying the concentration or sequence of sigma factor proteins or the perturbation of regulators that alter cellular metabolism [2-5]. Arguably, the ideal perturbation(s) would lead to higher product formation by altering central carbon metabolism, result in higher amino acid production for the biogenesis of heterologous proteins, and reduce fermentation by-products such as CO_2_ and acetate that decrease product yield. In this study, we examined the carbon storage regulator (Csr) system of *E. coli* as one potential system that could be manipulated to bring about such changes.

The carbon storage regulator (Csr) system of *E. coli* has been shown to regulate over 700 genes and can exert control over global regulatory systems, such as the stringent response [6]. Csr influences a number of physiological processes including central carbon metabolism, biofilm development, motility, peptide uptake, and virulence gene expression [7-10]. Interestingly, manipulation of Csr has also been demonstrated to increase production of phenylalanine [11,12]. As such, Csr is a key cellular subsystem that might be exploited for increased production of compounds from both native and heterologous pathways.

**Figure 1:**
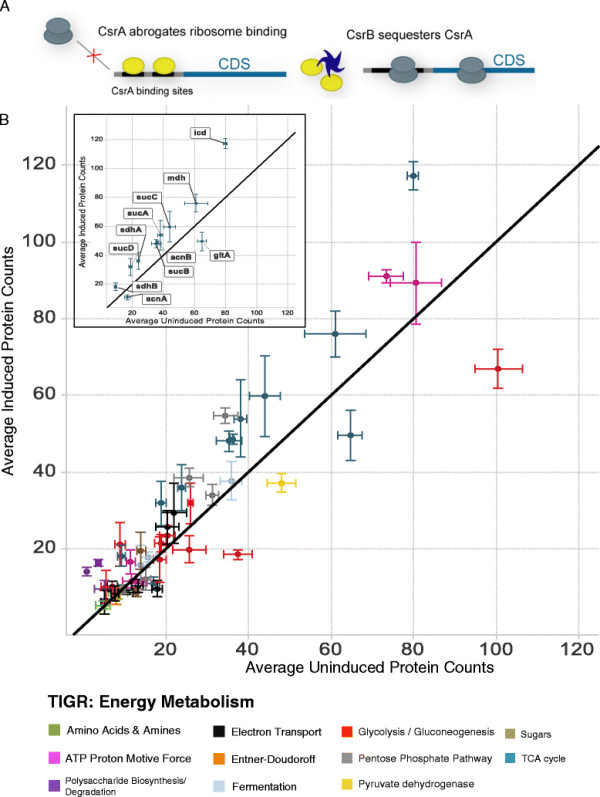
**The carbon storage regulator system in*E. coli.*A**) The RNA-binding protein CsrA (yellow ovals) associates with target transcripts and impedes their translation by interfering with ribosome binding. The non-coding RNA antagonist of CsrA, CsrB (blue star), sequesters CsrA, thereby removing the hindrance that CsrA places on translation of its targets. Not shown here are CsrD and CsrC, protein and RNA negative regulators of CsrA. **B**) Averaged spectral counts for proteins in the Energy Metabolism TIGR group from samples containing the pBbA5C-CsrB plasmid (induced) or pBbA5C plasmid (uninduced) with 500 uM IPTG harvested at 24 hours after induction. Proteins from the TCA cycle are highlighted in the figure inset. Significant increases were observed in both these groups.

Csr is controlled by the RNA-binding protein CsrA [13-15], which binds to the 5’ untranslated region of its target mRNAs, often in the region spanning the Shine-Dalgarno (SD) site [16] (Figure 1A). Interaction of CsrA with mRNA interferes with ribosome binding, negatively impacting translation of target transcripts. While CsrA directly regulates the activity of glycolysis pathway components both positively (glucose-6-phosphate isomerase (*pgi*), triose-phosphate isomerase (*tpiA*), and enolase (*eno*)) and negatively (fructose-1,6-bisphosphatase (*fbp*) and phosphoenolpyruvate synthetase (*pps*)) [17], CsrA is essential to *E. coli* during growth on glycolytic carbon sources due to its ability to limit glycogen accumulation [18]. Regulation of CsrA can occur by physical interaction with CsrB, a non-coding small RNA [19-21]. CsrB prevents CsrA from binding target transcripts thus alleviating CsrA-induced translation inhibition (Figure 1A). In fact, it has been proposed that the intracellular level of CsrB is the key determinant of CsrA activity in the cell [22].

As Csr regulates a wide variety of cellular processes including parts of central carbon metabolism, we hypothesized that manipulation of CsrB expression might alter the activity of CsrA resulting in changes to *E. coli* metabolism favorable to product formation. To investigate this possibility, we evaluated the effects of CsrB elevation on the proteome, on metabolites of central carbon metabolism, and on the production of several advanced biofuels or their precursors in *E. coli*. We demonstrate that the Csr system can serve as an effective regulatory handle for improving production via a number of engineered pathways, reducing the need for extensive host-chromosome modifications.

## Results

### CsrB-mediated proteome changes

We reasoned that since CsrB regulatory control occurs at the post-transcriptional level, quantitative proteomic measurements would offer the most direct assessment of relevant cellular changes. Therefore, we employed a shotgun proteomics approach to compare protein levels in *E. coli* with elevated CsrB levels to those from an uninduced control. We chose to profile cells during stationary phase when Csr is most active in remodeling cellular metabolism [23]. This also corresponds to when engineered bacteria are typically harvested for biofuel production.

*E. coli* (see Additional file 1: Table S1) with or without elevated CsrB levels were grown in Neidhardt’s MOPS medium with IPTG for 24 hours, and harvested for proteomic analysis. At a 95% confidence interval, 894 proteins (~20% genomic coverage) were identified with at least two peptides (Figure 1B). Candidates with an average of five or more normalized spectral counts in the induced samples and at least a 2-fold change upon induction are listed in Additional file 2: Table S2 (see Additional file 3: Table S5 for the complete list of proteins identified in this study). We confirmed increased protein levels of known CsrA mRNA targets *glgB, glgC*[23], and *ompA*[24] (Additional file 2: Table S2), which upon CsrB elevation were observed to rise 16.7-, 4.5-, and 2.8-fold respectively.

Using TIGR Gene Indices and gene ontology (GO) categories that provide genome-wide functional annotations for related genes [25], we observed that CsrB elevation significantly affected the protein abundance of several functional categories (*P* < 0.05, multiple-test corrected hypergeometric enrichment test). Many of the proteins in TIGR categories such as ‘Energy metabolism’, ‘glycogen biosynthetic process’, and ‘amino acid biosynthesis’ displayed CsrB-dependent expression changes that were greater than twice the level observed for the control strain. In particular, we noted several proteins involved in central carbon metabolism – Pck, TpiA, GapA, and Eno from glycolysis/gluconeogenesis, the SdhAB and SucABCD operons associated with the TCA cycle as well as TktA, TalB, and AtpG involved in the pentose phosphate pathway showed significant changes in protein expression. Consistent with these results, we also observed that genes under the regulatory control of CysB [26] were significantly overrepresented (*P* < 0.05) among proteins with altered expression. CysB has been previously implicated in modulating carbon source utilization during limited nutrient availability [27].

Additionally, proteins with altered expression also included those associated with stress response (PspA, GrpE, AhpC), transport (CysA, TolC, SecB), and regulatory functions (DksA, RraA, AllR). In particular, the change in protein expression of DksA corroborates a previously proposed regulatory interaction between Csr and stringent response [6] (Additional file 4: Figure S1A). Currently, the stringent response transcription factor DksA and the nucleotide secondary messenger ppGpp are thought to activate Csr by increasing transcription of *csrB/C* through BarA/UvrY*,* thereby relieving CsrA-mediated repression during the stringent response [6]. However, it was also reported that CsrA has only a modest effect on DksA gene expression. In contrast, we observed a greater than 2.5-fold increase in the expression of DksA upon CsrB elevation suggesting a more substantial role for CsrA regulation of DksA expression.

### RNA-binding motifs of CsrA

Given the technical limitations of shotgun proteomics (limited to 20% genomic coverage in this study) we wanted to assess the broader impact of perturbing CsrA-mediated regulation in *E. coli*. Therefore, to identify putative CsrA-regulated genes, we investigated the distribution of three variants of an 8-base pair (bp), position-independent, degenerate CsrA binding motif within a 22-bp window upstream of the translational start site of each *E. coli* gene (see Supplemental Methods for details on motif generation). The first motif was derived directly from experimentally determined binding sites (Motif 0: [~G]ANGGAN[A/U]). We further refined Motif 0 to generate Motif 1: [~G]A[~C]GGA[~C][A/U] and Motif 8:[~G]A[~C]GGA[~C][~G]), which showed the highest combination of sensitivity and specificity (Additional file 4: Figure S1B,C) for proteins identified by our mass spectrometry experiments.

Our search discovered hundreds (Motif 0: 689 genes, Motif 1: 575 genes, Motif 8: 702 genes) of protein-coding genes that possess putative sites immediately upstream of their translational start (Additional file 5: Table S4, Additional file 3: Table S5). Examining the set of differentially expressed proteins from our screen revealed that they were significantly enriched for these motifs (*P* < 0.02, hypergeometric enrichment test). Among these genes, we identified previously reported targets with roles in glycogen synthesis, peptidoglycan formation, peptide import, and RNA metabolism [7,14]. Interestingly, we also identified CsrA-binding sites among genes responsible for chaperones involved in the general stress response of *E. coli*, iron homeostasis, and transcriptional and translational regulators. These observations suggest that Csr regulates additional functions than what has been described so far [6].

Next, we examined our proteomics results in conjunction with previously derived TF regulons [26]. While we found several regulons with genes that possessed CsrA binding sites, several regulons also had the cognate transcription factor itself under putative CsrA regulation. Examples of such systems include those involved in carbon utilization, stress response, and regulation of quorum sensing and biofilms. In particular, we noted that the FabR/FadR regulons were also putatively CsrA-regulated. These regulons directly impact fatty acid biosynthesis and the AtoC regulon, which includes AtoB, a key enzyme for acetoacetyl-CoA synthesis. As such, these regulons are critical for pathway engineering applications that rely on acetyl-CoA as a branch point metabolite.

### CsrB-mediated alterations in central carbon metabolism

From the shotgun proteomics measurements, we noted changes in levels of several proteins from glycolysis/gluconeogenesis, the TCA cycle, and the pentose phosphate pathway upon CsrB elevation. Additionally, our genome wide survey of CsrA binding motifs suggested a potential impact on the AtoC regulon, which affects acetyl-CoA levels. Consequently, we proceeded to examine the direct impact of perturbing CsrB levels on intracellular metabolites associated with central carbon metabolism. *E. coli* with or without elevated CsrB levels were grown in Neidhardt’s MOPS medium with IPTG (50 *μ*M) and harvested after 48 hrs. All measurements were performed with liquid chromatography and mass spectrometry analysis except for amino acid metabolites that were determined by capillary electrophoresis and mass spectrometry.

**Figure 2:**
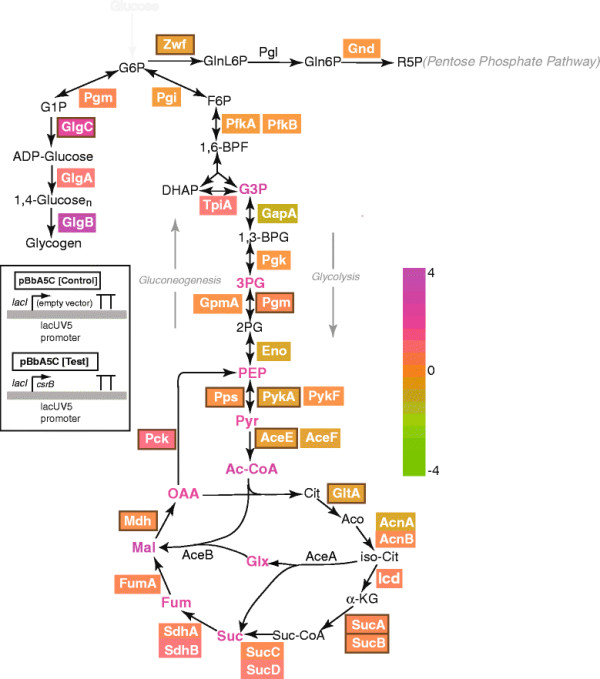
**Extensive metabolic remodeling is achieved through CsrB manipulation.** Average fold ratio of intracellular metabolites and proteins from central metabolism in BLR-DAJ cells bearing pBbA5C-CsrB relative to the empty plasmid (pBbA5C) alone (see Methods for details). Certain metabolites and proteins were not analyzed (black). Fold level changes represented by colored fonts (for metabolites) or colored boxes (for proteins) corresponding to the gradient shown on the right (see Additional file 2: Table S2 and Additional file 6: Table S3). CsrA targets with potential or known binding sites are depicted by a brown outline around the corresponding protein box. Pentose phosphate (penP), erythrose-4-phosphate, (E4P), glyceraldehyde-3-phosphate (G3P), 3-phosphoglycerate (3PG), phosphoenolpyruvate (PEP), pyruvate (pyr), acetyl-CoA (acCoA), oxaloacetate (oaa), citrate (cit), iso-citrate (i-cit), a-ketoglutarate (akg), glyoxylate (glx), succinyl-CoA (sucCoA), succinate (suc), malate (mal), fumarate (fum).

Intracellular concentrations of twenty-four of the thirty-four metabolites analyzed were elevated, with nine intermediates demonstrating increases greater than six-fold (Figure 2). Notably intracellular levels of lower half glycolytic intermediates glycerol-3-phosphate (G3P), 3-phosphoglycerate (3PG) and/or 2-phosphoglycerate (2PG), phosphoenolpyruvate (PEP), and pyruvate (pyr) were three- to eight-fold higher in cultures with elevated CsrB than in control cultures. Intriguingly, intracellular pools of acetyl-CoA increased eight fold. Acetyl-CoA is a vital precursor to several pathways relevant to product formation in host organisms and is likely a bottleneck to further increases in production hosts.

In contrast to the comprehensive effect observed on lower half glycolytic intermediates due to CsrB elevation, the impact on the TCA cycle appeared to be more restricted (Figure 2, Additional file 6: Table S3). We observed greater than five-fold increases in oxaloacetate (OAA), malate (MAL), fumarate (FUM), succinate (SUC), and glyoxylate in cells with elevated CsrB. Despite higher levels of these intermediates as well as acetyl-CoA, concentrations of citrate and/or isocitrate (I-CIT), and α-ketoglutarate (α-KG) were unaffected upon CsrB elevation (Figure 2, Additional file 6: Table S3).

**Figure 3:**
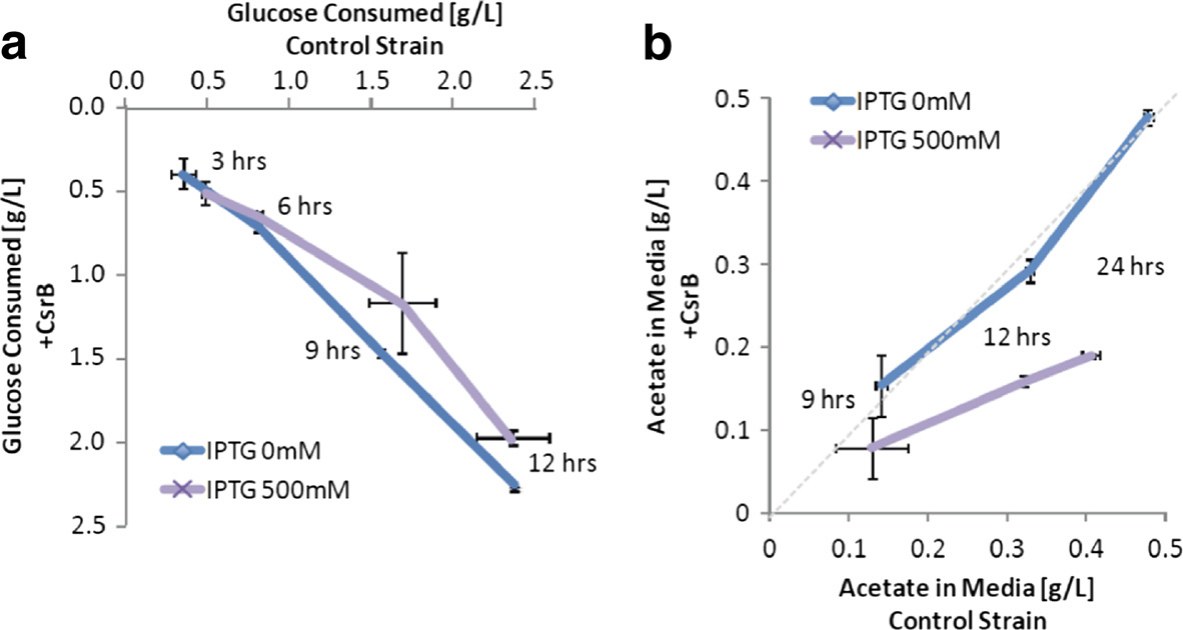
**Analysis of glucose consumption and acetate production.A**) Glucose is consumed at nearly the same rate in CsrB and control strains. **B**) We observe vastly decreasing amounts of the fermentation byproduct acetate in the growth medium in the CsrB strain. This suggests the CsrB strain is more efficient in its utilization of carbon.

We also examined levels of extracellular metabolites - glucose and acetate - in the culture medium over the course of 24-hours growth. Despite increased PEP levels, we found that CsrB induction led to a decrease in glucose consumption (Figure 3). This could not be explained by differences in cell-culture density since optical density (OD600) measurements were virtually indistinguishable between control and induced strains. In contrast, acetate, which is formed due to overflow metabolism, decreased as a function of CsrB expression (Figure 3). The decrease in extracellular acetate levels along with decreased glucose consumption levels might partly explain the carbon redistribution of intracellular metabolites described above. Additional studies on other extracellular metabolites would further shed light on the metabolic impact of CsrB, but are beyond the scope of this work. In particular, we were intrigued by the rise in intracellular glyoxylate levels upon CsrB elevation, which may have a bearing on CO_2_ secretion [28].

### CsrB-mediated alterations in amino acid levels

Increased amino acid production would be expected to benefit a host organism by increasing the ability to produce both native and heterologous proteins and hence enhance product formation. While manipulation of Csr has been previously demonstrated to increase production of phenylalanine [11,12], its impact on other amino acids has not been measured. In this study, we performed a comprehensive analysis of cellular amino-acid levels in a strain with elevated CsrB levels versus a control. Consistent with previous reports, we observed a 2.5-fold increase in phenylalanine levels. Examining the levels of other amino acids, we observed even larger changes in the levels of asparagine, threonine and aspartate which increased 47-, 30- and 9-fold, respectively (Additional file 6: Table S3).

Interestingly, from our central carbon metabolomics data we observed insignificant changes in α-KG levels. As α-KG is a TCA cycle intermediate and a precursor for the amino acids glutamate, glutamine, and proline, we would expect to see no changes in the levels of these amino acids. Surprisingly, we observed substantial increases in glutamate, glutamine, and proline levels (Additional file 6: Table S3). Additionally, amino acids derived from glycolytic intermediates that do not involve the TCA cycle (serine, glycine and alanine) were also found at higher levels as a result of CsrB elevation (Additional file 6: Table S3).

### Elevation of CsrB levels in *E. coli* improves production via engineered biofuel pathways

Our shotgun proteomics and targeted metabolomics data suggests that manipulation of Csr leads to accumulation of glycolytic and TCA cycle intermediates, decreased levels of fermentation by-products, and improved levels of both chaperones and DNA repair enzymes. These characteristics are highly desirable in the engineering of metabolic pathways. However, most strategies employed for improving compound production from metabolic pathways have primarily relied on manipulating the components of the pathways themselves [29-31]. Therefore, we wanted to test whether perturbing this global regulator rather than pathway components themselves might lead to improved production characteristics. Consequently, we tested the ability of increased CsrB levels to improve production of advanced biofuels and their precursors that have been targeted in recent metabolic engineering studies [32,33]. Specifically, we tested production from the native fatty acid (FA) pathway of *E. coli,* the n-butanol pathway (from *Clostridium acetobutylicum*), and the mevalonate pathway (from *Saccharomyces cerevisiae*).

**Figure 4:**
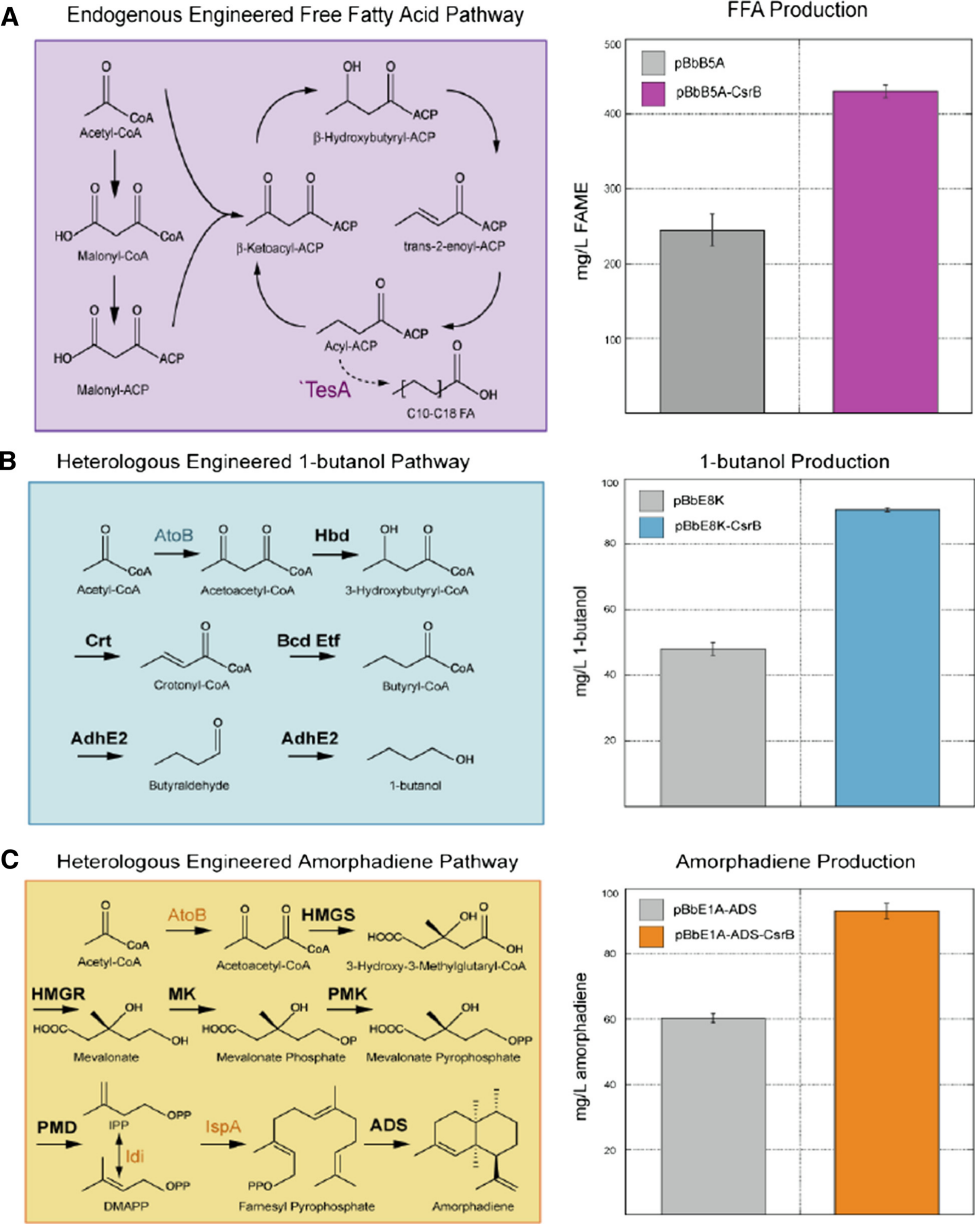
**Overexpression of CsrB improves production through engineered biofuel pathways.A**) Overexpression of CsrB improved production of total free fatty acids, detected as fatty acid methyl esters (FAMEs) by 1.8 fold in E. coli overexpressing L-tesA after 72 hrs relative to an empty plasmid control. Overexpressed E. coli genes are indicated in color. **B**) Inclusion of overexpressed CsrB nearly doubled total productionof 1-butanol yielded from E. coli co-expressing the C. acetobutylicum butanol pathway, relative to an empty plasmid control. C. acetobutylicumgenes are designated in bold font. C) CsrB overexpressed with an engineered mevalonate pathway and the amorphadiene synthase (ADS) geneproduced approximately twice the amorphadiene after 48 hrs relative to a control. Genes from S. cerevisiae and heterologous genes are designated in bold font.

FA biosynthesis commences with acetyl-CoA and malonyl-CoA as precursors followed by a series of condensation and elongation steps. Each step of the elongation cycle adds two carbons to a growing fatty acid- acyl carrier protein chain (Figure 4). Our binding site analysis suggests that translation of at least two known regulators of fatty acid metabolism, FabR and FadR, may be controlled by CsrA (Additional file 5: Table S4). FabR modulates *fabB* and *fabA* expression to balance the unsaturated:saturated FA ratio of acyl chains [34]. In contrast, the dual regulator FadR functions as a switch that coordinately regulates the machinery required for fatty acid β-oxidation and the expression of a key enzyme in FA biosynthesis [35]. FadR also represses the entire set of degradative (*fad*) genes but activates the expression of *fabA*.

Consequently, we hypothesized that Csr regulation of FabR and FadR, along with an elevated level of acetyl-CoA afforded by CsrB upregulation might impact both FA levels as well as the degree of saturation. We tested this hypothesis in a strain overexpressing a leaderless version of the *tesA* product (`TesA) with either CsrB or an empty plasmid control. Cytosolically localized `TesA can act on a range of acyl-CoA molecules to produce free fatty acids (FFAs) and FA ethyl esters [37-40]. *E. coli* overexpressing `TesA and elevated CsrB produced 76% more saturated and unsaturated FAs than control cultures bearing `TesA and an empty plasmid control (from 233 mg/L to 430 mg/L in 48 h) (Figure 4a). CsrB-mediated regulation improved total production of C12, C14, C16:1, C16, and C18:1 and C18:3 FAs, although the degree of increase was not equivalent across the FA spectrum analyzed, perhaps due to altered regulation of FabR as noted above. Notably we observed a shift from medium towards long chain FAs with levels of C16 and C18:1 and C18:3 FAs increasing approximately 3-fold which also represented a greater fraction of the total FA pool (Additional file 4: Figure S2).

Next, we examined the effect of Csr perturbation on the production of target compounds from various codon-optimized heterologous pathways. Since the heterologous pathways for n-butanol and amorphadiene production both employ acetyl-CoA as a branch-point precursor (Figure 4b and Figure 4c), we hypothesized that the enhanced acetyl-CoA pool afforded by CsrB-mediated regulation might increase production titers. n-Butanol can be produced using a four-gene pathway derived from *Clostridium acetobutylicum*[41,42], while amorphadiene is synthesized through the five-gene mevalonate pathway from *Saccharomyces cerevisiae* in conjunction with the corresponding synthase from *Artemisia annua*[43] (see Additional file 1: Table S1 for details on parts and composition)*.* We found that CsrB-mediated regulation enhanced total n-butanol production from 48 mg/L to 90 mg/L, an 88% increase, when cells were induced with 20 mM arabinose. Total production of amorphadiene increased 55% (from 60 mg/L to 93 mg/L in 48 h).

## Discussion

A proposed model of regulatory interplay connecting Csr and stringent response systems in *E. coli* involves the transcription factor DksA and the nucleotide secondary messenger ppGpp [6]. These activate *csrB/C* transcription to relieve CsrA mediated regulation during stringent response [6]. In the current study, we examined the impact of directly elevating CsrB levels thereby bypassing this native regulatory machinery. We performed a systems level analysis to assess the global impact of the aforesaid perturbation. Our proteomics and metabolomics analyses suggest that induced CsrB elevation results in the accumulation of glycolytic intermediates. In particular, we observed that CsrB-mediated deregulation of CsrA drives overexpression of the CsrA-targeted *glgCAP* operon (Figure 2), which would result in the accretion of the storage polysaccharide glycogen. Other alterations in central carbon metabolism included dramatic increases in the levels of acetyl-CoA and several amino acids, with concurrent decrease in acetate levels (Additional file 6: Table S3).

We also found that CsrB impacts the expression of the stringent response regulator DksA (Additional file 2: Table S2). DksA is responsible for transcriptional activation of CsrB (and therefore indirect deregulation of CsrA) during stringent response [6]. Additionally, we found a CsrA binding-site in the 5’UTR of DksA. This suggests that CsrB (through CsrA) directly regulates DksA, thereby forming a positive feedback loop. This model would predict that increased CsrB levels should also increase levels of DksA. In fact, we found that elevation of CsrB levels produced a 2.5-fold increase in protein levels of DksA. While a previous study [6] has shown that increased DksA expression transcriptionally activates CsrB/C, our findings strongly suggest that a post-transcriptional positive feedback loop also links CsrA/B and the stringent response regulator DksA.

Classic symptoms of the stringent response [44] such as increased amino acid biosynthesis and decreased ribosomal protein production (Additional file 2: Table S2) also appear to be amplified by CsrB overexpression. These complement typical features of the Csr system [6] such as increased carbon metabolism and motility (Additional file 2: Table S2) among others. Our findings suggest that ectopic amplification of CsrB in *E. coli* somewhat mimics the conditions of stringent response in that resources are diverted away from cellular growth and division and towards amino acid synthesis. This was evidenced by a near universal increase in amino-acid levels as exemplified by a 44-fold increase in asparagine levels. Interestingly, we also noted that CsrB elevation also significantly modulated the protein expression of the ArcA regulon (*P* < 0.005) which has previously been implicated in stringent response [45]. These results further strengthen the proposed link between Csr and the stringent response [6].

While it is possible that catabolism of isoleucine present in the medium might account for some of the improved compound production and carbon redistribution observed, we believe this effect to be marginal. In support of this, we observed an insignificant decrease in intracellular isoleucine levels (1.4-fold, *P* ~ 0.1) between test and control strains. Additionally, we observed no evidence for changing protein levels or CsrA binding sites among genes involved in protein recycling or proteolysis.

## Conclusions

Given the ubiquity of engineered pathways that rely on glycolysis for producing biologically-derived compounds, results from this study would be expected to have broad implications. Indeed, we demonstrated improved hydrocarbon production through three distinct routes engineered for generating advanced biofuels by simple manipulation of this ribonucleoprotein regulatory scheme in *E. coli*. While it is tempting to speculate that the increased amino acid levels due to CsrB elevation may also be used to improve biofuel production through synthetic non-fermentative pathways [46], such validation remains to be performed.

This approach obviates the need for extensive host organism modification in simply requiring overexpression of a single non-coding RNA. It remains to be seen if such an approach can be applied synergistically with other approaches for improving production. As such, this study shows that targeting of the carbon storage regulator and stringent response systems can be a powerful means with which to increase targeted compound production in metabolic engineering applications. This approach is likely generalizable to other pathways and organisms.

## Materials and methods

### Host strain

*E. coli* DH5a (F^-^ endA1 glnV44 thi-1 recA1 relA1 gyrA96 deoRnupG Φ80d*lacZ*ΔM15 Δ(*lacZYA-argF*)U169, hsdR17(r_K_^-^m_K_^+^), λ–) was used for cloning and DNA amplification. *E. coli* strain BLR (Δ*tyrR*; Δ*pheA/L*; *aroF*[P148L]; *tyrA*[M53I; A354V]) [36,47,48] was used as the base strain (BLR-DAJ) in this study. This strain is auxotrophic for phenylalanine and isoleucine [49].

### Plasmids for advanced biofuel production

CsrB was amplified from *E. coli* MG1655 using primers CsrB_F/R. Restriction enzymes BamHI and XhoI were used to insert CsrB into the medium copy vectors pBbA5A and pBbB5C, with the lacUV5 promoter (P_lacUV5_) and the chloramphenicol or ampicillin resistance genes, respectively. The *C. acetobutylicum* butyryl-CoA biosynthetic operon (*crthbdetfABhbd*) and alcohol dehydrogenase (*adhE2)* were amplified from *C. acetobutylicum* ATCC824 genomic DNA (ATCC) using primers F76/F73 and F72/F74, respectively. The vector backbone (containing a p15a origin), chloramphenicol selective marker, trc promoter (P_trc_), and LacI^Q^ was amplified using primers F75/F77. The three PCR products were assembled using the SLIC protocol [50], producing plasmid pBMO49. The *atoB* gene from *E. coli* (PCR amplified using primers F92/F93) and an additional P_trc_ promoter (PCR amplified using primers F90/F91) were inserted 5’ of *adhE2* to generate plasmid pBMO50 using BglII and BamHI restriction sites. Plasmids for fatty acid production [37] and amorphadiene production [30] and the respective extraction procedures are described elsewhere.

### Medium composition and growth conditions

Individual colonies were grown overnight at 37°C in LB medium and passed 1:100 for an additional night of growth in Neidhardt MOPS medium containing 0.5 or 1.0% glucose for metabolite analysis or heterologous pathway production experiments, respectively. Glucose was the sole carbon source used in all production experiments. All metabolite production experiments were carried out in defined media (Neidhardt MOPS) [51] supplemented with 12.5 g/L [49] and 10.0 g/L phenylalanine. *E. coli* were seeded at 1:100 dilutions into either 5-mL shake tubes or 50-ml shake flasks (metabolomics experiments) and grown at 30°C or 37°C with appropriate antibiotics. IPTG (Sigma-Aldrich) was added at the concentrations indicated to induce CsrB. Except where noted, all metabolite and production data were collected at 48 hrs after induction. All data presented are the averaged results of biological triplicates.

### Proteomics sample preparation & analysis

Cells were centrifuged at 8000 × *g* at 4°C and frozen in liquid nitrogen. Protein extraction was performed using chloroform/methanol (Wessel and Flugge, 1984) followed by desiccation in a vacuum concentrator (ThermoSavant). Samples were reconstituted in 200 μL of 100 mM ammonium bicarbonate with 20% (v/v) methanol to a final concentration of 0.25 μg/μL. Disulfide bonds were reduced with 5 mM TCEP for 30 minutes, and alkylation was performed with 200 mM iodoacetic acid for 30 minutes in the dark. Trypsin (1 μg/μL) was added to a final concentration of 1:50 (trypsin:sample), and incubated at 37°C overnight.

Samples were washed for 10 min at a 15 μL/min flow rate with a buffer consisting of 94% (v/v) acetonitrile, 0.1% (v/v) formic acid and 5.9% H_2_O on a Pepmap100 μ-guard column (Dionex-LC Packings, Sunnyvale, CA). Samples were then applied to a Pepmap100 analytical column (75-μm i.d., 150-mm length, 100 Å, and 3 μm) for 60 minutes at a flow rate of 300 nL/min. Column pressure was equilibrated with buffer for two minutes. Samples were analyzed on a LC-MS/MS system consisting of an Eksigent TEMPO nanoLC-2D coupled to an AB Sciex (Foster City, CA) 5600 Triple-TOF mass spectrometer running Analyst TF™ 1.5.1 (AB Sciex) in IDA experiment mode.

Spectra were processed using PeakView v1.1.1.2 (AB Sciex), analyzed with Mascot 2.2 (Matrix Science) and imported in Scaffold v3.2.0 (Proteome Software Inc, Portland, OR USA) for analysis. A database of all *E. coli* ORFs (MicrobesOnline) was searched using a peptide tolerance of 100 ppm, allowing for 1 missed trypsin cleavage and carboxymethyl modifications. Proteins were identified with at least two 95% confidence peptides. For analysis of fold changes, only proteins with at least five spectral counts were considered.

### Sampling, quenching, and extraction of metabolites

Separation of metabolites for the analysis of glycolysis and TCA cycle intermediates was conducted on a fermentation monitoring HPX-87 H column with 8% cross linkage (150-mm length, 7.8-mm internal diameter, and 9-μm particle size; Biorad, CA, USA). Pyruvate separation was conducted on a ZIC-HILIC column (250-mm length, 2.1-mm internal diameter, and 3.5-μm particle size; Merck SeQuant, MA, USA). Samples were run on an Agilent Technologies 1100 Series HPLC (Agilent Technologies, CA, USA).

For analysis of amino acids, CE separations were performed in a 100 cm, 50-*μ*m i.d. ~365-*μ*m o.d. (total volume 1963 nL), untreated, fused-silica capillary (PolyMicro Technologies) via the Agilent CE system (Agilent Technologies). Details on subsequent experimental protocols are provided in the Supplemental Methods.

### Motif analyses and statistical testing

Using experimentally determined CsrA binding sites from previous studies [23,52-55] and our proteomics results, we identified genome wide targets putatively regulated by CsrA. Details of this motif analyses are provided in Supplemental Methods. Tests for statistical enrichment of motifs and functional categories were performed using the hypergeometric enrichment test [56]. Multiple-test correction of test statistics was performed using the Benjamini-Hochberg method [57]. Gene ontology annotations were obtained from AMIGO [58] while TIGR gene indices were obtained from Microbes Online [25].

## Abbreviations

Csr, Carbon storage regulator; SD, Shine-Delgarno; Bp, Base pair; FA, Fatty acid; FAME, Fatty acid methyl ester; ADS, Amorphadiene synthase; GO, Gene ontology; TIGR, The Institute for Genomic Research; CE, Capillary electrophoresis; PCR, Polymerase chain reaction; RNA, Ribonucleic acid; DNA, Deoxyribonucleic acid; ORF, Open reading frame; E. coli, Escherichia coli; UTR, Untranslated region.

## Competing interests

JDK has financial interests in Amyris and LS9, both of which are involved in producing advanced biofuels.

## Author contributions

Experimental work was performed by AEM, BJR, DCC, EKB, DJ, DK, PIB, JAD, and SMM. DK, DCC and CJP analyzed the data. APA, CJP, APA CJP, PDA, JDK and SRC supervised the study. DK and SRC wrote the final manuscript. All authors read and approved the final manuscript.

## Supplementary Material

Additional file 4 **Figure S1.** A) Interaction between Csr and the stringent response [6]. B) CsrA RNA-binding motifs. *P*-values refer to statistical enrichment (hypergeometric enrichment test) of motifs among proteins with changing expression. C) Sensitivity and Specificity (Harmonic mean) of various motifs. **Figure S2. Analysis of the harmonic mean of precision and recall (F**_**1**_**-measure) of various motifs. Figure S3. Overexpression of CsrB alters the distribution of free fatty acids towards longer chain fatty acids.** Cultures of *E. coli* co-expressing the L-tesA gene and either CsrB or an empty plasmid control were analyzed for production of saturated and unsaturated FAs of lengths 12-18 yielded after induction and growth for 72 hrs in Neidhardt MOPS minimal medium. Inclusion of CsrB leads to an improvement in total production. Shown here is the percentage that each medium and long chain FA contributes to total FA production. Overexpression of CsrB alters the distribution of production towards longer chain FAs, with a decrease seen in C12 and an increase in C14, C16, and unsaturated C18 FAs. FAs were derivatized to FAMEs for GC-FID analysis. Click here for file

Additional file 1 **Table S1.** Plasmids and Primers Used in this Study. Click here for file

Additional file 2 **Table S2.** Whole cell proteomic analysis for CsrB elevation in *E. coli.* Click here for file

Additional file 3 **Table S5.** Whole---cell proteomics analysis. Click here for file

Additional file 6 **Table S3.** Metabolites impacted by CsrB overexpression. Click here for file

Additional file 5 **Table S4.** Predicted CsrA binding sites. Click here for file
