# Supplementary material for: Manipulation of the carbon storage regulator system for metabolite remodeling and biofuel production in Escherichia coli
Source: Microb Cell Fact. 2012 Jun 13;11:79. doi: 10.1186/1475-2859-11-79 (PMC3460784; doi:10.1186/1475-2859-11-79)
Supplement: Additional file 4 — Figure S1. A) Interaction between Csr and the stringent response [6]. B) CsrA RNA-binding motifs. P-values refer to statistical enrichment (hypergeometric enrichment test) of motifs among proteins with changing expression. C) Sensitivity and Specificity (Harmonic mean) of various motifs. Figure S2. Analysis of the harmonic mean of precision and recall (F1-measure) of various motifs. Figure S3. Overexpression of CsrB alters the distribution of free fatty acids towards longer chain fatty acids. Cultures of E. coli co-expressing the L-tesA gene and either CsrB or an empty plasmid control were analyzed for production of saturated and unsaturated FAs of lengths 12-18 yielded after induction and growth for 72 hrs in Neidhardt MOPS minimal medium. Inclusion of CsrB leads to an improvement in total production. Shown here is the percentage that each medium and long chain FA contributes to total FA production. Overexpression of CsrB alters the distribution of production towards longer chain FAs, with a decrease seen in C12 and an increase in C14, C16, and unsaturated C18 FAs. FAs were derivatized to FAMEs for GC-FID analysis. [file 1475-2859-11-79-S4.pdf]

Table S3. Metabolites impacted by CsrB overexpression.

Glycolysis Metabolite	Fold Change	Std. Dev.
Glyceraldehyde-3-phosphate	7.43	2.97
3Phosphoglycerate	4.65	0.70
Phosphoenolpyruvate	2.89	0.50
Pyruvate	4.37	1.08
Acetyl-CoA	7.62	0.67
G1P+G6P+F6P	3.32	0.61
**TCA Metabolite**	**Fold Change**	**Std. Dev.**
Oxaloacetate	6.36	1.93
Malate	11.22	3.59
Fumarate	5.27	3.91
Succinate	5.51	1.59
Oxoglutarate	0.80	0.10
cis-Aconitate	0.69	0.07
Citrate/Isocit	0.65	0.09
Glyoxylate	3.60	0.82
**Amino Acid**	**Fold Change**	**Std. Dev.**
Glycine	2.82	0.65
Alanine	7.89	0.94
Serine	3.18	0.95
Proline	6.15	3.11
Betaine	2.18	0.59
Valine	0.80	0.11
Threonine	28.30	5.83
Cysteine	0.48	0.05
Leucine	0.93	0.22
Isoleucine	0.70	0.14
Asparagine	44.42	19.29
Aspartate	8.41	2.52
Glutamine	15.25	9.15
Glutamate	13.58	8.13
Methionine	1.05	0.32
Phenylalanine*	2.51	0.95
Tyrosine	1.88	0.23
Tryptophan	4.87	2.88
Lysine	3.57	1.30
Histidine	3.80	2.25
Arginine	0.66	0.26

* An increase in phenylalanine (despite the complete deletion of pheA/L in BLR-DAJ) is attributed to accumulated prephenate which can be non-enzymatically converted to phenylpyruvate and transaminated to phenylalanine[a].

[a] Olson, M. M., et al., 2007. Production of tyrosine from sucrose or glucose achieved by rapid genetic changes to phenylalanine-producing *Escherichia coli* strains. Applied microbiology and biotechnology. 74, 1031-40.
