# Supplementary figures and images for: Manipulation of the carbon storage regulator system for metabolite remodeling and biofuel production in Escherichia coli
Source: Microb Cell Fact. 2012 Jun 13;11:79. doi: 10.1186/1475-2859-11-79 (PMC3460784; doi:10.1186/1475-2859-11-79)

Figure S1.

A.

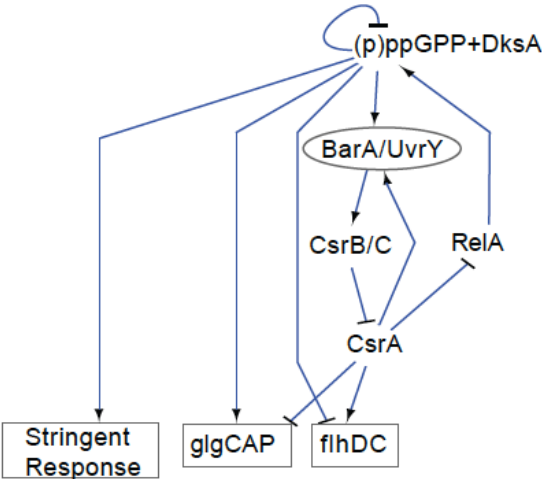

B.

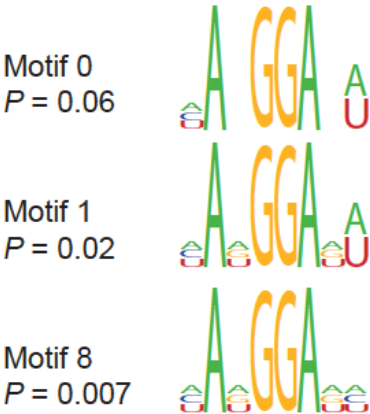

C.

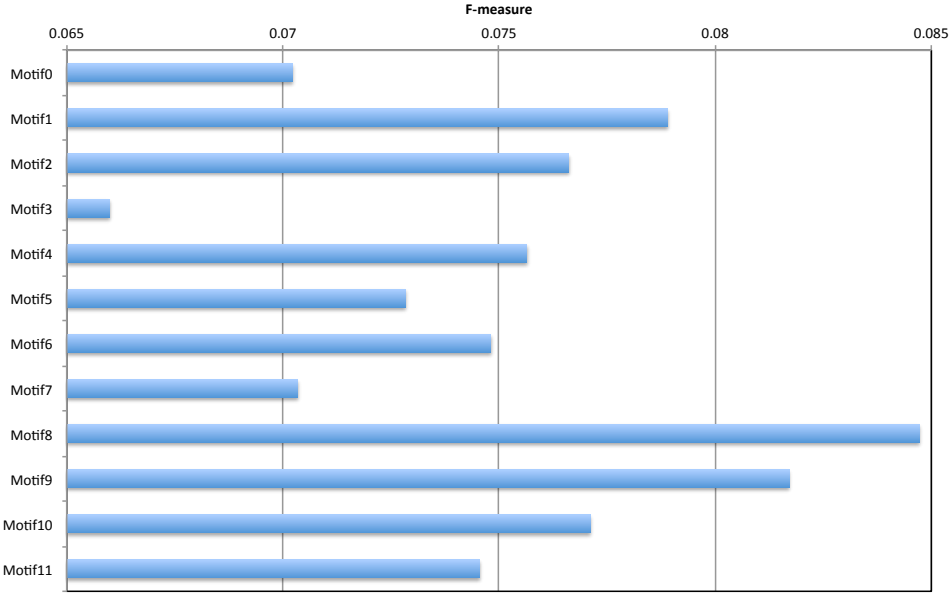

Figure S2.

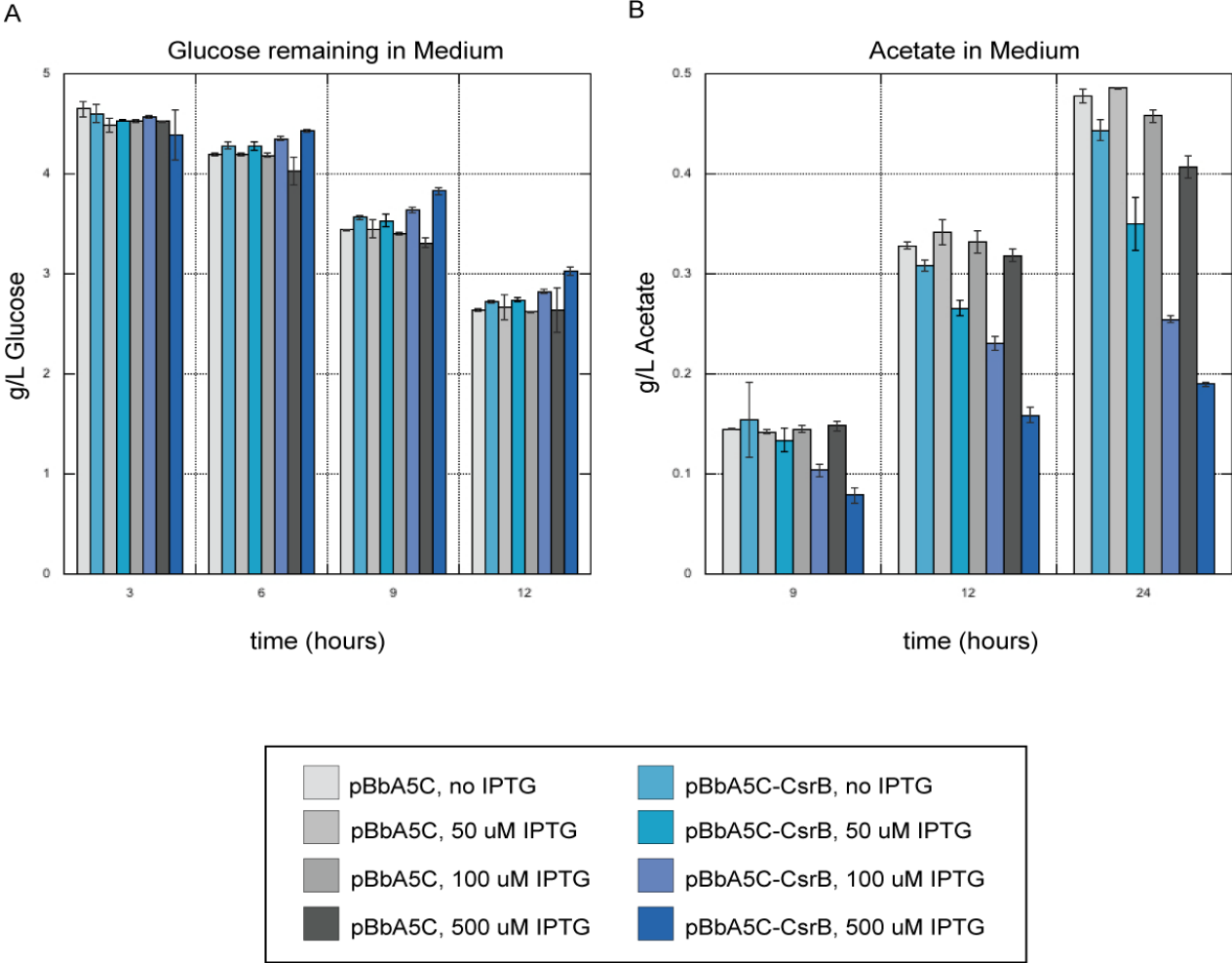

**Figure S3.**

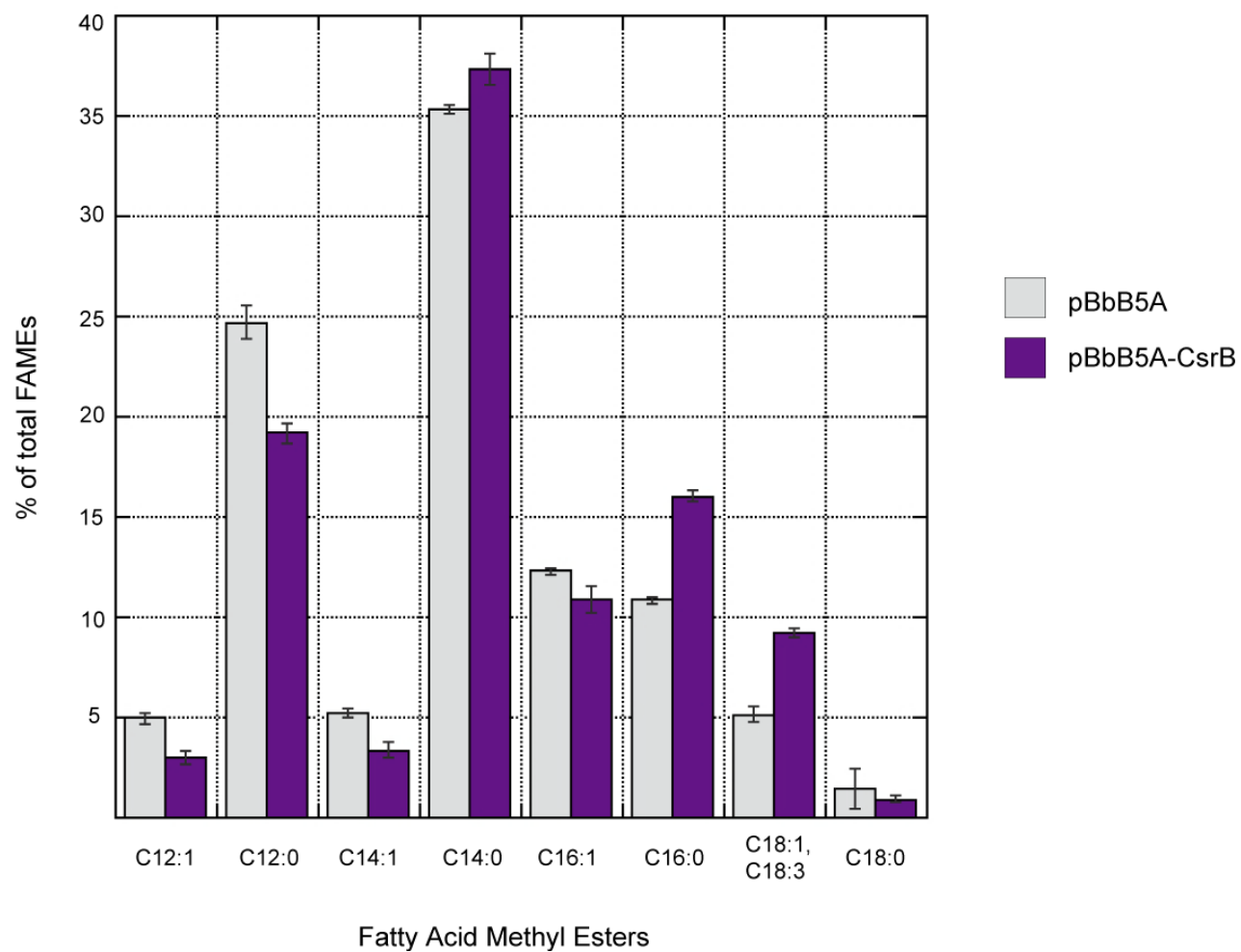

Supplement: Additional file 1 — Table S1. Plasmids and Primers Used in this Study. [file 1475-2859-11-79-S1.pdf]
