# Supplementary material for: Manipulation of the carbon storage regulator system for metabolite remodeling and biofuel production in Escherichia coli
Source: Microb Cell Fact. 2012 Jun 13;11:79. doi: 10.1186/1475-2859-11-79 (PMC3460784; doi:10.1186/1475-2859-11-79)
Supplement: Additional file 2 — Table S2. Whole cell proteomic analysis for CsrB elevation in E. coli. [file 1475-2859-11-79-S2.pdf]

Table S1. Plasmids and Primers Used in this Study

Name	Description	Reference
pBbA5C	placUV5 promoter/p15A origin/CmR	This study.
pBbS8K	pBAD promoter/pSC101 origin/KanR	This study.
pBbE8K	pBAD promoter/colE1 origin/KanR	This study.
pBbB5A	placUV5 promoter/MMR origin/AmpR	This study.
pBbE1A-ADS	pTrc promoter/ColE1 origin/AmpR bearing ADS gene	This study.
pKS1	placUV5 promoter/p15A origin/CmR	(Steen *et al*, 2010)
pBMO50	placUV5 promoter/p15A origin/CmR	This study.
pBbA5C-MevT/B	placUV5 promoter/p15A origin/CmR	This study.
pBbA5C-CsrB	bearing the CsrB gene cloned with BamH1 and XhoI	This study.
pBbS8K-CsrB	bearing the CsrB gene cloned with EcoR1 and XhoI	This study.
pBbE8K-CsrB	bearing the CsrB gene cloned with EcoR1 and XhoI	This study.
pBbB5A-CsrB	bearing the CsrB gene cloned with EcoR1 and XhoI	This study.
pBbB5A-ADS-CsrB	bearing an operon consisting of a terminator and placUV5-CsrB cassette	This study.

Name	Primer Sequence	Description
csrB_F	tcagaattcggatccGAGTCAGACAACGAAGTGAAC	CsrB 5’ F
csrB_R	tggctcgagAATAAAAAAAGGGAGCACTGTATTCA	CsrB 3’ R
F72	attaatacgcgagttaaggagtacaggagcatgaaggtgaccaatcagaaagaactgaa	AdhE forward with junction on butanol pathay in pButE1:v1.0
F73	tactccttaactcgcgtattaatttggatccttattttgaataatcgtagaaaccttttc	AdhE reverse with junction on butanol pathay in pButE1:v1.0
F74	atctacattaagagcttttgaagatctgaagcttgggcccgaacaaaaac	AdhE 3' end forward with trrnB on pButE1:v1.0
F75	gtttttgttcgggcccaagcttcagatcttcaaaagctcttaatgtagat	AdhE 3' end reverse with trrnB on pButE1:v1.0
F76	ttgtgagcggataacaatttcacaccagcaggacgcactgagggcccatg	pTRC-Butanol 5' F
F77	catgggccctcagtgcgtcctgctggtgtgaaattgttatccgctcacaa	pTRC-Butanol 5' R
F90	tttctacgattattcaaaataaggatcccgactgcacggtgcaccaatgcttctggcg	SLIC primer- Forward on pTRC (hbd/pTRC)
F91	acctacctccttatttcaatttttcgatgaattgttatccgctcacaattccacacatt	SLIC primer - Reverse on pTRC (pTRC/AtoB)
F92	aaaattgaaataaggaggtaggtagtaatgaaaaattgtgtcatcgtcagtgcggtacg	SLIC primer - Forward on AtoB (pTRC/AtoB)
F93	tgtactccttaactcgcgtattaatttgttaattcaaccgttcaatcaccatcgcaat	SLIC primer - Reverse on AtoB (AtoB/AdhE2)
