# Supplementary material for: Manipulation of the carbon storage regulator system for metabolite remodeling and biofuel production in Escherichia coli
Source: Microb Cell Fact. 2012 Jun 13;11:79. doi: 10.1186/1475-2859-11-79 (PMC3460784; doi:10.1186/1475-2859-11-79)
Supplement: Additional file 3 — Table S5. Whole---cell proteomics analysis. [file 1475-2859-11-79-S3.pdf]

Table S2: Whole cell proteomic analysis for CsrB elevation in *E. coli*.

Protein ID	Description	Spectral Counts							TIGR Roles	TIGR Family	TIGR Description
		Induced (Avg)	Stdev	Uninduced (Avg)	Stdev	Fold Change	Variance	Log_2 (Fold Change)			
**GlgB**	glycogen branching enzyme	14.03	1.06	0.84	0.01	16.71	0.01	4.06	Energy metabolism:E	TIGR01515 1,4	Biosynthesis and degradation of polysaccharides
**CysA**	sulfate/thiosulfate transporter subunit	5.82	2.05	0.84	0.01	6.93	0.12	2.79	Transport and bindir	TIGR00968 sul	Anions
**YhiI**	predicted HlyD family secretion protein	9.75	4.11	1.96	0.49	4.97	0.24	2.31	N/A	N/A	N/A
**GlgC**	glucose-1-phosphate adenylyltransferase	16.43	0.89	3.65	0.51	4.50	0.02	2.17	Energy metabolism:E	TIGR02091 glu	Biosynthesis and degradation of polysaccharides
**IaaA**	L-asparaginase	5.45	2.30	1.69	0.86	3.23	0.44	1.69	N/A	N/A	N/A
**TolC**	outer membrane channel; specific toleran	11.48	1.16	3.65	0.51	3.15	0.03	1.65	Transport and bindir	TIGR01844 typ	Porins
**AhpC**	alkyl hydroperoxide reductase, C22 subun	52.02	4.83	17.6	5.68	2.96	0.11	1.56	Cellular processes:Ad	TIGR03137 per	Adaptations to atypical conditions
**OmpA**	outer membrane protein A	183.08	17.70	64.36	11.33	2.84	0.04	1.51	N/A	N/A	N/A
**PspA**	regulatory protein for phage-shock-protei	24.60	7.50	8.69	1.35	2.83	0.12	1.50	Cellular processes:Ad	TIGR02977 pha	Adaptations to atypical conditions
**DksA**	DNA-binding transcriptional regulator of r	9.46	2.95	3.64	0.5	2.60	0.12	1.38	Regulatory functions	TIGR02420 RN	Small molecule interactions
**RraA**	ribonuclease activity regulator protein Rra	11.43	3.35	4.47	1.23	2.56	0.16	1.35	Regulatory functions	TIGR01935 Rra	Protein interactions
**GrpE**	heat shock protein	7.43	0.50	3.07	1.26	2.42	0.17	1.28	N/A	N/A	N/A
**Pck**	phosphoenolpyruvate carboxykinase	21.24	5.81	8.96	1.26	2.37	0.09	1.25	Energy metabolism:G	TIGR00224 pho	Glycolysis/gluconeogenesis
**CysN**	sulfate adenylyltransferase subunit 1	26.17	8.14	12.04	1.3	2.17	0.11	1.12	Central intermediary	TIGR00231 sm	Sulfur metabolism
**RplR**	50S ribosomal protein L18	5.47	2.88	2.52	0.85	2.17	0.39	1.12	Protein synthesis:Rib	TIGR00060 ribo	Ribosomal proteins
**SelD**	selenophosphate synthetase	7.25	3.95	3.35	0.8	2.16	0.35	1.11	N/A	TIGR00476 sel	N/A
**FucU**	L-fucose mutarotase	5.40	1.09	2.52	0.82	2.14	0.15	1.10	N/A	N/A	N/A
**SecB**	export protein SecB	16.38	2.05	7.85	2.17	2.09	0.09	1.06	Protein fate:Protein	TIGR00809 pro	Protein and peptide secretion and trafficking
**AllR**	DNA-binding transcriptional repressor	5.71	1.67	2.8	0.5	2.04	0.12	1.03	N/A	N/A	N/A
**YfgM**	hypothetical protein	11.29	4.12	5.6	1	2.02	0.17	1.01	N/A	N/A	N/A
**CysD**	sulfate adenylyltransferase subunit 2	7.34	1.76	3.64	0.5	2.02	0.08	1.01	Central intermediary	TIGR02039 sul	Sulfur metabolism
**SdhB**	succinate dehydrogenase, FeS subunit	18.23	2.67	9.25	0.92	1.97	0.03	0.98	Energy metabolism:T	TIGR00384 suc	TCA cycle
**Gst**	glutathionine S-transferase	22.87	5.54	11.76	0.14	1.94	0.06	0.96	N/A	N/A	N/A
**UspA**	universal stress global response regulator	43.04	14.24	22.4	1.13	1.92	0.11	0.94	N/A	N/A	N/A
**HflC**	modulator for HflB protease specific for ph	16.14	2.16	8.41	1.53	1.92	0.05	0.94	Regulatory functions	TIGR01932 Hfl	Protein interactions
**Ssb**	single-strand DNA-binding protein	5.31	1.15	2.8	0.5	1.90	0.08	0.92	DNA metabolism:DN	TIGR00621 sin	DNA replication, recombination, and repair
**YiiU**	hypothetical protein	7.38	0.75	3.91	0.92	1.89	0.07	0.92	N/A	N/A	N/A
**ZapA**	protein that localizes to the cytokinetic rin	5.77	1.83	3.09	0.52	1.87	0.13	0.90	N/A	N/A	N/A
**ManZ**	PTS enzyme IID, mannose-specific	6.27	2.43	3.36	0.84	1.87	0.21	0.90	Signal transduction:P	TIGR00828 PTS	PTS
**GlgA**	glycogen synthase	9.38	2.38	5.04	2.25	1.86	0.26	0.90	Energy metabolism:E	TIGR02095 gly	Biosynthesis and degradation of polysaccharides
**b2253**	#N/A	15.66	0.46	8.42	1.77	1.86	0.05	0.89	#N/A	#N/A	N/A
**PntB**	pyridine nucleotide transhydrogenase	10.74	1.12	5.87	1.38	1.83	0.07	0.87	N/A	N/A	N/A
**MinD**	membrane ATPase of the MinC-MinD-Min	22.34	3.22	12.32	0.46	1.81	0.02	0.86	Cellular processes:Ce	TIGR01968 sep	Cell division
**YaeP**	hypothetical protein	7.10	2.33	3.93	0.53	1.81	0.13	0.85	N/A	N/A	N/A
**TpiA**	triosephosphate isomerase	10.12	4.16	5.61	1.34	1.80	0.23	0.85	Energy metabolism:G	TIGR00419 trio	Glycolysis/gluconeogenesis
**MinE**	cell division topological specificity factor M	5.40	1.09	3.07	1.26	1.76	0.21	0.81	Cellular processes:Ce	TIGR01215 cell	Cell division
**YiaD**	predicted outer membrane lipoprotien	5.88	3.01	3.35	1.64	1.76	0.50	0.81	N/A	N/A	N/A
**PhoP**	DNA-binding response regulator in two-co	12.00	3.12	7	1.75	1.71	0.13	0.78	N/A	N/A	N/A
**SucD**	succinyl-CoA synthetase subunit alpha	31.89	5.92	18.77	1.19	1.70	0.04	0.76	Energy metabolism:T	TIGR01019 suc	TCA cycle
**CysM**	cysteine synthase B (O-acetylserine sulfhy	6.18	1.13	3.64	0.5	1.70	0.05	0.76	Amino acid biosynth	TIGR01136 cys	Serine family
**YhcB**	orf, hypothetical protein	20.72	4.16	12.32	1.9	1.68	0.06	0.75	N/A	N/A	N/A
**SurA**	peptidyl-prolyl cis-trans isomerase (PPIase	14.55	3.54	8.68	0.53	1.68	0.06	0.75	N/A	N/A	N/A
**TolB**	translocation protein TolB precursor	11.68	3.71	7	0.95	1.67	0.12	0.74	Cellular processes:Pa	TIGR02800 Tol	Pathogenesis
**YbiC**	predicted dehydrogenase	7.47	2.59	4.48	1.29	1.67	0.20	0.74	N/A	N/A	N/A
**HflK**	modulator for HflB protease specific for ph	13.03	3.84	7.84	0.55	1.66	0.09	0.73	N/A	TIGR01933 Hfl	N/A
**IhfB**	integration host factor subunit beta	11.19	2.73	6.73	1.49	1.66	0.11	0.73	DNA metabolism:DN	TIGR00988 inte	DNA replication, recombination, and repair
**MraW**	S-adenosyl-methyltransferase	11.61	3.04	7	1.32	1.66	0.10	0.73	Cell envelope:Biosyn	TIGR00006 S-a	Biosynthesis and degradation of murein sacculus and peptidoglycan
**YfgL**	protein assembly complex, lipoprotein cor	10.80	3.38	6.73	5.15	1.60	0.68	0.68	Protein fate:Protein	TIGR03300 out	Protein and peptide secretion and trafficking
**FabI**	enoyl-(acyl carrier protein) reductase	25.11	2.61	15.67	1.95	1.60	0.03	0.68	N/A	N/A	N/A
**FabD**	acyl carrier protein S-malonyltransferase	8.97	2.04	5.6	1	1.60	0.08	0.68	Fatty acid and phosp	TIGR00128 ma	Biosynthesis
**MscS**	mechanosensitive channel	9.87	0.98	6.18	1.82	1.60	0.10	0.68	N/A	N/A	N/A
**TktA**	transketolase 1, thiamin-binding	54.84	2.05	34.47	2.9	1.59	0.01	0.67	Energy metabolism:P	TIGR00232 tra	Pentose phosphate pathway
**Crp**	DNA-binding transcriptional dual regulator	14.22	4.32	8.97	1.04	1.58	0.11	0.66	N/A	N/A	N/A
**PepB**	putative peptidase	18.99	1.34	12.04	0.89	1.58	0.01	0.66	N/A	N/A	N/A
**RplL**	50S ribosomal protein L7/L12	53.28	8.55	33.87	3.13	1.57	0.03	0.65	Protein synthesis:Rib	TIGR00855 ribo	Ribosomal proteins
**CspC**	stress protein, member of the CspA-family	11.46	2.07	7.29	1.36	1.57	0.07	0.65	N/A	N/A	N/A
**YfgA**	hypothetical protein	6.14	0.84	3.92	0.47	1.57	0.03	0.65	N/A	N/A	N/A

**YaeT**	hypothetical protein	13.11	1.76	8.41	1.49	1.56	0.05	0.64	Protein fate:Protein	TIGR03303 out	Protein and peptide secretion and trafficking
**YbiS**	hypothetical protein	8.29	3.79	5.33	1.33	1.55	0.27	0.64	N/A	N/A	N/A
**LacI**	transcriptional repressor of the lac operon	38.62	7.59	25.23	3.19	1.53	0.05	0.61	N/A	N/A	N/A
**SdhA**	succinate dehydrogenase flavoprotein sub	36.03	6.02	23.8	0.83	1.51	0.03	0.60	Energy metabolism:T	TIGR01812 suc	TCA cycle
**TalB**	transaldolase B	38.60	2.36	25.51	3.45	1.51	0.02	0.60	Energy metabolism:P	TIGR00874 tra	Pentose phosphate pathway
**BasR**	DNA-binding response regulator in two-co	4.99	1.52	3.37	0.87	1.48	0.16	0.57	N/A	N/A	N/A
**Lrp**	DNA-binding transcriptional dual regulato	7.45	2.29	5.03	1.42	1.48	0.17	0.57	N/A	N/A	N/A
**UbiE**	ubiquinone/menaquinone biosynthesis me	5.40	1.09	3.65	0.51	1.48	0.06	0.57	Biosynthesis of cofac	TIGR01934 ubi	Menaquinone and ubiquinone
**IscU**	scaffold protein	8.67	0.59	5.87	1.62	1.48	0.08	0.56	Biosynthesis of cofac	TIGR01999 FeS	Other
**NlpA**	cytoplasmic membrane lipoprotein-28	6.18	1.13	4.19	2.19	1.47	0.31	0.56	Cell envelope:Other	TIGR00363 lipo	Other
**CysH**	phosphoadenosine phosphosulfate reduct	23.96	2.38	16.26	1.45	1.47	0.02	0.56	Central intermediary	TIGR00434 pho	Sulfur metabolism
**Icd**	isocitrate dehydrogenase	117.18	3.60	79.82	1.42	1.47	0.00	0.55	Energy metabolism:T	TIGR00183 iso	TCA cycle
**HemX**	predicted uroporphyrinogen III methylase	9.04	1.70	6.17	1.04	1.47	0.06	0.55	N/A	N/A	N/A
**CyoA**	cytochrome o ubiquinol oxidase subunit II	10.24	1.23	6.99	1.2	1.46	0.04	0.55	Energy metabolism:E	TIGR01433 ubi	Electron transport
**YhbS**	predicted acyltransferase with acyl-CoA N-	5.33	1.66	3.64	0.44	1.46	0.11	0.55	N/A	N/A	N/A
**InfA**	translation initiation factor IF-1	5.31	1.15	3.65	0.51	1.45	0.07	0.54	Protein synthesis:Tra	TIGR00008 tra	Translation factors
**YjgF**	orf, hypothetical protein	6.49	2.20	4.49	1.01	1.45	0.17	0.53	Transcription:Degrad	TIGR00004 put	Degradation of RNA
**AtpG**	F0F1 ATP synthase subunit gamma	16.60	3.05	11.49	1.03	1.44	0.04	0.53	Energy metabolism:A	TIGR01146 ATP	ATP-proton motive force interconversion
**RplK**	50S ribosomal protein L11	12.85	3.10	8.96	0.46	1.43	0.06	0.52	Protein synthesis:Rib	TIGR01632 ribo	Ribosomal proteins
**IspG**	4-hydroxy-3-methylbut-2-en-1-yl diphosph	12.72	1.09	8.96	0.46	1.42	0.01	0.51	Biosynthesis of cofac	TIGR00612 4-h	Other
**SucA**	alpha-ketoglutarate decarboxylase	53.90	10.07	38.07	1.67	1.42	0.04	0.50	Energy metabolism:T	TIGR00239 oxo	TCA cycle
**YcfF**	orf, hypothetical protein	6.67	2.33	4.75	1.24	1.40	0.19	0.49	N/A	N/A	N/A
**UspE**	stress-induced protein	22.79	3.48	16.25	2.13	1.40	0.04	0.49	N/A	N/A	N/A
**MetH**	B12-dependent methionine synthase	30.17	7.27	21.57	3	1.40	0.08	0.48	Amino acid biosynth	TIGR02082 me	Aspartate family
**InfC**	translation initiation factor IF-3	26.92	6.04	19.32	2.97	1.39	0.07	0.48	Protein synthesis:Tra	TIGR00168 tra	Translation factors
**TyrS**	tyrosyl-tRNA synthetase	23.37	7.15	16.77	4.51	1.39	0.17	0.48	Protein synthesis:tRN	TIGR00234 tyr	tRNA aminoacylation
**CysI**	sulfite reductase, beta subunit, NAD(P)-bin	43.71	1.68	31.38	5.81	1.39	0.04	0.48	Central intermediary	TIGR02041 sul	Sulfur metabolism
**Pgm**	phosphoglucomutase	19.49	4.87	14.01	1.08	1.39	0.07	0.48	Energy metabolism:S	TIGR01132 pho	Sugars
**DapA**	dihydrodipicolinate synthase	12.46	3.17	8.96	1.01	1.39	0.08	0.48	Amino acid biosynth	TIGR00674 dih	Aspartate family
**FumA**	fumarate hydratase (fumarase A), aerobic	21.64	6.88	15.7	2.27	1.38	0.12	0.46	N/A	TIGR00722 hyd	N/A
**AspC**	aspartate aminotransferase, PLP-depende	64.81	5.49	47.06	2.55	1.38	0.01	0.46	N/A	N/A	N/A
**ManX**	fused mannose-specific PTS enzymes: IIA c	17.31	1.02	12.63	3.16	1.37	0.07	0.45	Signal transduction:P	TIGR00824 PTS	PTS
**NlpB**	lipoprotein-34	9.94	1.80	7.28	2.11	1.37	0.12	0.45	N/A	N/A	N/A
**SucB**	dihydrolipoamide acetyltransferase	48.15	2.74	35.27	3.03	1.37	0.01	0.45	Energy metabolism:T	TIGR01347 dih	TCA cycle
**RpsF**	30S ribosomal protein S6	15.99	3.79	11.76	1.38	1.36	0.07	0.44	Protein synthesis:Rib	TIGR00166 ribo	Ribosomal proteins
**Ftn**	ferritin iron storage protein (cytoplasmic)	9.87	2.26	7.27	1.21	1.36	0.08	0.44	N/A	N/A	N/A
**SucC**	succinyl-CoA synthetase subunit beta	59.71	10.49	43.99	3.82	1.36	0.04	0.44	Energy metabolism:T	TIGR01016 suc	TCA cycle
**ThrS**	threonyl-tRNA synthetase	25.05	1.52	18.46	2.86	1.36	0.03	0.44	Protein synthesis:tRN	TIGR00418 thr	tRNA aminoacylation
**YdgA**	hypothetical protein	5.31	1.15	3.92	0.47	1.35	0.06	0.44	N/A	N/A	N/A
**AcnB**	aconitate hydratase	48.57	1.32	36.12	0.47	1.34	0.00	0.43	Energy metabolism:T	TIGR00117 aco	TCA cycle
**FabG**	3-oxoacyl-[acyl-carrier-protein] reductase	18.45	2.41	13.74	2.26	1.34	0.04	0.43	Fatty acid and phosp	TIGR01830 3-o	Biosynthesis
**PntA**	NAD(P) transhydrogenase subunit alpha	29.37	7.78	21.87	3.15	1.34	0.09	0.43	Energy metabolism:E	TIGR00561 NA	Electron transport
**Tpx**	thiol peroxidase	26.05	2.78	19.62	1.96	1.33	0.02	0.41	N/A	N/A	N/A
**SerA**	D-3-phosphoglycerate dehydrogenase	40.45	3.11	30.52	0.92	1.33	0.01	0.41	N/A	N/A	N/A
**YadR**	hypothetical protein	8.13	2.45	6.15	1.21	1.32	0.13	0.40	Biosynthesis of cofac	TIGR00049 iro	Other
**SodB**	superoxide dismutase, Fe	19.25	3.05	14.56	2.38	1.32	0.05	0.40	N/A	N/A	N/A
**Asd**	aspartate-semialdehyde dehydrogenase	42.93	3.24	32.5	4.05	1.32	0.02	0.40	Amino acid biosynth	TIGR01745 asp	Aspartate family
**PpiD**	peptidyl-prolyl cis-trans isomerase (rotam	18.51	1.96	14.02	3.08	1.32	0.06	0.40	N/A	N/A	N/A
**PdxJ**	pyridoxal phosphate biosynthetic protein	8.26	0.99	6.44	0.47	1.28	0.02	0.36	Biosynthesis of cofac	TIGR00559 pyr	Pyridoxine
**Pnp**	polynucleotide phosphorylase/polyadenyl	32.96	2.14	25.75	2.1	1.28	0.01	0.36	Transcription:Degrad	TIGR03591 pol	Degradation of RNA
**SseA**	putative thiosulfate sulfurtransferase	5.70	1.54	4.48	0.94	1.27	0.12	0.35	N/A	N/A	N/A
**FkpA**	FKBP-type peptidyl-prolyl cis-trans isomer	10.61	2.30	8.4	0.75	1.26	0.05	0.34	N/A	N/A	N/A
**KatG**	catalase/hydroperoxidase HPI(I)	26.73	1.15	21.3	1.99	1.26	0.01	0.33	Cellular processes:De	TIGR00198 cat	Detoxification
**TrxB**	thioredoxin reductase, FAD/NAD(P)-bindin	25.63	4.26	20.46	2.8	1.25	0.05	0.32	Energy metabolism:E	TIGR01292 thio	Electron transport
**HtpG**	heat shock protein 90	23.78	2.24	19.04	1.88	1.25	0.02	0.32	N/A	N/A	N/A
**HlpA**	periplasmic chaperone	11.17	1.61	8.95	1.63	1.25	0.05	0.32	N/A	N/A	N/A
**Mdh**	malate dehydrogenase	76.14	6.03	61.03	7.51	1.25	0.02	0.32	Energy metabolism:T	TIGR01772 ma	TCA cycle
**Hns**	global DNA-binding transcriptional dual re	42.58	5.25	34.15	7.64	1.25	0.07	0.32	N/A	N/A	N/A
**GdhA**	glutamate dehydrogenase	34.40	5.40	27.71	2.21	1.24	0.03	0.31	N/A	N/A	N/A
**AtpD**	F0F1 ATP synthase subunit beta	90.98	1.67	73.37	4.12	1.24	0.00	0.31	Energy metabolism:A	TIGR01039 ATP	ATP-proton motive force interconversion
**InfB**	translation initiation factor IF-2	45.38	10.25	36.97	3.04	1.23	0.06	0.30	Protein synthesis:Tra	TIGR00231 sm	Translation factors
**Ais**	hypothetical protein	8.60	1.66	7.01	0.54	1.23	0.04	0.29	N/A	N/A	N/A

**FbaA**	fructose-bisphosphate aldolase	31.85	5.19	26.04	0.62	1.22	0.03	0.29	Energy metabolism:G	TIGR00167 ket	Glycolysis/gluconeogenesis
**SlyD**	FKBP-type peptidyl prolyl cis-trans isomera	9.93	1.55	8.13	2.18	1.22	0.10	0.29	N/A	N/A	N/A
**HupA**	HU, DNA-binding transcriptional regulator	38.61	11.00	31.66	3.3	1.22	0.09	0.29	N/A	N/A	N/A
**DeoD**	purine nucleoside phosphorylase	9.83	1.61	8.11	2.04	1.21	0.09	0.28	Purines, pyrimidines	TIGR00107 pur	Salvage of nucleosides and nucleotides
**RplA**	50S ribosomal protein L1	27.06	7.69	22.41	1.1	1.21	0.08	0.27	Protein synthesis:Rib	TIGR01169 ribo	Ribosomal proteins
**Rne**	fused ribonucleaseE: endoribonuclease/RN	15.57	2.72	12.9	1.9	1.21	0.05	0.27	Transcription:Degrad	TIGR00757 ribo	Degradation of RNA
**GrxC**	glutaredoxin 3	6.07	3.05	5.04	0.78	1.20	0.28	0.27	Energy metabolism:E	TIGR02181 glu	Electron transport
**TrpB**	tryptophan synthase subunit beta	18.90	1.76	15.7	2.27	1.20	0.03	0.27	Amino acid biosynth	TIGR00263 tryp	Aromatic amino acid family
**Lpd**	dihydrolipoamide dehydrogenase	79.88	19.65	66.4	3.68	1.20	0.06	0.27	N/A	TIGR01350 dih	N/A
**LuxS**	S-ribosylhomocysteinase	8.71	2.64	7.27	1.66	1.20	0.14	0.26	N/A	N/A	N/A
**CysK**	cysteine synthase A, O-acetylserine sulfhy	139.00	6.14	116.49	1.86	1.19	0.00	0.25	Amino acid biosynth	TIGR01136 cys	Serine family
**LpcA**	phosphoheptose isomerase	7.34	1.76	6.16	1.3	1.19	0.10	0.25	Cell envelope:Biosyn	TIGR00441 pho	Biosynthesis and degradation of surface polysaccharides and lipopolysaccharides
**AcrA**	multidrug efflux system	10.67	1.46	8.96	0.86	1.19	0.03	0.25	Transport and bindin	TIGR01730 effl	Unknown substrate
**SspA**	stringent starvation protein A	14.60	4.64	12.33	0.63	1.18	0.10	0.24	N/A	N/A	N/A
**YajQ**	orf, hypothetical protein	10.60	2.44	8.96	1.29	1.18	0.07	0.24	N/A	N/A	N/A
**LivJ**	leucine/isoleucine/valine transporter subu	14.22	4.32	12.04	1.7	1.18	0.11	0.24	N/A	N/A	N/A
**YcgK**	hypothetical protein	8.26	0.99	7.01	0.54	1.18	0.02	0.24	N/A	N/A	N/A
**GpmA**	phosphoglyceromutase	42.04	2.25	35.88	4.68	1.17	0.02	0.23	N/A	TIGR01258 pho	N/A
**TyrB**	tyrosine aminotransferase, tyrosine-repres	17.71	1.67	15.13	1.01	1.17	0.01	0.23	N/A	N/A	N/A
**ProA**	gamma-glutamyl phosphate reductase	8.19	1.56	7.01	1.34	1.17	0.07	0.22	Amino acid biosynth	TIGR00407 glu	Glutamate family
**SerC**	phosphoserine aminotransferase	39.77	3.62	34.18	6.31	1.16	0.04	0.22	Amino acid biosynth	TIGR01364 pho	Serine family
**PurC**	phosphoribosylaminoimidazole-succinocar	13.98	0.68	12.05	1.38	1.16	0.02	0.21	Purines, pyrimidines	TIGR00081 pho	Purine ribonucleotide biosynthesis
**Fdx**	[2Fe-2S] ferredoxin	6.14	0.84	5.31	1.21	1.16	0.07	0.21	Biosynthesis of cofac	TIGR02007 fer	Other
**LysC**	aspartate kinase III	35.91	8.12	31.1	1.19	1.15	0.05	0.21	Amino acid biosynth	TIGR00656 asp	Aspartate family
**UdhA**	putative oxidoreductase	11.93	2.37	10.35	1.71	1.15	0.07	0.20	N/A	N/A	N/A
**MetK**	S-adenosylmethionine synthetase	26.71	5.43	23.27	3.65	1.15	0.07	0.20	Central intermediary	TIGR01034 me	Other
**Pps**	phosphoenolpyruvate synthase	23.44	2.46	20.43	1.88	1.15	0.02	0.20	Energy metabolism:G	TIGR01418 pho	Glycolysis/gluconeogenesis
**YidC**	inner membrane protein translocase comp	8.67	0.59	7.56	0.76	1.15	0.01	0.20	Protein fate:Protein	TIGR03592 me	Protein and peptide secretion and trafficking
**PykF**	pyruvate kinase	21.50	2.17	18.77	1.44	1.15	0.02	0.20	Energy metabolism:G	TIGR01064 pyr	Glycolysis/gluconeogenesis
**AckA**	acetate kinase	15.90	5.08	14	1.29	1.14	0.11	0.18	Energy metabolism:F	TIGR00016 ace	Fermentation
**PflB**	pyruvate formate lyase I	17.75	1.26	15.67	1.95	1.13	0.02	0.18	Energy metabolism:F	TIGR01255 for	Fermentation
**TrpD**	bifunctional indole-3-glycerol-phosphate s	18.10	2.01	15.98	3.14	1.13	0.05	0.18	Amino acid biosynth	TIGR00566 glu	Aromatic amino acid family
**Tdh**	L-threonine 3-dehydrogenase	5.40	1.09	4.77	1.8	1.13	0.18	0.18	Energy metabolism:A	TIGR00692 L-th	Amino acids and amines
**FtsH**	protease, ATP-dependent zinc-metallo	27.34	4.01	24.38	3.81	1.12	0.05	0.17	Protein fate:Degrada	TIGR01241 ATP	Degradation of proteins, peptides, and glycopeptides
**PurR**	DNA-binding transcriptional repressor, hyp	5.66	2.60	5.06	1.73	1.12	0.33	0.16	N/A	N/A	N/A
**HslU**	ATP-dependent protease ATP-binding subu	15.34	2.85	13.72	0.89	1.12	0.04	0.16	Protein fate:Protein	TIGR00390 ATP	Protein folding and stabilization
**AsnS**	asparaginyl-tRNA synthetase	26.86	2.41	24.08	0.68	1.12	0.01	0.16	Protein synthesis:tRN	TIGR00457 asp	tRNA aminoacylation
**AtpA**	F0F1 ATP synthase subunit alpha	89.32	10.60	80.63	6.3	1.11	0.02	0.15	Energy metabolism:A	TIGR00962 ATP	ATP-proton motive force interconversion
**FabH**	3-oxoacyl-(acyl carrier protein) synthase	9.93	2.37	8.97	1.38	1.11	0.08	0.15	Fatty acid and phosp	TIGR00747 3-o	Biosynthesis
**GlmS**	D-fructose-6-phosphate amidotransferase	17.65	1.98	15.96	0.67	1.11	0.01	0.15	Central intermediary	TIGR01135 glu	Amino sugars
**HscA**	chaperone protein HscA	10.81	3.66	9.8	1.26	1.10	0.13	0.14	Protein fate:Protein	TIGR01991 Fe-	Protein folding and stabilization
**RpsB**	30S ribosomal protein S2	24.62	3.03	22.39	2.14	1.10	0.02	0.14	Protein synthesis:Rib	TIGR01011 ribo	Ribosomal proteins
**YdhD**	hypothetical protein	8.32	2.02	7.57	0.91	1.10	0.07	0.14	Energy metabolism:E	TIGR00365 mo	Electron transport
**MaeB**	malic enzyme	8.30	2.16	7.57	1.49	1.10	0.11	0.13	N/A	N/A	N/A
**Crr**	glucose-specific PTS system enzyme IIA co	28.82	0.63	26.3	3.19	1.10	0.02	0.13	Signal transduction:P	TIGR00830 PTS	PTS
**TrpC**	N-(5-phosphoribosyl)anthranilate isomera	16.25	4.92	14.85	0.66	1.09	0.09	0.13	N/A	N/A	N/A
**RpsI**	30S ribosomal protein S9	6.10	1.84	5.61	1.79	1.09	0.19	0.12	N/A	N/A	N/A
**Gnd**	6-phosphogluconate dehydrogenase	34.09	2.83	31.35	1.56	1.09	0.01	0.12	Energy metabolism:P	TIGR00873 6-p	Pentose phosphate pathway
**TrpA**	tryptophan synthase subunit alpha	27.42	3.77	25.24	6.24	1.09	0.08	0.12	Amino acid biosynth	TIGR00262 tryp	Aromatic amino acid family
**Pgk**	phosphoglycerate kinase	79.94	2.24	73.69	4.36	1.08	0.00	0.12	N/A	N/A	N/A
**Dps**	DNA protection during starvation conditio	6.99	0.34	6.45	1.03	1.08	0.03	0.12	N/A	N/A	N/A
**MetQ**	DL-methionine transporter subunit	34.90	7.79	32.51	2.31	1.07	0.05	0.10	Cell envelope:Other	TIGR00363 lipo	Other
**ArcA**	DNA-binding response regulator in two-co	10.79	2.18	10.07	0.73	1.07	0.05	0.10	N/A	N/A	N/A
**YfbG**	hypothetical protein	15.27	1.13	14.28	3.04	1.07	0.05	0.10	N/A	N/A	N/A
**SohB**	predicted inner membrane peptidase	6.58	0.44	6.16	0.54	1.07	0.01	0.10	N/A	N/A	N/A
**SecD**	protein export protein SecD	12.28	3.33	11.49	1.82	1.07	0.10	0.10	Protein fate:Protein	TIGR00916 pro	Protein and peptide secretion and trafficking
**RpsE**	30S ribosomal protein S5	18.83	7.92	17.64	0.9	1.07	0.18	0.09	Protein synthesis:Rib	TIGR01021 ribo	Ribosomal proteins
**UspG**	universal stress protein UP12	12.28	1.67	11.5	4.7	1.07	0.19	0.09	N/A	N/A	N/A
**CysJ**	sulfite reductase, alpha subunit, flavoprot	36.48	6.51	34.17	3.31	1.07	0.04	0.09	N/A	TIGR01931 sul	N/A
**HisF**	imidazole glycerol phosphate synthase sub	14.60	4.64	13.72	1.31	1.06	0.11	0.09	Amino acid biosynth	TIGR00735 imi	Histidine family
**PepQ**	proline dipeptidase	12.20	3.67	11.49	2.49	1.06	0.14	0.09	N/A	N/A	N/A
**TufA**	protein chain elongation factor EF-Tu (dup	448.63	29.39	422.86	4.91	1.06	0.00	0.09	Protein synthesis:Tra	TIGR00231 sm	Translation factors

**RibE**	riboflavin synthase subunit beta	15.64	0.87	14.84	1.18	1.05	0.01	0.08	Biosynthesis of cofac	TIGR00114 6,7	Riboflavin, FMN, and FAD
**GalU**	glucose-1-phosphate uridylyltransferase	7.38	0.75	7	0.52	1.05	0.02	0.08	Cell envelope:Biosyn	TIGR01099 UT	Biosynthesis and degradation of surface polysaccharides and lipopolysaccharides
**Pta**	phosphate acetyltransferase	37.66	4.98	35.86	2.62	1.05	0.02	0.07	Energy metabolism:F	TIGR00651 pho	Fermentation
**NudE**	ADP-ribose diphosphatase	7.04	1.11	6.71	1.42	1.05	0.07	0.07	N/A	N/A	N/A
**MetE**	5-methyltetrahydropteroyltriglutamate-- h	98.64	13.04	94.14	5.25	1.05	0.02	0.07	Amino acid biosynth	TIGR01371 5-n	Aspartate family
**RplO**	50S ribosomal protein L15	16.71	3.90	15.97	1.73	1.05	0.07	0.07	Protein synthesis:Rib	TIGR01071 ribo	Ribosomal proteins
**RpsG**	30S ribosomal protein S7	30.95	2.05	29.71	2.76	1.04	0.01	0.06	Protein synthesis:Rib	TIGR01029 ribo	Ribosomal proteins
**PanB**	3-methyl-2-oxobutanoate hydroxymethylt	4.96	0.34	4.76	0.47	1.04	0.01	0.06	Biosynthesis of cofac	TIGR00222 3-n	Pantothenate and coenzyme A
**YdgH**	hypothetical protein	7.84	0.79	7.56	0.09	1.04	0.01	0.05	N/A	N/A	N/A
**DapD**	2,3,4,5-tetrahydropyridine-2-carboxylate N	32.17	11.31	31.07	4.26	1.04	0.14	0.05	Amino acid biosynth	TIGR00965 2,3	Aspartate family
**ArgD**	bifunctional acetylornithine aminotransfe	8.71	1.72	8.41	2.28	1.04	0.11	0.05	Energy metabolism:A	TIGR00707 ace	Amino acids and amines
**MoaB**	molybdopterin biosynthesis protein B	5.21	6.13	5.04	0.89	1.03	1.41	0.05	Biosynthesis of cofac	TIGR00177 mo	Molybdopterin
**YfiO**	predicted lipoprotein	5.79	0.88	5.6	2.11	1.03	0.17	0.05	Protein fate:Protein	TIGR03302 out	Protein and peptide secretion and trafficking
**AccD**	acetyl-CoA carboxylase subunit beta	12.72	1.09	12.33	1.43	1.03	0.02	0.04	Fatty acid and phosp	TIGR00515 ace	Biosynthesis
**GuaA**	bifunctional GMP synthase/glutamine ami	11.83	2.35	11.48	0.62	1.03	0.04	0.04	Purines, pyrimidines,	TIGR00884 GM	Purine ribonucleotide biosynthesis
**HisS**	histidyl-tRNA synthetase	20.19	1.62	19.59	1.05	1.03	0.01	0.04	Protein synthesis:tRN	TIGR00442 hist	tRNA aminoacylation
**BetB**	betaine aldehyde dehydrogenase, NAD-de	6.60	0.76	6.44	1.23	1.02	0.05	0.04	Cellular processes:Ad	TIGR01804 bet	Adaptations to atypical conditions
**PurH**	bifunctionalphosphoribosylaminoimidazol	19.43	4.07	19.03	3.44	1.02	0.08	0.03	Purines, pyrimidines,	TIGR00355 pho	Purine ribonucleotide biosynthesis
**IhfA**	integration host factor subunit alpha	10.30	0.46	10.09	2.56	1.02	0.07	0.03	DNA metabolism:DN	TIGR00987 inte	DNA replication, recombination, and repair
**SerS**	seryl-tRNA synthetase	19.14	5.06	18.76	0.6	1.02	0.07	0.03	Protein synthesis:tRN	TIGR00414 ser	tRNA aminoacylation
**RpsC**	30S ribosomal protein S3	29.99	2.34	29.4	2.93	1.02	0.02	0.03	Protein synthesis:Rib	TIGR01009 ribo	Ribosomal proteins
**RpmC**	50S ribosomal protein L29	15.42	4.21	15.14	1.85	1.02	0.09	0.03	Protein synthesis:Rib	TIGR00012 ribo	Ribosomal proteins
**CarA**	carbamoyl-phosphate synthase small subu	10.28	1.68	10.1	2.32	1.02	0.08	0.03	Purines, pyrimidines,	TIGR01368 car	Pyrimidine ribonucleotide biosynthesis
**CarB**	carbamoyl-phosphate synthase large subu	36.44	4.90	35.87	3.09	1.02	0.03	0.02	Purines, pyrimidines,	TIGR01369 car	Pyrimidine ribonucleotide biosynthesis
**YfiF**	predicted methyltransferase	5.40	3.11	5.32	0.51	1.02	0.34	0.02	N/A	N/A	N/A
**FusA**	elongation factor EF-2	119.74	5.59	118.44	9.23	1.01	0.01	0.02	Protein synthesis:Tra	TIGR00231 sma	Translation factors
**YidA**	predicted hydrolase	5.10	3.70	5.05	0.9	1.01	0.56	0.02	Unknown function:E	TIGR00099 Cof	Enzymes of unknown specificity
**HisC**	histidinol-phosphate aminotransferase	8.49	3.91	8.41	1.71	1.01	0.25	0.01	Amino acid biosynth	TIGR01141 hist	Histidine family
**VacB**	putative enzyme	8.19	1.56	8.12	1.29	1.01	0.06	0.01	Transcription:Degrad	TIGR00358 Vac	Degradation of RNA
**PurL**	phosphoribosylformylglycinamidine synth	21.37	1.67	21.25	5.23	1.01	0.07	0.01	Purines, pyrimidines,	TIGR01735 pho	Purine ribonucleotide biosynthesis
**DacA**	D-alanyl-D-alanine carboxypeptidase (pen	8.15	2.04	8.12	1.21	1.00	0.09	0.01	N/A	N/A	N/A
**PfkB**	6-phosphofructokinase II; suppressor of pf	7.03	0.94	7	0.95	1.00	0.04	0.01	N/A	TIGR03168 hex	N/A
**ArgB**	acetylglutamate kinase (RefSeq)	5.33	1.77	5.31	2.05	1.00	0.26	0.00	Amino acid biosynth	TIGR00761 ace	Glutamate family
**LeuS**	leucyl-tRNA synthetase	17.12	3.83	17.09	2.96	1.00	0.08	0.00	Protein synthesis:tRN	TIGR00396 leu	tRNA aminoacylation
**LeuC**	isopropylmalate isomerase large subunit	28.88	1.49	28.83	2.66	1.00	0.01	0.00	Amino acid biosynth	TIGR00170 3-is	Pyruvate family
**RpsD**	30S ribosomal protein S4	29.08	6.04	29.12	2.54	1.00	0.05	0.00	Protein synthesis:Rib	TIGR01017 ribo	Ribosomal proteins
**Pfs**	5'-methylthioadenosine/S-adenosylhomoc	5.86	2.26	5.87	0.78	1.00	0.17	0.00	Purines, pyrimidines,	TIGR01704 MT	Salvage of nucleosides and nucleotides
**GlyA**	serine hydroxymethyltransferase	42.12	4.26	42.26	2.98	1.00	0.02	0.00	N/A	N/A	N/A
**NemA**	N-ethylmaleimide reductase, FMN-linked	18.95	1.68	19.04	0.74	1.00	0.01	-0.01	N/A	N/A	N/A
**PheS**	phenylalanyl-tRNA synthetase alpha subu	13.27	2.34	13.45	0.99	0.99	0.04	-0.02	Protein synthesis:tRN	TIGR00468 phe	tRNA aminoacylation
**Eda**	keto-hydroxyglutarate-aldolase/keto-deox	7.73	2.12	7.84	0.92	0.99	0.09	-0.02	Energy metabolism:E	TIGR01182 2-d	Entner-Doudoroff
**MreB**	regulator of ftsI, penicillin binding protein	5.79	2.56	5.88	0.78	0.98	0.21	-0.02	Cell envelope:Biosyn	TIGR00904 cell	Biosynthesis and degradation of murein sacculus and peptidoglycan
**AspS**	aspartyl-tRNA synthetase	37.45	2.85	38.08	0.07	0.98	0.01	-0.02	Protein synthesis:tRN	TIGR00459 asp	tRNA aminoacylation
**AtpH**	F0F1 ATP synthase subunit delta	11.57	1.77	11.77	2.31	0.98	0.06	-0.02	Energy metabolism:A	TIGR01145 ATF	ATP-proton motive force interconversion
**LysS**	lysine tRNA synthetase, constitutive	15.40	3.95	15.68	0.78	0.98	0.07	-0.03	Protein synthesis:tRN	TIGR00499 lysi	tRNA aminoacylation
**RpsT**	30S ribosomal protein S20	8.52	2.80	8.68	0.53	0.98	0.11	-0.03	Protein synthesis:Rib	TIGR00029 ribo	Ribosomal proteins
**OppA**	oligopeptide transporter subunit	31.06	4.79	31.66	2.82	0.98	0.03	-0.03	N/A	N/A	N/A
**NusG**	transcription antitermination protein NusG	11.80	3.32	12.03	1.15	0.98	0.09	-0.03	Transcription:Transc	TIGR00922 tra	Transcription factors
**ArgH**	argininosuccinate lyase	10.71	0.27	10.94	2.61	0.98	0.06	-0.03	Amino acid biosynth	TIGR00838 arg	Glutamate family
**AlaS**	alanyl-tRNA synthetase	28.77	2.68	29.4	4.42	0.98	0.03	-0.03	Protein synthesis:tRN	TIGR00344 ala	tRNA aminoacylation
**RplN**	50S ribosomal protein L14	9.30	3.77	9.53	0.56	0.98	0.17	-0.04	Protein synthesis:Rib	TIGR01067 ribo	Ribosomal proteins
**DnaK**	molecular chaperone DnaK	36.44	5.16	37.55	3.11	0.97	0.03	-0.04	Protein fate:Protein	TIGR02350 cha	Protein folding and stabilization
**PpiB**	peptidyl-prolyl cis-trans isomerase B (rota	8.43	4.42	8.69	1.99	0.97	0.33	-0.04	N/A	N/A	N/A
**RpiA**	ribose-5-phosphate isomerase A	9.50	1.06	9.8	0.46	0.97	0.01	-0.04	Energy metabolism:F	TIGR00021 ribo	Pentose phosphate pathway
**RpsR**	30S ribosomal protein S18	9.45	2.40	9.8	0.46	0.96	0.07	-0.05	Protein synthesis:Rib	TIGR00165 ribo	Ribosomal proteins
**LepA**	GTP-binding protein LepA	5.36	0.73	5.59	1.91	0.96	0.14	-0.06	Unknown function:G	TIGR00231 sma	General
**MurA**	UDP-N-acetylglucosamine 1-carboxyvinylt	6.99	0.34	7.29	1.03	0.96	0.02	-0.06	Cell envelope:Biosyn	TIGR01072 UD	Biosynthesis and degradation of murein sacculus and peptidoglycan
**AccC**	acetyl-CoA carboxylase	9.11	1.34	9.51	1.68	0.96	0.05	-0.06	Fatty acid and phosp	TIGR00514 ace	Biosynthesis
**YchF**	translation-associated GTPase	7.76	1.38	8.11	1.65	0.96	0.07	-0.06	Unknown function:G	TIGR00092 GTI	General
**AhpF**	alkyl hydroperoxide reductase, F52a subu	16.60	3.05	17.36	0.4	0.96	0.03	-0.06	Cellular processes:Ad	TIGR03140 alky	Adaptations to atypical conditions
**Pgi**	glucose-6-phosphate isomerase	12.30	1.47	12.89	2.79	0.95	0.06	-0.07	N/A	N/A	N/A
**HemL**	glutamate-1-semialdehyde aminotransfera	20.19	1.69	21.27	2.41	0.95	0.02	-0.08	Biosynthesis of cofac	TIGR00713 glu	Heme, porphyrin, and cobalamin

**IlvC**	ketol-acid reductoisomerase	52.98	11.17	56.54	3.47	0.94	0.05	-0.09	Amino acid biosynth	TIGR00465 ket	Pyruvate family
**ValS**	valyl-tRNA synthetase	23.11	2.26	24.66	1.54	0.94	0.01	-0.09	Protein synthesis:tR	TIGR00422 val	tRNA aminoacylation
**RpoB**	DNA-directed RNA polymerase subunit be	70.83	8.67	76.16	3.59	0.93	0.02	-0.10	Transcription:DNA-d	TIGR02013 DN	DNA-dependent RNA polymerase
**PfkA**	6-phosphofructokinase	17.14	5.96	18.47	1.47	0.93	0.13	-0.11	Energy metabolism:	TIGR02482 6-p	Glycolysis/gluconeogenesis
**PrsA**	ribose-phosphate pyrophosphokinase	28.01	2.03	30.24	2.1	0.93	0.01	-0.11	Purines, pyrimidines	TIGR01251 rib	Purine ribonucleotide biosynthesis
**b2511**	putative GTP-binding factor	7.77	2.92	8.4	1.4	0.93	0.17	-0.11	Protein synthesis:Ot	TIGR00231 sm	Other
**DegP**	serine endoprotease (protease Do), memb	6.99	0.34	7.56	0.86	0.92	0.02	-0.11	Protein fate:Degrada	TIGR02037 pro	Degradation of proteins, peptides, and glycopeptides
**PepP**	proline aminopeptidase P II	8.26	0.99	8.95	1.64	0.92	0.05	-0.12	N/A	N/A	N/A
**B2294**	orf, hypothetical protein	11.87	1.83	12.86	2.54	0.92	0.06	-0.12	N/A	N/A	N/A
**Tsf**	elongation factor Ts	25.29	4.64	27.43	4	0.92	0.05	-0.12	Protein synthesis:Tra	TIGR00116 tra	Translation factors
**PtsI**	PEP-protein phosphotransferase of PTS sy	56.15	3.74	61.36	3.74	0.92	0.01	-0.13	N/A	TIGR01417 ph	N/A
**GroS**	co-chaperonin GroES	17.12	5.92	18.77	1.19	0.91	0.12	-0.13	N/A	N/A	N/A
**RpsJ**	30S ribosomal protein S10	23.16	5.53	25.5	5.71	0.91	0.11	-0.14	Protein synthesis:Rib	TIGR01049 rib	Ribosomal proteins
**FabF**	3-oxoacyl-(acyl carrier protein) synthase	18.56	2.24	20.45	1.45	0.91	0.02	-0.14	Fatty acid and phosp	TIGR03150 bet	Biosynthesis
**PurD**	phosphoribosylamine--glycine ligase	6.58	0.44	7.27	1.21	0.91	0.03	-0.14	Purines, pyrimidines	TIGR00877 ph	Purine ribonucleotide biosynthesis
**ArgS**	arginyl-tRNA synthetase	7.60	4.25	8.39	1.59	0.91	0.35	-0.14	Protein synthesis:tR	TIGR00456 arg	tRNA aminoacylation
**TyrA**	fused chorismate mutase T/prephenate de	60.39	4.44	66.94	1.28	0.90	0.01	-0.15	Amino acid biosynth	TIGR01799 ch	Aromatic amino acid family
**Frr**	ribosome releasing factor	5.77	1.39	6.43	2.06	0.90	0.16	-0.16	Protein synthesis:Tra	TIGR00496 rib	Translation factors
**ThrA**	bifunctional aspartokinase I/homeserine d	25.50	1.19	28.56	3.35	0.89	0.02	-0.16	Amino acid biosynth	TIGR00657 asp	Aspartate family
**DapB**	dihydrodipicolinate reductase	7.75	2.38	8.69	1.35	0.89	0.12	-0.17	Amino acid biosynth	TIGR00036 dih	Aspartate family
**HisJ**	histidine/lysine/arginine/ornithine transp	9.94	1.80	11.2	0.55	0.89	0.04	-0.17	Transport and bindir	TIGR01096 lysi	Amino acids, peptides and amines
**BipA**	GTP-binding protein	16.80	2.67	19.04	3.49	0.88	0.06	-0.18	Protein synthesis:Tra	TIGR00231 sm	Translation factors
**CysP**	thiosulfate transporter subunit	20.98	1.93	23.78	4.38	0.88	0.04	-0.18	Transport and bindir	TIGR00971 sul	Anions
**RplP**	50S ribosomal protein L16	15.73	2.64	17.92	1.63	0.88	0.04	-0.19	Protein synthesis:Rib	TIGR01164 rib	Ribosomal proteins
**RplJ**	50S ribosomal protein L10	25.46	1.43	29.1	5.53	0.87	0.04	-0.19	N/A	N/A	N/A
**Upp**	uracil phosphoribosyltransferase	8.56	1.92	9.79	0.85	0.87	0.06	-0.19	Purines, pyrimidines	TIGR01091 ura	Salvage of nucleosides and nucleotides
**GlnS**	glutaminyl-tRNA synthetase	7.06	2.21	8.11	1.89	0.87	0.15	-0.20	Protein synthesis:tR	TIGR00440 glu	tRNA aminoacylation
**Tig**	trigger factor	49.35	8.13	56.81	4.9	0.87	0.03	-0.20	Protein fate:Protein	TIGR00115 trig	Protein folding and stabilization
**ClpB**	protein disaggregation chaperone	35.94	3.05	41.42	5.53	0.87	0.03	-0.20	Protein fate:Protein	TIGR03346 AT	Protein folding and stabilization
**RpsA**	30S ribosomal protein S1	45.85	6.72	52.9	7.71	0.87	0.04	-0.21	Protein synthesis:Rib	TIGR00717 rib	Ribosomal proteins
**RpoC**	DNA-directed RNA polymerase subunit be	63.56	5.57	73.37	1.19	0.87	0.01	-0.21	Transcription:DNA-d	TIGR02386 DN	DNA-dependent RNA polymerase
**PurB**	adenylosuccinate lyase	9.71	3.91	11.21	2.17	0.87	0.20	-0.21	Purines, pyrimidines	TIGR00928 ade	Purine ribonucleotide biosynthesis
**IscS**	cysteine desulfurase	20.47	2.75	23.79	2.71	0.86	0.03	-0.22	Protein synthesis:tR	TIGR02006 cys	tRNA and rRNA base modification
**TrpE**	anthranilate synthase component I	9.83	1.61	11.47	1.6	0.86	0.05	-0.22	Amino acid biosynth	TIGR00565 ant	Aromatic amino acid family
**ThrC**	threonine synthase	20.01	4.97	23.52	2.09	0.85	0.07	-0.23	Amino acid biosynth	TIGR00260 thr	Aspartate family
**AdhE**	fused acetaldehyde-CoA dehydrogenase/i	26.36	3.75	31.07	2.68	0.85	0.03	-0.24	N/A	N/A	N/A
**HisG**	ATP phosphoribosyltransferase	7.36	1.71	8.68	2.58	0.85	0.14	-0.24	Amino acid biosynth	TIGR00070 AT	Histidine family
**GshA**	glutamate--cysteine ligase	4.96	0.34	5.88	1.42	0.84	0.06	-0.25	Biosynthesis of cofac	TIGR01434 glu	Glutathione and analogs
**AccA**	acetyl-CoA carboxylase subunit alpha	18.15	2.08	21.58	3.38	0.84	0.04	-0.25	Fatty acid and phosp	TIGR00513 ace	Biosynthesis
**DppA**	dipeptide transporter	22.61	1.64	26.89	0.32	0.84	0.01	-0.25	N/A	N/A	N/A
**RplM**	50S ribosomal protein L13	8.71	1.72	10.37	2.16	0.84	0.08	-0.25	Protein synthesis:Rib	TIGR01066 rib	Ribosomal proteins
**YfbF**	undecaprenyl phosphate-L-Ara4FN transfe	5.40	1.09	6.44	1.29	0.84	0.08	-0.25	N/A	N/A	N/A
**NuoC**	NADH:ubiquinone oxidoreductase, chain C	8.67	0.59	10.36	1.74	0.84	0.03	-0.26	Energy metabolism:	TIGR01961 NA	Electron transport
**PurT**	phosphoribosylglycinamide formyltransfer	5.84	4.17	7.01	1.35	0.83	0.55	-0.26	Purines, pyrimidines	TIGR01142 ph	Purine ribonucleotide biosynthesis
**NusA**	transcription elongation factor NusA	17.64	1.46	21.26	2.7	0.83	0.02	-0.27	Transcription:Transc	TIGR01953 tra	Transcription factors
**RfaE**	fused heptose 7-phosphate kinase/heptos	9.50	1.06	11.47	1.6	0.83	0.03	-0.27	Cell envelope:Biosyn	TIGR00125 cyt	Biosynthesis and degradation of surface polysaccharides and lipopolysaccharides
**GlyS**	glycyl-tRNA synthetase subunit beta	17.12	3.83	20.71	4.84	0.83	0.10	-0.27	Protein synthesis:tR	TIGR00211 gly	tRNA aminoacylation
**GlnA**	glutamine synthetase	9.02	2.78	10.92	1.68	0.83	0.12	-0.28	Amino acid biosynth	TIGR00653 glu	Glutamate family
**YgfZ**	putative global regulator	6.71	2.96	8.13	0.58	0.83	0.20	-0.28	Protein synthesis:tR	TIGR03317 fol	tRNA and rRNA base modification
**DadA**	D-amino acid dehydrogenase small subuni	5.75	0.40	6.99	3.39	0.82	0.24	-0.28	N/A	N/A	N/A
**RplQ**	50S ribosomal protein L17	15.18	2.07	18.47	1.47	0.82	0.02	-0.28	Protein synthesis:Rib	TIGR00059 rib	Ribosomal proteins
**RbfA**	ribosome-binding factor A	5.29	1.54	6.44	1	0.82	0.11	-0.28	Transcription:RNA p	TIGR00082 rib	RNA processing
**RplF**	50S ribosomal protein L6	24.38	3.58	29.69	3.44	0.82	0.03	-0.28	Protein synthesis:Rib	TIGR03654 rib	Ribosomal proteins
**YibN**	predicted rhodanese-related sulfurtransfe	5.71	1.67	7	0.93	0.82	0.10	-0.29	N/A	N/A	N/A
**HisB**	imidazoleglycerolphosphate dehydratase a	4.99	1.52	6.15	1.21	0.81	0.13	-0.30	Amino acid biosynth	TIGR01261 his	Histidine family
**AceE**	pyruvate dehydrogenase subunit E1	77.27	12.78	95.2	2.46	0.81	0.03	-0.30	N/A	TIGR00759 pyr	N/A
**Zwf**	glucose-6-phosphate 1-dehydrogenase	12.22	2.91	15.12	1.36	0.81	0.06	-0.31	Energy metabolism:	TIGR00871 glu	Pentose phosphate pathway
**IleS**	isoleucyl-tRNA synthetase	20.73	6.08	25.75	4.02	0.80	0.11	-0.31	Protein synthesis:tR	TIGR00392 iso	tRNA aminoacylation
**RpsP**	30S ribosomal protein S16	8.95	3.46	11.21	1.09	0.80	0.16	-0.32	Protein synthesis:Rib	TIGR00002 rib	Ribosomal proteins
**GyrB**	DNA gyrase subunit B	8.71	1.72	10.93	1.54	0.80	0.06	-0.33	DNA metabolism:DN	TIGR01059 DN	DNA replication, recombination, and repair
**AtpF**	F0F1 ATP synthase subunit B	11.15	0.76	14.01	1.43	0.80	0.02	-0.33	Energy metabolism:	TIGR01144 AT	ATP-proton motive force interconversion
**YebL**	putative adhesin	23.96	2.38	30.26	1.8	0.79	0.01	-0.34	N/A	N/A	N/A

**GroEL**	chaperonin GroEL	101.82	3.08	128.79	4.86	0.79	0.00	-0.34	Protein fate:Protein	TIGR02348 cha	Protein folding and stabilization
**Rho**	transcription termination factor Rho	39.16	10.14	49.59	2.17	0.79	0.07	-0.34	Transcription:Transc	TIGR00767 tra	Transcription factors
**PepD**	aminoacyl-histidine dipeptidase (peptidas	7.73	2.12	9.79	2.03	0.79	0.12	-0.34	N/A	TIGR01893 am	N/A
**GlnH**	glutamine ABC transporter periplasmic pro	7.49	1.75	9.52	0.91	0.79	0.06	-0.35	N/A	N/A	N/A
**SolA**	N-methyltryptophan oxidase, FAD-binding	5.29	1.54	6.73	0.9	0.79	0.10	-0.35	N/A	N/A	N/A
**TrxA**	thioredoxin 1	10.28	1.68	13.17	1.42	0.78	0.04	-0.36	Energy metabolism:E	TIGR01068 thi	Electron transport
**Ppc**	phosphoenolpyruvate carboxylase	36.92	3.34	47.33	3.81	0.78	0.01	-0.36	N/A	N/A	N/A
**RpoA**	DNA-directed RNA polymerase subunit alp	39.25	6.35	50.41	7.74	0.78	0.05	-0.36	Transcription:DNA-d	TIGR02027 DN	DNA-dependent RNA polymerase
**AceF**	dihydrolipoamide acetyltransferase	37.16	2.52	47.88	3.42	0.78	0.01	-0.37	Energy metabolism:P	TIGR01348 dih	Pyruvate dehydrogenase
**YeeZ**	predicted epimerase, with NAD(P)-binding	7.80	1.71	10.09	2.26	0.77	0.10	-0.37	N/A	N/A	N/A
**PyrG**	CTP synthetase	12.35	0.96	15.97	0.19	0.77	0.01	-0.37	Purines, pyrimidines	TIGR00337 CTP	Pyrimidine ribonucleotide biosynthesis
**PanC**	pantoate--beta-alanine ligase	6.49	2.20	8.4	0.76	0.77	0.12	-0.37	Biosynthesis of cofac	TIGR00018 par	Pantothenate and coenzyme A
**PykA**	pyruvate kinase	19.91	3.52	25.77	4.05	0.77	0.06	-0.37	Energy metabolism:G	TIGR01064 pyr	Glycolysis/gluconeogenesis
**YjjK**	fused predicted transporter subunits of AB	10.32	0.89	13.44	1.68	0.77	0.02	-0.38	N/A	TIGR03719 ATP	N/A
**HupB**	HU, DNA-binding transcriptional regulator	6.88	4.21	8.96	1.26	0.77	0.39	-0.38	N/A	N/A	N/A
**PurA**	adenylosuccinate synthetase	24.46	6.12	31.92	1.96	0.77	0.07	-0.38	Purines, pyrimidines	TIGR00184 ade	Purine ribonucleotide biosynthesis
**GltA**	citrate synthase	49.59	6.37	64.72	2.95	0.77	0.02	-0.38	Energy metabolism:T	TIGR01798 citr	TCA cycle
**LeuB**	3-isopropylmalate dehydrogenase	32.57	2.02	42.87	3.76	0.76	0.01	-0.40	Amino acid biosynth	TIGR00169 3-is	Pyruvate family
**YciK**	short chain dehydrogenase	6.14	0.84	8.12	2.11	0.76	0.09	-0.40	N/A	N/A	N/A
**AroG**	3-deoxy-D-arabino-heptulosonate-7-phosp	28.53	7.56	37.81	2.64	0.75	0.08	-0.41	Amino acid biosynth	TIGR00034 3-d	Aromatic amino acid family
**Tas**	predicted oxidoreductase, NADP(H)-depen	5.25	2.29	7.01	2.15	0.75	0.28	-0.42	N/A	N/A	N/A
**PheT**	phenylalanyl-tRNA synthetase beta subun	50.42	6.42	67.54	11.1	0.75	0.04	-0.42	Protein synthesis:tRN	TIGR00472 phe	tRNA aminoacylation
**NrdA**	ribonucleotide-diphosphate reductase alp	8.74	2.57	11.77	0.95	0.74	0.09	-0.43	Purines, pyrimidines	TIGR02506 rib	2'-Deoxyribonucleotide metabolism
**LeuD**	isopropylmalate isomerase small subunit	13.18	0.33	17.93	0.6	0.74	0.00	-0.44	Amino acid biosynth	TIGR00171 3-is	Pyruvate family
**RpsK**	30S ribosomal protein S11	13.79	5.05	18.77	2.2	0.73	0.15	-0.44	Protein synthesis:Rib	TIGR03632 30S	Ribosomal proteins
**Adk**	adenylate kinase	12.33	3.07	17.11	2.76	0.72	0.09	-0.47	Purines, pyrimidines	TIGR01351 ade	Nucleotide and nucleoside interconversions
**Apt**	adenine phosphoribosyltransferase	7.36	2.25	10.34	3.35	0.71	0.20	-0.49	Purines, pyrimidines	TIGR01090 ade	Salvage of nucleosides and nucleotides
**GlmM**	phosphoglucosamine mutase	6.56	1.74	9.25	1.71	0.71	0.10	-0.50	Central intermediary	TIGR01455 pho	Amino sugars
**MetG**	methionyl-tRNA synthetase	15.99	1.42	22.69	2.51	0.70	0.02	-0.50	Protein synthesis:tRN	TIGR00398 me	tRNA aminoacylation
**HisA**	N-(5'-phospho-L-ribosyl-formimino)-5-ami	9.49	1.43	13.71	1.12	0.69	0.03	-0.53	Amino acid biosynth	TIGR00007 1-(5	Histidine family
**ClpP**	ATP-dependent Clp protease proteolytic su	5.81	1.03	8.41	1.71	0.69	0.07	-0.53	Protein fate:Degrada	TIGR00493 ATP	Degradation of proteins, peptides, and glycopeptides
**AroF**	3-deoxy-D-arabino-heptulosonate-7-phosp	104.36	23.91	151.44	14.01	0.69	0.06	-0.54	Amino acid biosynth	TIGR00034 3-d	Aromatic amino acid family
**RplI**	50S ribosomal protein L9	31.89	5.92	47.33	5.62	0.67	0.05	-0.57	Protein synthesis:Rib	TIGR00158 rib	Ribosomal proteins
**GutQ**	orf, hypothetical protein	8.63	0.99	12.88	0.33	0.67	0.01	-0.58	Energy metabolism:S	TIGR00393 sug	Sugars
**NarL**	DNA-binding response regulator in two-co	8.23	0.29	12.31	2.01	0.67	0.03	-0.58	N/A	N/A	N/A
**YcdW**	putative dehydrogenase	5.80	2.95	8.69	1.35	0.67	0.28	-0.58	N/A	N/A	N/A
**Eno**	phosphopyruvate hydratase	66.88	5.09	100.49	5.76	0.67	0.01	-0.59	Energy metabolism:G	TIGR01060 pho	Glycolysis/gluconeogenesis
**PotD**	spermidine/putrescine ABC transporter pe	6.14	0.84	9.24	0.74	0.66	0.02	-0.59	N/A	N/A	N/A
**GuaB**	inositol-5-monophosphate dehydrogenase	11.90	2.10	17.94	2.3	0.66	0.05	-0.59	Purines, pyrimidines	TIGR01302 ino	Purine ribonucleotide biosynthesis
**IlvB**	acetolactate synthase large subunit	9.99	2.64	15.11	2.1	0.66	0.09	-0.60	Amino acid biosynth	TIGR00118 ace	Pyruvate family
**ProS**	prolyl-tRNA synthetase	20.22	2.83	30.79	2.23	0.66	0.02	-0.61	Protein synthesis:tRN	TIGR00409 pro	tRNA aminoacylation
**AcnA**	aconitate hydratase	11.04	1.75	16.82	1.65	0.66	0.03	-0.61	Energy metabolism:T	TIGR01341 aco	TCA cycle
**RplB**	50S ribosomal protein L2	27.62	1.87	42.31	2.24	0.65	0.01	-0.62	Protein synthesis:Rib	TIGR01171 rib	Ribosomal proteins
**RplE**	50S ribosomal protein L5	25.48	1.47	40.04	3.14	0.64	0.01	-0.65	N/A	N/A	N/A
**StpA**	DNA binding protein, nucleoid-associated	8.56	1.92	13.46	2.36	0.64	0.08	-0.65	N/A	N/A	N/A
**IlvE**	branched-chain amino acid aminotransfer	11.93	0.91	18.77	1.48	0.64	0.01	-0.65	Amino acid biosynth	TIGR01122 bra	Pyruvate family
**RfaD**	ADP-L-glycero-D-mannoheptose-6-epimer	9.56	2.10	15.11	1.51	0.63	0.06	-0.66	Cell envelope:Biosyn	TIGR02197 AD	Biosynthesis and degradation of surface polysaccharides and lipopolysaccharides
**FabZ**	(3R)-hydroxymyristoyl ACP dehydratase	6.51	2.32	10.37	0.54	0.63	0.13	-0.67	Fatty acid and phosp	TIGR01750 bet	Biosynthesis
**YliJ**	putative transferase	8.40	6.13	13.45	2.32	0.62	0.56	-0.68	N/A	N/A	N/A
**FabB**	3-oxoacyl-(acyl carrier protein) synthase	18.29	5.51	29.39	2.72	0.62	0.10	-0.68	N/A	N/A	N/A
**MoeA**	molybdopterin biosynthesis protein	5.31	1.15	8.68	1.72	0.61	0.09	-0.71	N/A	TIGR00177 mo	N/A
**ArgG**	argininosuccinate synthase	12.61	3.51	20.73	1.09	0.61	0.08	-0.72	Amino acid biosynth	TIGR00032 arg	Glutamate family
**IlvD**	dihydroxy-acid dehydratase	40.47	2.99	66.66	2.25	0.61	0.01	-0.72	Amino acid biosynth	TIGR00110 dih	Pyruvate family
**RplY**	50S ribosomal protein L25	15.62	1.31	25.74	3.95	0.61	0.03	-0.72	N/A	N/A	N/A
**FtsZ**	cell division protein FtsZ	14.72	2.39	24.38	1.91	0.60	0.03	-0.73	Cellular processes:Ce	TIGR00065 cell	Cell division
**YbeZ**	predicted protein with nucleoside triphos	7.47	2.59	12.6	1.68	0.59	0.14	-0.75	N/A	N/A	N/A
**Usg**	hypothetical protein	12.61	3.51	21.29	2.21	0.59	0.09	-0.76	N/A	N/A	N/A
**KdsA**	2-dehydro-3-deoxyphosphooctonate aldol	9.76	2.72	16.54	1.45	0.59	0.09	-0.76	Cell envelope:Biosyn	TIGR01362 3-d	Biosynthesis and degradation of surface polysaccharides and lipopolysaccharides
**GltB**	glutamate synthase, large subunit	35.03	1.13	59.94	1.09	0.58	0.00	-0.77	N/A	N/A	N/A
**RplC**	50S ribosomal protein L3	15.01	4.37	25.75	2.1	0.58	0.09	-0.78	N/A	TIGR03625 50S	N/A
**RplD**	50S ribosomal protein L4	15.27	2.69	26.88	0.62	0.57	0.03	-0.82	Protein synthesis:Rib	TIGR03953 50S	Ribosomal proteins
**NadE**	NAD synthetase	6.99	0.34	12.32	1.19	0.57	0.01	-0.82	Biosynthesis of cofac	TIGR00552 NA	Pyridine nucleotides

**YbiB**	hypothetical protein	5.36	0.73	9.52	0.91	0.56	0.03	-0.83	N/A	N/A	N/A
**GyrA**	DNA gyrase subunit A	6.92	2.89	12.33	1.83	0.56	0.20	-0.83	DNA metabolism:DN	TIGR01063 DN	DNA replication, recombination, and repair
**LeuA**	2-isopropylmalate synthase	10.65	1.20	19.32	4.43	0.55	0.07	-0.86	Amino acid biosynth	TIGR00973 2-is	Pyruvate family
**FolE**	GTP cyclohydrolase I	14.33	2.08	26.04	3.79	0.55	0.04	-0.86	Biosynthesis of cofac	TIGR00063 GTI	Folic acid
**Ndk**	nucleoside diphosphate kinase	11.37	3.59	20.73	0.46	0.55	0.10	-0.87	N/A	N/A	N/A
**NuoG**	NADH:ubiquinone oxidoreductase, chain G	9.56	2.10	17.93	1.17	0.53	0.05	-0.91	Energy metabolism:E	TIGR01973 NA	Electron transport
**MetF**	5,10-methylenetetrahydrofolate reductase	7.01	1.40	13.17	1.42	0.53	0.05	-0.91	Amino acid biosynth	TIGR00676 5,1	Aspartate family
**RpsM**	30S ribosomal protein S13	5.77	1.83	11.22	2.2	0.51	0.14	-0.96	Protein synthesis:Rib	TIGR03631 30S	Ribosomal proteins
**SecA**	translocase	11.06	1.54	21.58	1.96	0.51	0.03	-0.96	Protein fate:Protein	TIGR00963 pre	Protein and peptide secretion and trafficking
**RpoD**	RNA polymerase sigma factor	7.03	0.94	13.74	2.57	0.51	0.05	-0.97	Transcription:Transc	TIGR02393 RN	Transcription factors
**GshB**	glutathione synthetase	7.01	1.40	13.71	2.8	0.51	0.08	-0.97	Biosynthesis of cofac	TIGR01380 glu	Glutathione and analogs
**PepN**	aminopeptidase N	6.99	0.34	13.98	2.4	0.50	0.03	-1.00	Protein fate:Degrada	TIGR02414 am	Degradation of proteins, peptides, and glycopeptides
**GltX**	glutamyl-tRNA synthetase	6.29	2.62	12.62	1.82	0.50	0.19	-1.01	Protein synthesis:tRI	TIGR00464 glu	tRNA aminoacylation
**GapA**	glyceraldehyde-3-phosphate dehydrogena	18.47	1.26	37.55	3.37	0.49	0.01	-1.02	Energy metabolism:G	TIGR01534 gly	Glycolysis/gluconeogenesis
**RplV**	50S ribosomal protein L22	13.05	3.38	27.45	1.32	0.48	0.07	-1.07	Protein synthesis:Rib	TIGR01044 ribo	Ribosomal proteins
**RplS**	50S ribosomal protein L19	5.45	2.30	11.77	2.31	0.46	0.22	-1.11	Protein synthesis:Rib	TIGR01024 ribo	Ribosomal proteins
**GltD**	glutamate synthase, 4Fe-4S protein, small	5.44	2.26	12.89	2.05	0.42	0.20	-1.25	N/A	TIGR01318 glu	N/A
**TrpS**	tryptophanyl-tRNA synthetase	6.14	0.84	16.52	1.83	0.37	0.03	-1.43	Protein synthesis:tRI	TIGR00233 tryp	tRNA aminoacylation
**IlvA**	threonine dehydratase	6.29	2.62	18.21	0.7	0.35	0.18	-1.53	Amino acid biosynth	TIGR01124 thr	Pyruvate family
