# Supplementary material for: Manipulation of the carbon storage regulator system for metabolite remodeling and biofuel production in Escherichia coli
Source: Microb Cell Fact. 2012 Jun 13;11:79. doi: 10.1186/1475-2859-11-79 (PMC3460784; doi:10.1186/1475-2859-11-79)
Supplement: Additional file 6 — Table S3. Metabolites impacted by CsrB overexpression. [file 1475-2859-11-79-S6.pdf]

Table S5. Whole-cell proteomics analysis

Protein ID	Identified Proteins (894)	Accession #	Experimental			Control		
			500-1 (F007428)	500-2 (F007429)	500-3 (F007424)	0-1 (F007521_1)	0-2 (F007518_1)	0-3 (F007519_1)
AccA	acetyl-CoA carboxylase subunit alpha (NCBI) [Escherichia coli K12]	VIMSS14331	15.88	19.95	18.63	20.27	25.42	19.06
AccB	acetyl-CoA carboxylase (NCBI) [Escherichia coli K12]	VIMSS17325	2.44	1.33	1.16	1.69	2.54	1.66
AccC	acetyl-CoA carboxylase (NCBI) [Escherichia coli K12]	VIMSS17326	8.55	10.64	8.15	7.6	10.17	10.77
AccD	acetyl-CoA carboxylase subunit beta (NCBI) [Escherichia coli K12]	VIMSS16423	12.22	11.97	13.97	12.67	13.56	10.77
AceA	isocitrate lyase (NCBI) [Escherichia coli K12]	VIMSS18043	0	1.33	0	2.53	0	0.83
AceE	pyruvate dehydrogenase subunit E1 (NCBI) [Escherichia coli K12]	VIMSS14260	84.3	62.52	84.99	92.9	94.91	97.79
AceF	dihydrolipoamide acetyltransferase (NCBI) [Escherichia coli K12]	VIMSS14261	36.65	39.9	34.93	50.67	44.07	48.9
AckA	acetate kinase (NCBI) [Escherichia coli K12]	VIMSS16403	20.77	10.64	16.3	12.67	15.25	14.09
AcnA	aconitate hydratase (NCBI) [Escherichia coli K12]	VIMSS15396	11	9.31	12.81	17.74	17.8	14.92
AcnB	aconitate hydratase (NCBI) [Escherichia coli K12]	VIMSS14264	50.09	47.89	47.73	36.31	35.59	36.47
AcpP	acyl carrier protein (NCBI) [Escherichia coli K12]	VIMSS15216	4.89	2.66	5.82	3.38	5.93	5.8
AcrA	multidrug efflux system (NCBI) [Escherichia coli K12]	VIMSS14600	12.22	9.31	10.48	8.45	8.47	9.95
AcrB	multidrug efflux system protein (NCBI) [Escherichia coli K12]	VIMSS14599	2.44	3.99	4.66	4.22	3.39	4.14
Add	adenosine deaminase (NCBI) [Escherichia coli K12]	VIMSS15744	3.67	1.33	0	3.38	2.54	6.63
AdhE	fused acetaldehyde-CoA dehydrogenase/iron-dependent alcohol dehydrogenase/pyruvate-formate lyase deactivase (NCBI) [Escherichia coli K12]	VIMSS15361	30.54	25.27	23.28	28.71	30.51	33.98
Adk	adenylate kinase (NCBI) [Escherichia coli K12]	VIMSS14611	15.88	10.64	10.48	17.74	19.49	14.09
AhpC	alkyl hydroperoxide reductase, C22 subunit (NCBI) [Escherichia coli K12]	VIMSS14742	47.65	57.2	51.22	17.74	11.86	23.21
AhpF	alkyl hydroperoxide reductase, F52a subunit; detoxification of hydroperoxides (VIMSS) [Escherichia coli K12]	VIMSS14743	15.88	19.95	13.97	17.74	16.95	17.4
Ais	hypothetical protein (NCBI) [Escherichia coli K12]	VIMSS16359	7.33	7.98	10.48	6.76	7.63	6.63
AlaS	alanyl-tRNA synthetase (NCBI) [Escherichia coli K12]	VIMSS16781	31.77	26.6	27.94	24.49	33.05	30.66
AldA	aldehyde dehydrogenase A, NAD-linked (NCBI) [Escherichia coli K12]	VIMSS15537	0	0	0	0.84	0.85	1.66
AllR	DNA-binding transcriptional repressor (NCBI) [Escherichia coli K12]	VIMSS14643	7.33	3.99	5.82	3.38	2.54	2.49
Alr	alanine racemase (NCBI) [Escherichia coli K12]	VIMSS18081	0	0	1.16	0.84	1.69	1.66
Amn	AMP nucleosidase (NCBI) [Escherichia coli K12]	VIMSS16093	3.67	3.99	3.49	1.69	3.39	3.32
AmpE	predicted inner membrane protein (NCBI) [Escherichia coli K12]	VIMSS14257	0	0	0	0	0	1.66
Apt	adenine phosphoribosyltransferase (NCBI) [Escherichia coli K12]	VIMSS14606	9.77	5.32	6.99	9.29	7.63	14.09
ArcA	DNA-binding response regulator in two-component regulatory system with ArcB or CpxA (NCBI) [Escherichia coli K12]	VIMSS18422	9.77	13.3	9.31	10.13	9.32	10.77
ArcB	hybrid sensory histidine kinase in two-component regulatory system with ArcA (NCBI) [Escherichia coli K12]	VIMSS1936962	0	0	1.16	1.69	0	0.83
ArgB	acetylglutamate kinase (VIMSS) [Escherichia coli K12]	VIMSS17998	7.33	3.99	4.66	3.38	5.08	7.46
ArgD	bifunctional acetylornithine aminotransferase/ succinyldiaminopimelate aminotransferase (NCBI) [Escherichia coli K12]	VIMSS17422	7.33	10.64	8.15	10.98	7.63	6.63
ArgE	acetylornithine deacetylase (NCBI) [Escherichia coli K12]	VIMSS17996	0	1.33	0	9.29	7.63	2.49
ArgG	argininosuccinate synthase (NCBI) [Escherichia coli K12]	VIMSS17244	12.22	9.31	16.3	21.96	20.34	19.89
ArgH	argininosuccinate lyase (NCBI) [Escherichia coli K12]	VIMSS17999	11	10.64	10.48	13.51	11.02	8.29
ArgI	ornithine carbamoyltransferase 1 (NCBI) [Escherichia coli K12]	VIMSS18279	2.44	1.33	1.16	1.69	2.54	3.32
ArgP	chromosome replication initiation inhibitor protein (NCBI) [Escherichia coli K12]	VIMSS16995	1.22	0	1.16	3.38	2.54	4.97
ArgR	arginine repressor (NCBI) [Escherichia coli K12]	VIMSS17308	1.22	2.66	0	0.84	0	0
ArgS	arginyl-tRNA synthetase (NCBI) [Escherichia coli K12]	VIMSS15994	7.33	11.97	3.49	8.45	6.78	9.95
ArgT	lysine/arginine/ornithine transporter subunit (NCBI) [Escherichia coli K12]	VIMSS16417	1.22	2.66	2.33	2.53	0.85	3.32
AroA	3-phosphoshikimate 1-carboxyvinyltransferase (NCBI) [Escherichia coli K12]	VIMSS15032	3.67	3.99	2.33	8.45	8.47	8.29
AroB	3-dehydroquinate synthase (NCBI) [Escherichia coli K12]	VIMSS17452	1.22	1.33	4.66	7.6	5.08	5.8
AroC	chorismate synthase (NCBI) [Escherichia coli K12]	VIMSS16436	1.22	1.33	1.16	2.53	3.39	1.66
AroD	3-dehydroquinate dehydratase (NCBI) [Escherichia coli K12]	VIMSS15812	0	0	0	5.91	5.08	5.8
AroF	3-deoxy-D-arabino-heptulosonate-7-phosphate synthase, tyrosine-repressible (NCBI) [Escherichia coli K12]	VIMSS16696	94.08	131.69	87.31	135.97	155.08	163.27
AroG	3-deoxy-D-arabino-heptulosonate-7-phosphate synthase, phenylalanine repressible (NCBI) [Escherichia coli K12]	VIMSS14879	34.21	19.95	31.43	35.47	40.68	37.29
AroH	3-deoxy-D-arabino-heptulosonate-7-phosphate synthase, tryptophan repressible (NCBI) [Escherichia coli K12]	VIMSS15823	2.44	3.99	3.49	2.53	5.93	4.97
AroK	shikimate kinase I (NCBI) [Escherichia coli K12]	VIMSS1937004	4.89	5.32	3.49	3.38	4.24	4.14
AroL	shikimate kinase II (NCBI) [Escherichia coli K12]	VIMSS14526	0	0	0	3.38	2.54	2.49
ArtJ	arginine transporter subunit (NCBI) [Escherichia coli K12]	VIMSS14985	4.89	3.99	3.49	1.69	5.08	3.32
ArtP	arginine transporter subunit (NCBI) [Escherichia coli K12]	VIMSS14989	0	0	1.16	4.22	1.69	2.49
Asd	aspartate-semialdehyde dehydrogenase (NCBI) [Escherichia coli K12]	VIMSS17495	40.32	46.56	41.91	37.16	30.51	29.84
AsmA	predicted assembly protein (NCBI) [Escherichia coli K12]	VIMSS16172	0	1.33	0	0.84	0.85	2.49
AsnA	asparagine synthetase AsnA (NCBI) [Escherichia coli K12]	VIMSS17804	2.44	3.99	3.49	4.22	3.39	3.32
AsnB	asparagine synthetase B (NCBI) [Escherichia coli K12]	VIMSS14804	2.44	2.66	3.49	9.29	11.86	9.95
AsnS	asparaginyl-tRNA synthetase (NCBI) [Escherichia coli K12]	VIMSS15054	26.88	29.26	24.45	23.65	23.73	24.86
AspC	aspartate aminotransferase, PLP-dependent (NCBI) [Escherichia coli K12]	VIMSS15052	67.2	58.53	68.69	45.6	50	45.58
AspS	aspartyl-tRNA synthetase (NCBI) [Escherichia coli K12]	VIMSS15984	34.21	38.57	39.58	38	38.13	38.12
AtpA	F0F1 ATP synthase subunit alpha (NCBI) [Escherichia coli K12]	VIMSS17794	96.52	77.15	94.3	73.47	83.05	85.36
AtpC	F0F1 ATP synthase subunit epsilon (NCBI) [Escherichia coli K12]	VIMSS17791	3.67	5.32	4.66	5.07	4.24	4.14

AtpD	F0F1 ATP synthase subunit beta (NCBI) [Escherichia coli K12]	VIMSS17792	92.85	90.45	89.64	77.7	69.49	72.93
AtpF	F0F1 ATP synthase subunit B (NCBI) [Escherichia coli K12]	VIMSS17796	11	11.97	10.48	15.2	14.41	12.43
AtpG	F0F1 ATP synthase subunit gamma (NCBI) [Escherichia coli K12]	VIMSS17793	15.88	19.95	13.97	12.67	11.02	10.77
AtpH	F0F1 ATP synthase subunit delta (NCBI) [Escherichia coli K12]	VIMSS17795	9.77	13.3	11.64	10.13	14.41	10.77
AvtA	valine--pyruvate transaminase (NCBI) [Escherichia coli K12]	VIMSS1937060	0	0	1.16	2.53	3.39	3.32
AzoR	acyl carrier protein phosphodiesterase (NCBI) [Escherichia coli K12]	VIMSS15534	2.44	2.66	2.33	0.84	0.85	0.83
B96	5 orf, hypothetical protein (VIMSS) [Escherichia coli K12]	VIMSS15089	1.22	2.66	2.33	1.69	3.39	0.83
B96	7 putative oxidoreductase (VIMSS) [Escherichia coli K12]	VIMSS15091	1.22	0	2.33	2.53	0.85	0.83
B120	0 putative dihydroxyacetone kinase (EC 2.7.1.2) (VIMSS) [Escherichia coli K12]	VIMSS15322	3.67	2.66	1.16	2.53	3.39	1.66
B162	4 orf, hypothetical protein (VIMSS) [Escherichia coli K12]	VIMSS15745	3.67	3.99	5.82	5.91	4.24	4.97
B209	7 orf, hypothetical protein (VIMSS) [Escherichia coli K12]	VIMSS16204	0	0	0	0	1.69	0
B225	3 putative enzyme (VIMSS) [Escherichia coli K12]	VIMSS16360	15.88	15.96	15.13	8.45	10.17	6.63
B229	4 orf, hypothetical protein (VIMSS) [Escherichia coli K12]	VIMSS16401	11	10.64	13.97	10.98	11.86	15.75
B243	1 orf, hypothetical protein (VIMSS) [Escherichia coli K12]	VIMSS16530	3.67	2.66	4.66	2.53	5.93	5.8
B251	1 putative GTP-binding factor (VIMSS) [Escherichia coli K12]	VIMSS16610	11	5.32	6.99	9.29	6.78	9.12
B298	9 orf, hypothetical protein (VIMSS) [Escherichia coli K12]	VIMSS17067	2.44	1.33	1.16	1.69	1.69	2.49
B347	2 orf, hypothetical protein (VIMSS) [Escherichia coli K12]	VIMSS17533	4.89	3.99	5.82	3.38	4.24	5.8
BaeR	DNA-binding response regulator in two-component regulatory system with BaeS (NCBI) [Escherichia coli K12]	VIMSS16187	1.22	3.99	0	4.22	4.24	4.14
BasR	DNA-binding response regulator in two-component regulatory system with BasS (NCBI) [Escherichia coli K12]	VIMSS18141	3.67	6.65	4.66	4.22	3.39	2.49
Bcp	thioredoxin-dependent thiol peroxidase (NCBI) [Escherichia coli K12]	VIMSS16579	1.22	3.99	4.66	5.07	5.93	6.63
BetB	betaine aldehyde dehydrogenase, NAD-dependent (NCBI) [Escherichia coli K12]	VIMSS14450	7.33	6.65	5.82	5.07	6.78	7.46
BglA	6-phospho-beta-glucosidase A (NCBI) [Escherichia coli K12]	VIMSS16981	2.44	2.66	1.16	2.53	3.39	1.66
BglX	beta-D-glucoside glucohydrolase, periplasmic (NCBI) [Escherichia coli K12]	VIMSS16240	1.22	1.33	3.49	3.38	3.39	4.97
BioD	dethiobiotin synthetase (NCBI) [Escherichia coli K12]	VIMSS14903	1.22	2.66	2.33	1.69	3.39	3.32
BioH	carboxylesterase of pimeloyl-CoA synthesis (NCBI) [Escherichia coli K12]	VIMSS17475	0	1.33	0	1.69	0	0.83
BipA	GTP-binding protein (NCBI) [Escherichia coli K12]	VIMSS1937169	14.66	15.96	19.79	15.2	22.03	19.89
CafA	bundles of cytoplasmic filaments (VIMSS) [Escherichia coli K12]	VIMSS17317	1.22	2.66	0	2.53	1.69	1.66
Can	carbonic anhydrase (NCBI) [Escherichia coli K12]	VIMSS14272	4.89	2.66	3.49	5.91	5.08	5.8
CarA	carbamoyl-phosphate synthase small subunit (NCBI) [Escherichia coli K12]	VIMSS14177	12.22	9.31	9.31	11.82	11.02	7.46
CarB	carbamoyl-phosphate synthase large subunit (NCBI) [Escherichia coli K12]	VIMSS14178	36.65	41.23	31.43	38	37.29	32.32
Cdd	cytidine deaminase (NCBI) [Escherichia coli K12]	VIMSS16252	0	0	0	1.69	1.69	1.66
Chlo	ramphenicol acetyltransferase (CAM)	gi|50401831|C	174.71	184.89	176.96	52.36	59.32	57.19
ClpA	ATPase and specificity subunit of ClpA-ClpP ATP-dependent serine protease, chaperone activity (NCBI) [Escherichia coli K12]	VIMSS15007	3.67	5.32	5.82	4.22	3.39	5.8
ClpB	protein disaggregation chaperone (NCBI) [Escherichia coli K12]	VIMSS16687	36.65	38.57	32.6	35.47	42.37	46.41
ClpP	ATP-dependent Clp protease proteolytic subunit (NCBI) [Escherichia coli K12]	VIMSS14575	6.11	6.65	4.66	6.76	10.17	8.29
ClpX	ATP-dependent protease ATP-binding subunit (NCBI) [Escherichia coli K12]	VIMSS14576	4.89	0	4.66	5.07	4.24	4.97
Cls	cardiolipin synthetase (NCBI) [Escherichia coli K12]	VIMSS15369	2.44	1.33	2.33	1.69	2.54	2.49
Cmk	cytidylate kinase (NCBI) [Escherichia coli K12]	VIMSS15034	1.22	1.33	0	0.84	1.69	1.66
CoaD	phosphopantetheine adenylyltransferase (NCBI) [Escherichia coli K12]	VIMSS17695	0	0	0	0.84	1.69	1.66
CobT	nicotinate-nucleotide--dimethylbenzimidazolephosphoribosyltransferase (NCBI) [Escherichia coli K12]	VIMSS16099	0	0	0	0	1.69	0
CodA	cytosine deaminase (NCBI) [Escherichia coli K12]	VIMSS14475	1.22	1.33	1.16	4.22	4.24	3.32
CopA	copper transporter (NCBI) [Escherichia coli K12]	VIMSS14621	0	0	0	0.84	1.69	1.66
CorA	magnesium/nickel/cobalt transporter (NCBI) [Escherichia coli K12]	VIMSS17864	1.22	1.33	2.33	1.69	1.69	1.66
CpdB	bifunctional 2',3'-cyclic nucleotide 2'-phosphodiesterase/3'-nucleotidase periplasmic precursor protein (NCBI) [Escherichia coli K12]	VIMSS18238	3.67	1.33	1.16	3.38	3.39	4.14
CpxR	DNA-binding response regulator in two-component regulatory system with CpxA (NCBI) [Escherichia coli K12]	VIMSS17952	0	0	2.33	2.53	2.54	2.49
CreA	hypothetical protein (NCBI) [Escherichia coli K12]	VIMSS18418	2.44	1.33	1.16	0.84	1.69	0.83
Crp	DNA-binding transcriptional dual regulator (NCBI) [Escherichia coli K12]	VIMSS17420	15.88	9.31	17.46	8.45	10.17	8.29
Crr	glucose-specific PTS system enzyme IIA component (NCBI) [Escherichia coli K12]	VIMSS16516	28.1	29.26	29.1	23.65	25.42	29.84
CspC	stress protein, member of the CspA-family (NCBI) [Escherichia coli K12]	VIMSS15941	13.44	9.31	11.64	8.45	7.63	5.8
CueO	multicopper oxidase (laccase) (NCBI) [Escherichia coli K12]	VIMSS14269	0	0	0	1.69	0.85	0.83
CybC	cytochrome b(562) (VIMSS) [Escherichia coli K12]	VIMSS18261	1.22	2.66	2.33	3.38	5.08	3.32
CydA	cytochrome d terminal oxidase, polypeptide subunit I (VIMSS) [Escherichia coli K12]	VIMSS14865	2.44	1.33	3.49	0.84	0.85	3.32
CyoA	cytochrome o ubiquinol oxidase subunit II (NCBI) [Escherichia coli K12]	VIMSS14570	9.77	9.31	11.64	5.91	6.78	8.29
CyoB	cytochrome o ubiquinol oxidase subunit I (NCBI) [Escherichia coli K12]	VIMSS14569	3.67	6.65	3.49	2.53	3.39	1.66
CysA	sulfate/thiosulfate transporter subunit (NCBI) [Escherichia coli K12]	VIMSS16521	7.33	6.65	3.49	0.84	0.85	0.83
CysB	DNA-binding transcriptional dual regulator, O-acetyl-L-serine-binding (NCBI) [Escherichia coli K12]	VIMSS15395	1.22	0	1.16	0	2.54	3.32
CysC	adenylylsulfate kinase (NCBI) [Escherichia coli K12]	VIMSS16834	0	0	0	1.69	0.85	1.66
CysD	sulfate adenylyltransferase subunit 2 (NCBI) [Escherichia coli K12]	VIMSS16836	8.55	5.32	8.15	4.22	3.39	3.32
CysE	serine acetyltransferase (NCBI) [Escherichia coli K12]	VIMSS17668	3.67	3.99	3.49	5.07	4.24	5.8
CysH	phosphoadenosine phosphosulfate reductase (NCBI) [Escherichia coli K12]	VIMSS16846	21.99	26.6	23.28	16.05	17.8	14.92
CysI	sulfite reductase, beta subunit, NAD(P)-binding, heme-binding (NCBI) [Escherichia coli K12]	VIMSS16847	43.98	45.23	41.91	38	27.12	29.01
CysJ	sulfite reductase, alpha subunit, flavoprotein (NCBI) [Escherichia coli K12]	VIMSS16848	31.77	43.9	33.76	38	32.2	32.32

CysK	cysteine synthase A, O-acetylserine sulfhydrolase A subunit (NCBI) [Escherichia coli K12]	VIMSS16513	139.28	144.99	132.72	114.86	116.1	118.51
CysM	cysteine synthase B (O-acetylserine sulfhydrolase B) (NCBI) [Escherichia coli K12]	VIMSS16520	4.89	6.65	6.99	4.22	3.39	3.32
CysN	sulfate adenylyltransferase subunit 1 (NCBI) [Escherichia coli K12]	VIMSS16835	18.33	34.58	25.61	13.51	11.02	11.6
CysP	thiosulfate transporter subunit (NCBI) [Escherichia coli K12]	VIMSS16524	23.21	19.95	19.79	19.42	23.73	28.18
CysQ	PAPS (adenosine 3'-phosphate 5'-phosphosulfate) 3'(2'),5'-bisphosphate nucleotidase (NCBI) [Escherichia coli K12]	VIMSS18239	1.22	0	0	2.53	2.54	1.66
CysS	cysteinyl-tRNA synthetase (NCBI) [Escherichia coli K12]	VIMSS14663	2.44	2.66	1.16	5.07	3.39	5.8
DacA	D-alanyl-D-alanine carboxypeptidase (penicillin-binding protein 5) (NCBI) [Escherichia coli K12]	VIMSS14769	7.33	6.65	10.48	8.45	6.78	9.12
DacC	D-alanyl-D-alanine carboxypeptidase (penicillin-binding protein 6a) (NCBI) [Escherichia coli K12]	VIMSS14964	3.67	5.32	4.66	6.76	4.24	8.29
DadA	D-amino acid dehydrogenase small subunit (NCBI) [Escherichia coli K12]	VIMSS15311	6.11	5.32	5.82	10.13	3.39	7.46
DadX	alanine racemase (NCBI) [Escherichia coli K12]	VIMSS15312	2.44	1.33	3.49	4.22	4.24	4.97
DamX	hypothetical protein (NCBI) [Escherichia coli K12]	VIMSS17451	2.44	0	0	0	0	0
DapA	dihydrodipicolinate synthase (NCBI) [Escherichia coli K12]	VIMSS16577	9.77	15.96	11.64	10.13	8.47	8.29
DapB	dihydrodipicolinate reductase (NCBI) [Escherichia coli K12]	VIMSS14176	6.11	6.65	10.48	10.13	8.47	7.46
DapD	2,3,4,5-tetrahydropyridine-2-carboxylate N-succinyltransferase (NCBI) [Escherichia coli K12]	VIMSS14312	25.66	45.23	25.61	26.18	33.05	33.98
DapF	diaminopimelate epimerase (VIMSS) [Escherichia coli K12]	VIMSS17858	0	0	0	0	0.85	1.66
Dcd	deoxycytidine triphosphate deaminase (NCBI) [Escherichia coli K12]	VIMSS16173	4.89	3.99	4.66	1.69	0.85	0.83
Dcp	dipeptidyl carboxypeptidase II (NCBI) [Escherichia coli K12]	VIMSS15660	1.22	0	4.66	1.69	3.39	2.49
DdlA	D-alanylalanine synthetase (NCBI) [Escherichia coli K12]	VIMSS14519	3.67	3.99	4.66	3.38	4.24	4.97
DdlB	D-alanylalanine synthetase (NCBI) [Escherichia coli K12]	VIMSS14238	2.44	2.66	2.33	2.53	2.54	4.97
DeaD	inducible ATP-independent RNA helicase (VIMSS) [Escherichia coli K12]	VIMSS17235	0	0	1.16	4.22	4.24	3.32
Def	peptide deformylase (NCBI) [Escherichia coli K12]	VIMSS17349	1.22	1.33	2.33	2.53	2.54	2.49
DegP	serine endoprotease (protease Do), membrane-associated (NCBI) [Escherichia coli K12]	VIMSS14307	7.33	6.65	6.99	6.76	8.47	7.46
DegQ	serine endoprotease, periplasmic (NCBI) [Escherichia coli K12]	VIMSS17305	3.67	5.32	4.66	3.38	8.47	4.14
DeoA	thymidine phosphorylase (NCBI) [Escherichia coli K12]	VIMSS18403	0	0	0	4.22	5.08	2.49
DeoB	phosphopentomutase (NCBI) [Escherichia coli K12]	VIMSS18404	0	0	0	2.53	1.69	4.14
DeoC	deoxyribose-phosphate aldolase (NCBI) [Escherichia coli K12]	VIMSS18402	2.44	0	1.16	5.07	4.24	4.14
DeoD	purine nucleoside phosphorylase (NCBI) [Escherichia coli K12]	VIMSS18405	8.55	9.31	11.64	5.91	8.47	9.95
Dfp	flavoprotein affecting synthesis of DNA and pantothenate metabolism (VIMSS) [Escherichia coli K12]	VIMSS17700	1.22	0	0	5.91	5.93	5.8
DhaL	dihydroxyacetone kinase, C-terminal domain (NCBI) [Escherichia coli K12]	VIMSS15321	2.44	1.33	0	2.53	2.54	2.49
DksA	DNA-binding transcriptional regulator of rRNA transcription, DnaK suppressor protein (NCBI) [Escherichia coli K12]	VIMSS14291	6.11	10.64	11.64	4.22	3.39	3.32
DnaB	replicative DNA helicase (NCBI) [Escherichia coli K12]	VIMSS18080	3.67	1.33	1.16	1.69	1.69	2.49
DnaK	molecular chaperone DnaK (NCBI) [Escherichia coli K12]	VIMSS14159	40.32	30.59	38.42	39.69	38.98	33.98
DnaN	DNA polymerase III subunit beta (NCBI) [Escherichia coli K12]	VIMSS17760	1.22	1.33	2.33	2.53	4.24	4.14
DnaX	DNA polymerase III subunits gamma and tau (NCBI) [Escherichia coli K12]	VIMSS14607	1.22	1.33	0	4.22	2.54	1.66
DppA	dipeptide transporter (NCBI) [Escherichia coli K12]	VIMSS17605	24.44	21.28	22.12	27.02	27.12	26.52
DppD	dipeptide transporter (NCBI) [Escherichia coli K12]	VIMSS17602	0	1.33	0	1.69	1.69	1.66
DppF	dipeptide transporter (NCBI) [Escherichia coli K12]	VIMSS17601	1.22	1.33	0	2.53	1.69	2.49
Dps	DNA protection during starvation conditions (NCBI) [Escherichia coli K12]	VIMSS14937	7.33	6.65	6.99	5.91	7.63	5.8
Dut	deoxyuridine 5'-triphosphate nucleotidohydrolase (NCBI) [Escherichia coli K12]	VIMSS17701	1.22	2.66	2.33	0.84	1.69	1.66
Dxs	1-deoxy-D-xylulose-5-phosphate synthase (NCBI) [Escherichia coli K12]	VIMSS14558	1.22	2.66	1.16	2.53	3.39	4.14
Eda	keto-hydroxyglutarate-aldolase/keto-deoxy- phosphogluconate aldolase (NCBI) [Escherichia coli K12]	VIMSS15968	8.55	5.32	9.31	8.45	6.78	8.29
Efp	elongation factor P (NCBI) [Escherichia coli K12]	VIMSS18175	4.89	3.99	4.66	2.53	2.54	2.49
Eno	phosphopyruvate hydratase (NCBI) [Escherichia coli K12]	VIMSS16863	68.42	61.19	71.02	98.81	95.76	106.91
FabA	3-hydroxydecanoyl-ACP dehydratase (NCBI) [Escherichia coli K12]	VIMSS15078	3.67	5.32	4.66	5.91	5.93	5.8
FabB	3-oxoacyl-(acyl carrier protein) synthase (NCBI) [Escherichia coli K12]	VIMSS16430	20.77	11.97	22.12	31.25	26.27	30.66
FabD	acyl carrier protein S-malonyltransferase (NCBI) [Escherichia coli K12]	VIMSS15214	9.77	6.65	10.48	6.76	5.08	4.97
FabF	3-oxoacyl-(acyl carrier protein) synthase (NCBI) [Escherichia coli K12]	VIMSS15217	20.77	18.62	16.3	21.96	20.34	19.06
FabG	3-oxoacyl-[acyl-carrier-protein] reductase (NCBI) [Escherichia coli K12]	VIMSS15215	20.77	15.96	18.63	13.51	16.1	11.6
FabH	3-oxoacyl-(acyl carrier protein) synthase (NCBI) [Escherichia coli K12]	VIMSS15213	7.33	11.97	10.48	9.29	10.17	7.46
FabI	enoyl-(acyl carrier protein) reductase (NCBI) [Escherichia coli K12]	VIMSS15408	28.1	23.94	23.28	16.05	13.56	17.4
FabR	DNA-binding transcriptional repressor (NCBI) [Escherichia coli K12]	VIMSS18002	0	0	0	0	0.85	1.66
FabZ	(3R)-hydroxymyristoyl ACP dehydratase (NCBI) [Escherichia coli K12]	VIMSS14326	8.55	3.99	6.99	10.98	10.17	9.95
FbaA	fructose-bisphosphate aldolase (NCBI) [Escherichia coli K12]	VIMSS17004	26.88	37.24	31.43	25.34	26.27	26.52
Fbp	fructose-1,6-bisphosphatase (NCBI) [Escherichia coli K12]	VIMSS18257	4.89	3.99	3.49	1.69	2.54	2.49
Fdx	[2Fe-2S] ferredoxin (NCBI) [Escherichia coli K12]	VIMSS16624	6.11	5.32	6.99	5.07	4.24	6.63
Ffh	Signal Recognition Particle (SRP) component with 4.5S RNA (ffs) (NCBI) [Escherichia coli K12]	VIMSS16705	4.89	1.33	5.82	7.6	6.78	5.8
FklB	FKBP-type 22KD peptidyl-prolyl cis-trans isomerase (rotamase) (VIMSS) [Escherichia coli K12]	VIMSS18232	2.44	5.32	4.66	1.69	2.54	2.49
FkpA	FKBP-type peptidyl-prolyl cis-trans isomerase (rotamase) (NCBI) [Escherichia coli K12]	VIMSS17410	12.22	7.98	11.64	8.45	7.63	9.12
Fmt	methionyl-tRNA formyltransferase (NCBI) [Escherichia coli K12]	VIMSS17350	4.89	5.32	3.49	0.84	0.85	1.66
FolC	bifunctional folylpolyglutamate synthase/ dihydrofolate synthase (NCBI) [Escherichia coli K12]	VIMSS16422	1.22	1.33	3.49	5.07	2.54	3.32
FolD	bifunctional 5,10-methylene-tetrahydrofolate dehydrogenase/ 5,10-methylene-tetrahydrofolate cyclohydrolase (NCBI) [Escherichia coli K12]	VIMSS14666	3.67	0	2.33	1.69	1.69	1.66
FolE	GTP cyclohydrolase I (NCBI) [Escherichia coli K12]	VIMSS16262	15.88	11.97	15.13	29.56	22.03	26.52

FolX	D-erythro-7,8-dihydroneopterin triphosphate 2'-epimerase and dihydroneopterin aldolase (NCBI) [Escherichia coli K12]	VIMSS16410	1.22	1.33	2.33	1.69	2.54	0.83
Fpr	ferredoxin-NADP reductase (NCBI) [Escherichia coli K12]	VIMSS17963	0	0	0	1.69	0.85	0.83
Fre	NAD(P)H-flavin reductase (NCBI) [Escherichia coli K12]	VIMSS17890	2.44	3.99	2.33	0.84	1.69	1.66
FrmA	alcohol dehydrogenase class III/glutathione-dependent formaldehyde dehydrogenase (NCBI) [Escherichia coli K12]	VIMSS14494	0	0	0	0.84	0	1.66
Frr	ribosome releasing factor (NCBI) [Escherichia coli K12]	VIMSS14318	7.33	5.32	4.66	4.22	6.78	8.29
FrsA	fermentation/respiration switch protein (NCBI) [Escherichia coli K12]	VIMSS14378	1.22	1.33	0	1.69	0.85	1.66
FruR	DNA-binding transcriptional dual regulator (NCBI) [Escherichia coli K12]	VIMSS14226	1.22	0	2.33	0.84	0.85	2.49
Ftn	ferritin iron storage protein (cytoplasmic) (NCBI) [Escherichia coli K12]	VIMSS16022	7.33	10.64	11.64	7.6	5.93	8.29
FtsA	cell division protein (NCBI) [Escherichia coli K12]	VIMSS14240	4.89	3.99	2.33	2.53	2.54	2.49
FtsE	predicted transporter subunit: ATP-binding component of ABC superfamily (NCBI) [Escherichia coli K12]	VIMSS17524	2.44	1.33	0	1.69	1.69	0.83
FtsH	protease, ATP-dependent zinc-metallo (NCBI) [Escherichia coli K12]	VIMSS17249	25.66	31.92	24.45	28.71	22.88	21.55
FtsN	essential cell division protein (NCBI) [Escherichia coli K12]	VIMSS17972	1.22	2.66	1.16	0.84	0.85	0
FtsY	fused Signal Recognition Particle (SRP) receptor: membrane binding protein/conserved protein (NCBI) [Escherichia coli K12]	VIMSS17525	3.67	1.33	2.33	6.76	4.24	9.12
FtsZ	cell division protein FtsZ (NCBI) [Escherichia coli K12]	VIMSS14241	15.88	11.97	16.3	26.18	24.58	22.38
FucI	L-fucose isomerase (NCBI) [Escherichia coli K12]	VIMSS16886	0	0	3.49	2.53	3.39	4.14
FucU	L-fucose mutarotase (NCBI) [Escherichia coli K12]	VIMSS16888	4.89	6.65	4.66	1.69	2.54	3.32
FumA	fumarate hydratase (fumarase A), aerobic Class I (NCBI) [Escherichia coli K12]	VIMSS15733	15.88	29.26	19.79	17.74	16.1	13.26
FumC	fumarate hydratase (NCBI) [Escherichia coli K12]	VIMSS15732	1.22	1.33	2.33	5.91	5.08	4.14
Fur	ferric uptake regulator (NCBI) [Escherichia coli K12]	VIMSS14813	2.44	2.66	1.16	2.53	2.54	4.14
FusA	elongation factor EF-2 (NCBI) [Escherichia coli K12]	VIMSS17403	113.62	121.04	124.57	126.68	108.47	120.17
GabT	4-aminobutyrate aminotransferase (NCBI) [Escherichia coli K12]	VIMSS16752	0	1.33	0	0.84	0.85	2.49
GalF	predicted subunit with GalU (NCBI) [Escherichia coli K12]	VIMSS16150	4.89	3.99	3.49	4.22	5.93	2.49
GalU	glucose-1-phosphate uridylyltransferase (NCBI) [Escherichia coli K12]	VIMSS15356	7.33	6.65	8.15	7.6	6.78	6.63
GapA	glyceraldehyde-3-phosphate dehydrogenase (NCBI) [Escherichia coli K12]	VIMSS15897	18.33	17.29	19.79	38	40.68	33.98
GatZ	D-tagatose 1,6-bisphosphate aldolase 2, subunit (NCBI) [Escherichia coli K12]	VIMSS16202	4.89	2.66	4.66	0.84	1.69	0.83
Gcd	glucose dehydrogenase (NCBI) [Escherichia coli K12]	VIMSS14270	2.44	2.66	0	8.45	8.47	7.46
GcvH	glycine cleavage system protein H (NCBI) [Escherichia coli K12]	VIMSS16984	1.22	0	2.33	0	1.69	0.83
GcvP	glycine dehydrogenase (NCBI) [Escherichia coli K12]	VIMSS16983	1.22	1.33	0	3.38	4.24	4.14
GcvR	transcriptional regulation of gcv operon (VIMSS) [Escherichia coli K12]	VIMSS16578	0	0	0	1.69	1.69	0.83
GcvT	glycine cleavage system aminomethyltransferase T (NCBI) [Escherichia coli K12]	VIMSS16985	1.22	0	0	4.22	1.69	2.49
GdhA	glutamate dehydrogenase (NCBI) [Escherichia coli K12]	VIMSS15879	34.21	39.9	29.1	27.87	25.42	29.84
GidA	glucose-inhibited division protein A (NCBI) [Escherichia coli K12]	VIMSS17801	0	0	0	2.53	0	0
GidB	glucose-inhibited division protein B (NCBI) [Escherichia coli K12]	VIMSS17800	1.22	0	0	1.69	1.69	0.83
GlcB	malate synthase (NCBI) [Escherichia coli K12]	VIMSS17054	0	1.33	0	0.84	2.54	1.66
GlgA	glycogen synthase (NCBI) [Escherichia coli K12]	VIMSS17491	11	6.65	10.48	7.6	3.39	4.14
GlgB	glycogen branching enzyme (NCBI) [Escherichia coli K12]	VIMSS17494	14.66	14.63	12.81	0.84	0.85	0.83
GlgC	glucose-1-phosphate adenylyltransferase (NCBI) [Escherichia coli K12]	VIMSS17492	15.88	15.96	17.46	3.38	4.24	3.32
GlgP	glycogen phosphorylase (NCBI) [Escherichia coli K12]	VIMSS17490	2.44	2.66	4.66	0.84	3.39	0.83
Glk	glucokinase (NCBI) [Escherichia coli K12]	VIMSS16493	3.67	2.66	4.66	3.38	2.54	2.49
GlmM	phosphoglucosamine mutase (NCBI) [Escherichia coli K12]	VIMSS17247	8.55	5.32	5.82	7.6	11.02	9.12
GlmS	D-fructose-6-phosphate amidotransferase (NCBI) [Escherichia coli K12]	VIMSS17789	15.88	17.29	19.79	16.05	15.25	16.58
GlmU	bifunctional N-acetylglucosamine-1-phosphate uridyltransferase/glucosamine-1-phosphate acetyltransferase (NCBI) [Escherichia coli K12]	VIMSS17790	0	0	0	0	1.69	1.66
GlnA	glutamine synthetase (NCBI) [Escherichia coli K12]	VIMSS17910	6.11	9.31	11.64	12.67	9.32	10.77
GlnB	regulatory protein P-II for glutamine synthetase (NCBI) [Escherichia coli K12]	VIMSS16652	0	0	0	0.84	1.69	2.49
GlnE	fused deadenylyltransferase/adenylyltransferase for glutamine synthetase (NCBI) [Escherichia coli K12]	VIMSS17129	3.67	1.33	2.33	1.69	2.54	4.14
GlnH	glutamine ABC transporter periplasmic protein (NCBI) [Escherichia coli K12]	VIMSS14936	7.33	9.31	5.82	10.13	8.47	9.95
GlnQ	glutamine ABC transporter ATP-binding protein (NCBI) [Escherichia coli K12]	VIMSS14934	1.22	1.33	1.16	1.69	2.54	2.49
GlnS	glutaminyl-tRNA synthetase (NCBI) [Escherichia coli K12]	VIMSS14810	4.89	9.31	6.99	9.29	5.93	9.12
GlpK	glycerol kinase (NCBI) [Escherichia coli K12]	VIMSS17965	0	0	0	2.53	0	0
GlpR	DNA-binding transcriptional repressor (NCBI) [Escherichia coli K12]	VIMSS17485	1.22	0	4.66	0	0	0
GltA	citrate synthase (NCBI) [Escherichia coli K12]	VIMSS14852	54.98	42.56	51.22	66.72	66.1	61.33
GltB	glutamate synthase, large subunit (NCBI) [Escherichia coli K12]	VIMSS17283	35.43	35.91	33.76	59.96	61.01	58.84
GltD	glutamate synthase, 4Fe-4S protein, small subunit (NCBI) [Escherichia coli K12]	VIMSS17284	3.67	7.98	4.66	11.82	15.25	11.6
GltI	glutamate and aspartate transporter subunit (NCBI) [Escherichia coli K12]	VIMSS14792	2.44	3.99	4.66	6.76	5.93	7.46
GltX	glutamyl-tRNA synthetase (NCBI) [Escherichia coli K12]	VIMSS16503	4.89	9.31	4.66	12.67	14.41	10.77
GlyA	serine hydroxymethyltransferase (NCBI) [Escherichia coli K12]	VIMSS16650	45.2	43.9	37.25	41.38	39.83	45.58
GlyQ	glycyl-tRNA synthetase subunit alpha (NCBI) [Escherichia coli K12]	VIMSS17621	4.89	0	3.49	3.38	3.39	4.14
GlyS	glycyl-tRNA synthetase subunit beta (NCBI) [Escherichia coli K12]	VIMSS17620	17.1	13.3	20.96	24.49	15.25	22.38
Gmk	guanylate kinase (NCBI) [Escherichia coli K12]	VIMSS17709	3.67	2.66	3.49	2.53	5.08	4.14
Gnd	6-phosphogluconate dehydrogenase (NCBI) [Escherichia coli K12]	VIMSS16137	31.77	33.25	37.25	30.4	30.51	33.15
GntK	gluconokinase 2, thermoresistant (VIMSS) [Escherichia coli K12]	VIMSS17498	0	0	1.16	4.22	1.69	2.49
Gor	glutathione reductase (NCBI) [Escherichia coli K12]	VIMSS17561	2.44	1.33	3.49	2.53	5.08	3.32

Gph	phosphoglycolate phosphatase (NCBI) [Escherichia coli K12]	VIMSS17448	2.44	2.66	1.16	5.07	2.54	1.66
GpmA	phosphoglyceromutase (NCBI) [Escherichia coli K12]	VIMSS14880	43.98	42.56	39.58	39.69	37.29	30.66
GpmI	phosphoglyceromutase (NCBI) [Escherichia coli K12]	VIMSS17673	1.22	1.33	2.33	5.91	9.32	6.63
GpsA	NAD(P)H-dependent glycerol-3-phosphate dehydrogenase (NCBI) [Escherichia coli K12]	VIMSS17669	1.22	1.33	0	1.69	0.85	2.49
GreA	transcription elongation factor: cleaves 3' nucleotide of paused mRNA (VIMSS) [Escherichia coli K12]	VIMSS17252	3.67	3.99	4.66	3.38	2.54	7.46
GroEL	L chaperonin GroEL (NCBI) [Escherichia coli K12]	VIMSS18171	101.41	105.08	98.96	124.99	127.11	134.26
GroS	co-chaperonin GroES (NCBI) [Escherichia coli K12]	VIMSS18170	13.44	23.94	13.97	19.42	19.49	17.4
GrpE	heat shock protein (NCBI) [Escherichia coli K12]	VIMSS16708	7.33	7.98	6.99	1.69	3.39	4.14
GrxB	glutaredoxin 2 (Grx2) (NCBI) [Escherichia coli K12]	VIMSS15186	3.67	3.99	3.49	3.38	6.78	4.97
GrxC	glutaredoxin 3 (NCBI) [Escherichia coli K12]	VIMSS17671	8.55	2.66	6.99	5.07	4.24	5.8
GshA	glutamate--cysteine ligase (NCBI) [Escherichia coli K12]	VIMSS16777	4.89	5.32	4.66	6.76	4.24	6.63
GshB	glutathione synthetase (NCBI) [Escherichia coli K12]	VIMSS17026	8.55	6.65	5.82	10.98	13.56	16.58
Gss	fused glutathionylspermidine amidase/glutathionylspermidine synthetase (NCBI) [Escherichia coli K12]	VIMSS17066	1.22	1.33	1.16	3.38	3.39	3.32
Gst	glutathionine S-transferase (NCBI) [Escherichia coli K12]	VIMSS15756	19.55	29.26	19.79	11.82	11.86	11.6
GuaA	bifunctional GMP synthase/glutamine amidotransferase protein (NCBI) [Escherichia coli K12]	VIMSS16606	12.22	9.31	13.97	11.82	11.86	10.77
GuaB	inositol-5-monophosphate dehydrogenase (NCBI) [Escherichia coli K12]	VIMSS16607	9.77	11.97	13.97	17.74	20.34	15.75
GuaC	guanosine 5'-monophosphate oxidoreductase (NCBI) [Escherichia coli K12]	VIMSS14250	1.22	2.66	2.33	0.84	1.69	0.83
GutQ	orf, hypothetical protein (VIMSS) [Escherichia coli K12]	VIMSS16792	9.77	7.98	8.15	12.67	12.71	13.26
GyrA	DNA gyrase subunit A (NCBI) [Escherichia coli K12]	VIMSS16338	9.77	3.99	6.99	10.98	14.41	11.6
GyrB	DNA gyrase subunit B (NCBI) [Escherichia coli K12]	VIMSS1937098	7.33	10.64	8.15	10.13	12.71	9.95
Hda	DNA replication initiation factor (NCBI) [Escherichia coli K12]	VIMSS16595	0	0	0	1.69	0.85	0.83
HemB	5-aminolevulinate dehydratase = porphobilinogen synthase (VIMSS) [Escherichia coli K12]	VIMSS14507	2.44	2.66	2.33	4.22	3.39	4.97
HemC	porphobilinogen deaminase (NCBI) [Escherichia coli K12]	VIMSS1937139	3.67	5.32	3.49	2.53	2.54	4.14
HemE	uroporphyrinogen decarboxylase (NCBI) [Escherichia coli K12]	VIMSS18029	1.22	1.33	3.49	1.69	2.54	0.83
HemL	glutamate-1-semialdehyde aminotransferase (NCBI) [Escherichia coli K12]	VIMSS14300	21.99	19.95	18.63	18.58	22.03	23.21
HemN	O2-independent coproporphyrinogen III oxidase (VIMSS) [Escherichia coli K12]	VIMSS17907	0	1.33	2.33	5.07	1.69	4.14
HemX	predicted uroporphyrinogen III methylase (NCBI) [Escherichia coli K12]	VIMSS17851	11	7.98	8.15	6.76	6.78	4.97
HemY	predicted protoheme IX synthesis protein (NCBI) [Escherichia coli K12]	VIMSS17850	4.89	2.66	1.16	0.84	2.54	2.49
HepA	ATP-dependent helicase HepA (NCBI) [Escherichia coli K12]	VIMSS14205	0	0	0	1.69	2.54	0.83
HflC	modulator for HflB protease specific for phage lambda cII repressor (NCBI) [Escherichia coli K12]	VIMSS18200	14.66	18.62	15.13	7.6	10.17	7.46
HflK	modulator for HflB protease specific for phage lambda cII repressor (NCBI) [Escherichia coli K12]	VIMSS18199	11	10.64	17.46	7.6	8.47	7.46
HflX	predicted GTPase (NCBI) [Escherichia coli K12]	VIMSS18198	1.22	0	0	3.38	2.54	4.14
HisA	N-(5'-phospho-L-ribosyl-formimino)-5-amino-1-(5'- phosphoribosyl)-4-imidazolecarboxamide isomerase (VIMSS) [Escherichia coli K12]	VIMSS16132	11	9.31	8.15	13.51	12.71	14.92
HisB	imidazoleglycerolphosphate dehydratase and histidinol-phosphate phosphatase (VIMSS) [Escherichia coli K12]	VIMSS16130	3.67	6.65	4.66	5.07	5.93	7.46
HisC	histidinol-phosphate aminotransferase (NCBI) [Escherichia coli K12]	VIMSS16129	11	3.99	10.48	6.76	10.17	8.29
HisD	histidinol dehydrogenase (NCBI) [Escherichia coli K12]	VIMSS16128	6.11	3.99	4.66	9.29	9.32	9.12
HisF	imidazole glycerol phosphate synthase subunit HisF (NCBI) [Escherichia coli K12]	VIMSS16133	12.22	19.95	11.64	15.2	12.71	13.26
HisG	ATP phosphoribosyltransferase (NCBI) [Escherichia coli K12]	VIMSS16127	6.11	6.65	9.31	5.91	11.02	9.12
HisH	imidazole glycerol phosphate synthase subunit HisH (NCBI) [Escherichia coli K12]	VIMSS16131	4.89	0	3.49	2.53	4.24	4.97
HisJ	histidine/lysine/arginine/ornithine transporter subunit (NCBI) [Escherichia coli K12]	VIMSS16416	8.55	11.97	9.31	11.82	11.02	10.77
HisS	histidyl-tRNA synthetase (NCBI) [Escherichia coli K12]	VIMSS16613	18.33	21.28	20.96	19.42	18.64	20.72
HlpA	periplasmic chaperone (NCBI) [Escherichia coli K12]	VIMSS14324	12.22	11.97	9.31	8.45	7.63	10.77
Hmp	fused nitric oxide dioxygenase/dihydropteridine reductase 2 (NCBI) [Escherichia coli K12]	VIMSS16651	0	0	0	1.69	0.85	0
Hns	global DNA-binding transcriptional dual regulator H-NS (NCBI) [Escherichia coli K12]	VIMSS15357	42.76	37.24	47.73	25.34	38.98	38.12
Hpt	hypoxanthine phosphoribosyltransferase (VIMSS) [Escherichia coli K12]	VIMSS14271	2.44	6.65	2.33	2.53	2.54	1.66
HscA	chaperone protein HscA (NCBI) [Escherichia coli K12]	VIMSS16625	7.33	14.63	10.48	10.98	8.47	9.95
HslU	ATP-dependent protease ATP-binding subunit (NCBI) [Escherichia coli K12]	VIMSS17970	13.44	18.62	13.97	14.36	12.71	14.09
HslV	ATP-dependent protease peptidase subunit (NCBI) [Escherichia coli K12]	VIMSS17971	2.44	2.66	3.49	5.91	4.24	5.8
HtpG	heat shock protein 90 (NCBI) [Escherichia coli K12]	VIMSS14610	24.44	21.28	25.61	16.89	20.34	19.89
HtrG	predicted signal transduction protein (SH3 domain) (NCBI) [Escherichia coli K12]	VIMSS17131	2.44	0	0	0.84	0	0
HupA	HU, DNA-binding transcriptional regulator, alpha subunit (NCBI) [Escherichia coli K12]	VIMSS18032	51.31	31.92	32.6	35.47	29.66	29.84
HupB	HU, DNA-binding transcriptional regulator, beta subunit (NCBI) [Escherichia coli K12]	VIMSS14578	3.67	5.32	11.64	10.13	7.63	9.12
HybC	hydrogenase 2, large subunit (NCBI) [Escherichia coli K12]	VIMSS17072	1.22	1.33	3.49	0	0.85	0.83
IaaA	L-asparaginase (NCBI) [Escherichia coli K12]	VIMSS14953	4.89	7.98	3.49	1.69	2.54	0.83
IadA	isoaspartyl dipeptidase (NCBI) [Escherichia coli K12]	VIMSS18352	4.89	1.33	4.66	0.84	1.69	2.49
IbpA	heat shock chaperone (NCBI) [Escherichia coli K12]	VIMSS17746	4.89	3.99	2.33	2.53	1.69	1.66
Icd	isocitrate dehydrogenase (NCBI) [Escherichia coli K12]	VIMSS15258	119.73	113.06	118.75	78.54	81.35	79.56
IhfA	integration host factor subunit alpha (NCBI) [Escherichia coli K12]	VIMSS15831	9.77	10.64	10.48	7.6	12.71	9.95
IhfB	integration host factor subunit beta (NCBI) [Escherichia coli K12]	VIMSS15036	13.44	11.97	8.15	8.45	5.93	5.8
IleS	isoleucyl-tRNA synthetase (NCBI) [Escherichia coli K12]	VIMSS14171	20.77	14.63	26.78	21.11	27.96	28.18
IlvA	threonine dehydratase (NCBI) [Escherichia coli K12]	VIMSS17824	4.89	9.31	4.66	18.58	18.64	17.4
IlvB	acetolactate synthase large subunit (NCBI) [Escherichia coli K12]	VIMSS17731	11	11.97	6.99	16.05	12.71	16.58

IlvC	ketol-acid reductoisomerase (NCBI) [Escherichia coli K12]	VIMSS17826	41.54	63.85	53.55	54.05	55.08	60.5
IlvD	dihydroxy-acid dehydratase (NCBI) [Escherichia coli K12]	VIMSS1937120	39.1	43.9	38.42	69.25	65.25	65.47
IlvE	branched-chain amino acid aminotransferase (NCBI) [Escherichia coli K12]	VIMSS1937119	11	11.97	12.81	18.58	20.34	17.4
IlvH	acetolactate synthase small subunit (NCBI) [Escherichia coli K12]	VIMSS14224	3.67	2.66	4.66	1.69	3.39	2.49
IlvI	acetolactate synthase III large subunit (NCBI) [Escherichia coli K12]	VIMSS1936210	1.22	3.99	2.33	5.07	4.24	4.97
IlvM	acetolactate synthase II, small subunit (NCBI) [Escherichia coli K12]	VIMSS17821	0	0	0	2.53	2.54	2.49
Imp	organic solvent tolerance protein precursor (NCBI) [Escherichia coli K12]	VIMSS14200	1.22	1.33	1.16	1.69	0	2.49
InfA	translation initiation factor IF-1 (NCBI) [Escherichia coli K12]	VIMSS15008	6.11	3.99	5.82	3.38	4.24	3.32
InfB	translation initiation factor IF-2 (NCBI) [Escherichia coli K12]	VIMSS17241	54.98	34.58	46.57	33.78	39.83	37.29
InfC	translation initiation factor IF-3 (NCBI) [Escherichia coli K12]	VIMSS15837	30.54	19.95	30.27	21.96	16.1	19.89
IscA	iron-sulfur cluster assembly protein (NCBI) [Escherichia coli K12]	VIMSS16627	2.44	0	3.49	1.69	1.69	0.83
IscR	DNA-binding transcriptional repressor (NCBI) [Escherichia coli K12]	VIMSS16630	4.89	5.32	2.33	3.38	6.78	4.97
IscS	cysteine desulfurase (NCBI) [Escherichia coli K12]	VIMSS1936785	21.99	17.29	22.12	21.11	23.73	26.52
IscU	scaffold protein (NCBI) [Escherichia coli K12]	VIMSS16628	8.55	9.31	8.15	4.22	5.93	7.46
IspB	octaprenyl diphosphate synthase (NCBI) [Escherichia coli K12]	VIMSS17258	1.22	2.66	3.49	2.53	2.54	1.66
IspE	4-diphosphocytidyl-2-C-methyl-D-erythritol kinase (NCBI) [Escherichia coli K12]	VIMSS15330	0	1.33	1.16	0.84	1.69	1.66
IspF	2-C-methyl-D-erythritol 2,4-cyclodiphosphate synthase (NCBI) [Escherichia coli K12]	VIMSS16830	1.22	0	1.16	0.84	2.54	2.49
IspG	4-hydroxy-3-methylbut-2-en-1-yl diphosphate synthase (NCBI) [Escherichia coli K12]	VIMSS16614	12.22	11.97	13.97	8.45	9.32	9.12
Ivy	inhibitor of vertebrate C-lysozyme (NCBI) [Escherichia coli K12]	VIMSS14359	2.44	2.66	0	0.84	1.69	0.83
KatG	catalase/hydroperoxidase HPI(I) (NCBI) [Escherichia coli K12]	VIMSS17981	25.66	26.6	27.94	21.96	22.88	19.06
Kbl	2-amino-3-ketobutyrate coenzyme A ligase (NCBI) [Escherichia coli K12]	VIMSS17678	4.89	5.32	3.49	11.82	9.32	8.29
KdgR	predicted DNA-binding transcriptional regulator (NCBI) [Escherichia coli K12]	VIMSS15945	0	1.33	2.33	3.38	2.54	3.32
KdsA	2-dehydro-3-deoxyphosphooctonate aldolase (NCBI) [Escherichia coli K12]	VIMSS15337	11	6.65	11.64	17.74	16.95	14.92
KdsB	3-deoxy-manno-octulosonate cytidylyltransferase (NCBI) [Escherichia coli K12]	VIMSS15042	2.44	1.33	2.33	1.69	1.69	3.32
KdsD	D-arabinose 5-phosphate isomerase (NCBI) [Escherichia coli K12]	VIMSS17268	0	0	0	1.69	0.85	1.66
KefG	glutathione-regulated potassium-efflux system ancillary protein (NCBI) [Escherichia coli K12]	VIMSS17414	2.44	0	0	1.69	2.54	2.49
KsgA	dimethyladenosine transferase (NCBI) [Escherichia coli K12]	VIMSS14197	0	0	1.16	0	1.69	0.83
LacI	transcriptional repressor of the lac operon (VIMSS) [Escherichia coli K12]	VIMSS14483	37.87	46.56	31.43	27.02	27.12	21.55
LdcA	L,D-carboxypeptidase A (NCBI) [Escherichia coli K12]	VIMSS15314	2.44	1.33	1.16	1.69	3.39	1.66
LdhA	D-lactate dehydrogenase (NCBI) [Escherichia coli K12]	VIMSS15502	1.22	2.66	1.16	4.22	4.24	7.46
LepA	GTP-binding protein LepA (NCBI) [Escherichia coli K12]	VIMSS16668	6.11	5.32	4.66	6.76	3.39	6.63
LepB	leader peptidase (signal peptidase I) (NCBI) [Escherichia coli K12]	VIMSS16667	2.44	0	0	0.84	1.69	1.66
LeuA	2-isopropylmalate synthase (NCBI) [Escherichia coli K12]	VIMSS14220	11	9.31	11.64	14.36	22.88	20.72
LeuB	3-isopropylmalate dehydrogenase (VIMSS) [Escherichia coli K12]	VIMSS14219	30.54	34.58	32.6	46.45	43.22	38.95
LeuC	isopropylmalate isomerase large subunit (NCBI) [Escherichia coli K12]	VIMSS14218	28.1	30.59	27.94	26.18	28.81	31.49
LeuD	isopropylmalate isomerase small subunit (NCBI) [Escherichia coli K12]	VIMSS14217	13.44	13.3	12.81	18.58	17.8	17.4
LeuS	leucyl-tRNA synthetase (NCBI) [Escherichia coli K12]	VIMSS14779	17.1	13.3	20.96	20.27	14.41	16.58
LexA	LexA repressor (NCBI) [Escherichia coli K12]	VIMSS18071	1.22	0	1.16	0.84	3.39	4.97
LigA	NAD-dependent DNA ligase LigA (NCBI) [Escherichia coli K12]	VIMSS16510	0	1.33	0	0	0	1.66
LipA	lipoyl synthase (NCBI) [Escherichia coli K12]	VIMSS14765	3.67	2.66	0	1.69	1.69	2.49
LipB	protein of lipoate biosynthesis (VIMSS) [Escherichia coli K12]	VIMSS14767	0	1.33	2.33	1.69	1.69	0.83
LivJ	leucine/isoleucine/valine transporter subunit (NCBI) [Escherichia coli K12]	VIMSS1937025	15.88	9.31	17.46	13.51	10.17	12.43
LivK	leucine transporter subunit (NCBI) [Escherichia coli K12]	VIMSS17519	4.89	1.33	4.66	4.22	5.08	3.32
LolA	outer-membrane lipoprotein carrier protein precursor (NCBI) [Escherichia coli K12]	VIMSS15015	2.44	2.66	3.49	1.69	2.54	2.49
LolC	outer membrane-specific lipoprotein transporter subunit (NCBI) [Escherichia coli K12]	VIMSS15238	0	0	0	1.69	0.85	0
LpcA	phosphoheptose isomerase (NCBI) [Escherichia coli K12]	VIMSS14361	8.55	5.32	8.15	7.6	5.08	5.8
Lpd	dihydrolipoamide dehydrogenase (NCBI) [Escherichia coli K12]	VIMSS14262	70.86	102.42	66.36	68.41	68.64	62.16
LplA	lipoate-protein ligase A (NCBI) [Escherichia coli K12]	VIMSS18407	1.22	2.66	1.16	0	0	0
Lpp	murein lipoprotein (NCBI) [Escherichia coli K12]	VIMSS15796	3.67	2.66	3.49	2.53	2.54	0.83
LpxC	UDP-3-O-[3-hydroxymyristoyl] N-acetylglucosamine deacetylase (NCBI) [Escherichia coli K12]	VIMSS14242	0	0	0	3.38	0.85	1.66
LpxM	lipid A biosynthesis (KDO)2-(lauroyl)-lipid IVA acyltransferase (NCBI) [Escherichia coli K12]	VIMSS15973	0	0	1.16	0.84	1.69	0.83
Lrp	DNA-binding transcriptional dual regulator, leucine-binding (NCBI) [Escherichia coli K12]	VIMSS15013	4.89	9.31	8.15	5.91	3.39	5.8
LtaE	L-allo-threonine aldolase, PLP-dependent (NCBI) [Escherichia coli K12]	VIMSS14995	4.89	3.99	4.66	2.53	3.39	4.14
LuxS	S-ribosylhomocysteinase (NCBI) [Escherichia coli K12]	VIMSS16776	11	9.31	5.82	6.76	5.93	9.12
LysA	diaminopimelate decarboxylase, PLP-binding (NCBI) [Escherichia coli K12]	VIMSS16919	1.22	0	2.33	1.69	2.54	2.49
LysC	aspartate kinase III (NCBI) [Escherichia coli K12]	VIMSS18052	41.54	26.6	39.58	31.25	32.2	29.84
LysS	lysine tRNA synthetase, constitutive (NCBI) [Escherichia coli K12]	VIMSS16970	13.44	19.95	12.81	15.2	15.25	16.58
MaeB	malic enzyme (NCBI) [Escherichia coli K12]	VIMSS16562	9.77	9.31	5.82	9.29	6.78	6.63
ManA	mannose-6-phosphate isomerase (VIMSS) [Escherichia coli K12]	VIMSS15734	2.44	5.32	2.33	3.38	3.39	4.14
ManX	fused mannose-specific PTS enzymes: IIA component/IIB component (NCBI) [Escherichia coli K12]	VIMSS15935	18.33	17.29	16.3	13.51	15.25	9.12
ManZ	PTS enzyme IID, mannose-specific (VIMSS) [Escherichia coli K12]	VIMSS15937	7.33	7.98	3.49	4.22	2.54	3.32
Map	methionine aminopeptidase (NCBI) [Escherichia coli K12]	VIMSS14314	1.22	2.66	1.16	4.22	3.39	4.14

Mdh	malate dehydrogenase (NCBI) [Escherichia coli K12]	VIMSS17307	83.08	73.16	72.18	52.36	65.25	65.47
MdoG	glucan biosynthesis protein, periplasmic (NCBI) [Escherichia coli K12]	VIMSS15170	3.67	2.66	4.66	3.38	3.39	4.14
MdoH	glucosyltransferase MdoH (NCBI) [Escherichia coli K12]	VIMSS15171	1.22	0	0	0.84	2.54	2.49
MenB	naphthoate synthase (NCBI) [Escherichia coli K12]	VIMSS16369	2.44	2.66	2.33	4.22	1.69	3.32
MetA	homoserine O-succinyltransferase (NCBI) [Escherichia coli K12]	VIMSS18041	4.89	0	1.16	4.22	4.24	2.49
MetB	cystathionine gamma-synthase (NCBI) [Escherichia coli K12]	VIMSS17978	1.22	0	0	4.22	3.39	2.49
MetC	cystathionine beta-lyase (NCBI) [Escherichia coli K12]	VIMSS17085	0	1.33	1.16	4.22	2.54	4.14
MetE	5-methyltetrahydropteroyltriglutamate-- homocysteine methyltransferase (NCBI) [Escherichia coli K12]	VIMSS17877	103.85	83.8	108.27	94.59	99.15	88.68
MetF	5,10-methylenetetrahydrofolate reductase (NCBI) [Escherichia coli K12]	VIMSS17980	8.55	6.65	5.82	14.36	13.56	11.6
MetG	methionyl-tRNA synthetase (NCBI) [Escherichia coli K12]	VIMSS16221	15.88	14.63	17.46	25.34	20.34	22.38
MetH	B12-dependent methionine synthase (NCBI) [Escherichia coli K12]	VIMSS18047	21.99	35.91	32.6	18.58	24.58	21.55
MetK	S-adenosylmethionine synthetase (NCBI) [Escherichia coli K12]	VIMSS17021	20.77	27.93	31.43	25.34	25.42	19.06
MetL	bifunctional aspartate kinase II/homoserine dehydrogenase II (NCBI) [Escherichia coli K12]	VIMSS17979	6.11	2.66	3.49	6.76	8.47	6.63
MetN	DL-methionine transporter subunit (NCBI) [Escherichia coli K12]	VIMSS14345	3.67	1.33	2.33	1.69	1.69	0.83
MetQ	DL-methionine transporter subunit (NCBI) [Escherichia coli K12]	VIMSS14343	30.54	43.9	30.27	33.78	33.9	29.84
Mfd	transcription-repair coupling factor (NCBI) [Escherichia coli K12]	VIMSS15236	0	0	0	1.69	0.85	1.66
MgsA	methylglyoxal synthase (VIMSS) [Escherichia coli K12]	VIMSS15087	1.22	2.66	4.66	3.38	0.85	1.66
MinD	membrane ATPase of the MinC-MinD-MinE system (NCBI) [Escherichia coli K12]	VIMSS15297	24.44	23.94	18.63	11.82	12.71	12.43
MinE	cell division topological specificity factor MinE (NCBI) [Escherichia coli K12]	VIMSS15296	4.89	6.65	4.66	1.69	3.39	4.14
MntR	DNA-binding transcriptional regulator of mntH (NCBI) [Escherichia coli K12]	VIMSS14942	0	0	0	0.84	1.69	0.83
MoaB	molybdopterin biosynthesis protein B (NCBI) [Escherichia coli K12]	VIMSS14907	3.67	11.97	0	5.91	5.08	4.14
MoeA	molybdopterin biosynthesis protein (NCBI) [Escherichia coli K12]	VIMSS14952	6.11	3.99	5.82	10.13	6.78	9.12
Mog	molybdenum cofactor biosynthesis protein (NCBI) [Escherichia coli K12]	VIMSS14154	0	0	0	2.53	2.54	1.66
Mpl	UDP-N-acetylmuramate:L-alanyl-gamma-D-glutamyl- meso-diaminopimelate ligase (NCBI) [Escherichia coli K12]	VIMSS18258	0	1.33	2.33	0.84	1.69	1.66
MraW	S-adenosyl-methyltransferase (NCBI) [Escherichia coli K12]	VIMSS14228	8.55	14.63	11.64	5.91	8.47	6.63
MrcB	penicillin-binding protein 1b (NCBI) [Escherichia coli K12]	VIMSS14295	1.22	2.66	0	4.22	3.39	1.66
MreB	regulator of ftsI, penicillin binding protein 3, septation function (VIMSS) [Escherichia coli K12]	VIMSS17321	8.55	5.32	3.49	5.07	5.93	6.63
Mrp	putative ATPase (VIMSS) [Escherichia coli K12]	VIMSS16220	4.89	6.65	2.33	2.53	2.54	0.83
MsbA	fused lipid transporter subunits of ABC superfamily: membrane component/ATP-binding component (NCBI) [Escherichia coli K12]	VIMSS15038	0	0	0	2.53	2.54	1.66
MscS	mechanosensitive channel (NCBI) [Escherichia coli K12]	VIMSS17003	11	9.31	9.31	6.76	7.63	4.14
MsrA	methionine sulfoxide reductase A (NCBI) [Escherichia coli K12]	VIMSS18244	3.67	3.99	4.66	3.38	4.24	4.97
MtlA	fused mannitol-specific PTS enzymes: IIA components/IIB components/IIC components (NCBI) [Escherichia coli K12]	VIMSS17660	1.22	0	0	3.38	1.69	0.83
MtlD	mannitol-1-phosphate 5-dehydrogenase (NCBI) [Escherichia coli K12]	VIMSS17661	0	0	1.16	2.53	1.69	1.66
MukB	cell division protein MukB (NCBI) [Escherichia coli K12]	VIMSS15048	0	0	0	2.53	0.85	0.83
MukE	condesin subunit E (NCBI) [Escherichia coli K12]	VIMSS15047	0	0	0	0	1.69	1.66
MurA	UDP-N-acetylglucosamine 1-carboxyvinyltransferase (NCBI) [Escherichia coli K12]	VIMSS17260	7.33	6.65	6.99	6.76	8.47	6.63
MurC	UDP-N-acetylmuramate--L-alanine ligase (NCBI) [Escherichia coli K12]	VIMSS14237	1.22	2.66	1.16	2.53	3.39	0.83
MurD	UDP-N-acetylmuramoyl-L-alanyl-D-glutamatesynthetase (NCBI) [Escherichia coli K12]	VIMSS14234	3.67	3.99	2.33	1.69	2.54	1.66
MurE	UDP-N-acetylmuramoylalanyl-D-glutamate--2, 6-diaminopimelate ligase (NCBI) [Escherichia coli K12]	VIMSS14231	3.67	3.99	2.33	7.6	5.08	5.8
MurF	UDP-N-acetylmuramoyl-tripeptide:D-alanyl-D- alanine ligase (NCBI) [Escherichia coli K12]	VIMSS14232	0	0	1.16	1.69	1.69	1.66
MurG	N-acetylglucosaminyl transferase (NCBI) [Escherichia coli K12]	VIMSS14236	2.44	2.66	3.49	4.22	2.54	2.49
NadE	NAD synthetase (NCBI) [Escherichia coli K12]	VIMSS15858	7.33	6.65	6.99	10.98	12.71	13.26
NagA	N-acetylglucosamine-6-phosphate deacetylase (NCBI) [Escherichia coli K12]	VIMSS14807	1.22	0	0	3.38	1.69	1.66
NagD	UMP phosphatase (NCBI) [Escherichia coli K12]	VIMSS14805	1.22	2.66	3.49	4.22	3.39	4.14
NarL	DNA-binding response regulator in two-component regulatory system with NarX (or NarQ) (NCBI) [Escherichia coli K12]	VIMSS15343	8.55	7.98	8.15	10.13	12.71	14.09
NarP	DNA-binding response regulator in two-component regulatory system with NarQ or NarX (NCBI) [Escherichia coli K12]	VIMSS16301	0	0	0	2.53	1.69	1.66
Ndh	respiratory NADH dehydrogenase 2/cupric reductase (NCBI) [Escherichia coli K12]	VIMSS15231	2.44	3.99	2.33	5.07	7.63	4.97
Ndk	nucleoside diphosphate kinase (NCBI) [Escherichia coli K12]	VIMSS16617	11	7.98	15.13	20.27	21.19	20.72
NemA	N-ethylmaleimide reductase, FMN-linked (NCBI) [Escherichia coli K12]	VIMSS15771	20.77	18.62	17.46	18.58	18.64	19.89
NfnB	dihydropteridine reductase, NAD(P)H-dependent, oxygen-insensitive (NCBI) [Escherichia coli K12]	VIMSS14714	4.89	5.32	2.33	3.38	4.24	2.49
NfsA	nitroreductase A, NADPH-dependent, FMN-dependent (NCBI) [Escherichia coli K12]	VIMSS14976	1.22	1.33	0	2.53	3.39	2.49
NlpA	cytoplasmic membrane lipoprotein-28 (NCBI) [Escherichia coli K12]	VIMSS17721	4.89	6.65	6.99	5.07	1.69	5.8
NlpB	lipoprotein-34 (VIMSS) [Escherichia coli K12]	VIMSS16576	8.55	11.97	9.31	9.29	5.08	7.46
NlpD	predicted outer membrane lipoprotein (NCBI) [Escherichia coli K12]	VIMSS16826	3.67	2.66	1.16	0	0	0
NrdA	ribonucleotide-diphosphate reductase alpha subunit (NCBI) [Escherichia coli K12]	VIMSS16341	9.77	10.64	5.82	12.67	11.86	10.77
NrdB	ribonucleotide-diphosphate reductase beta subunit (NCBI) [Escherichia coli K12]	VIMSS16342	4.89	2.66	4.66	5.91	4.24	5.8
NudE	ADP-ribose diphosphatase (NCBI) [Escherichia coli K12]	VIMSS17460	7.33	7.98	5.82	7.6	5.08	7.46
NuoC	NADH:ubiquinone oxidoreductase, chain C,D (NCBI) [Escherichia coli K12]	VIMSS16393	8.55	9.31	8.15	8.45	11.86	10.77
NuoF	NADH:ubiquinone oxidoreductase, chain F (NCBI) [Escherichia coli K12]	VIMSS16391	2.44	0	2.33	2.53	2.54	4.14
NuoG	NADH dehydrogenase I chain G (VIMSS) [Escherichia coli K12]	VIMSS16390	8.55	11.97	8.15	18.58	18.64	16.58
NusA	transcription elongation factor NusA (NCBI) [Escherichia coli K12]	VIMSS17242	18.33	15.96	18.63	21.11	18.64	24.03
NusB	transcription antitermination protein NusB (NCBI) [Escherichia coli K12]	VIMSS14554	6.11	3.99	4.66	4.22	3.39	2.49

NusG	transcription antitermination protein NusG (NCBI) [Escherichia coli K12]	VIMSS18013	13.44	7.98	13.97	10.98	11.86	13.26
ObgE	GTPase involved in cell partioning and DNA repair (NCBI) [Escherichia coli K12]	VIMSS17254	2.44	2.66	3.49	0.84	5.08	3.32
OmpA	outer membrane protein A (3a;II*;G;d) (NCBI) [Escherichia coli K12]	VIMSS15081	172.27	203.51	173.46	51.52	68.64	72.93
OmpF	outer membrane porin 1a (Ia;b;F) (NCBI) [Escherichia coli K12]	VIMSS15053	4.89	3.99	5.82	5.07	5.08	3.32
OmpR	osmolarity response regulator (NCBI) [Escherichia coli K12]	VIMSS17468	4.89	3.99	4.66	3.38	5.93	7.46
OppA	oligopeptide transporter subunit (NCBI) [Escherichia coli K12]	VIMSS15363	32.99	34.58	25.61	34.63	31.35	29.01
OppD	oligopeptide transporter ATP-binding component (NCBI) [Escherichia coli K12]	VIMSS1936449	0	0	0	0.84	0	1.66
OppF	oligopeptide transporter subunit (NCBI) [Escherichia coli K12]	VIMSS15367	0	0	0	1.69	2.54	1.66
OsmC	osmotically inducible, stress-inducible membrane protein (NCBI) [Escherichia coli K12]	VIMSS15603	3.67	3.99	3.49	0	0	0
OxyR	DNA-binding transcriptional dual regulator (NCBI) [Escherichia coli K12]	VIMSS18000	6.11	1.33	4.66	4.22	4.24	4.97
Pal	peptidoglycan-associated outer membrane lipoprotein (NCBI) [Escherichia coli K12]	VIMSS14873	2.44	2.66	1.16	0	0.85	0
PanB	3-methyl-2-oxobutanoate hydroxymethyltransferase (NCBI) [Escherichia coli K12]	VIMSS14280	4.89	5.32	4.66	4.22	5.08	4.97
PanC	pantoate--beta-alanine ligase (NCBI) [Escherichia coli K12]	VIMSS14279	7.33	3.99	8.15	7.6	8.47	9.12
Pck	phosphoenolpyruvate carboxykinase (NCBI) [Escherichia coli K12]	VIMSS17466	18.33	27.93	17.46	10.13	7.63	9.12
PdhR	transcriptional regulator of pyruvate dehydrogenase complex (NCBI) [Escherichia coli K12]	VIMSS14259	0	2.66	0	0	1.69	0
PdxB	erythronate-4-phosphate dehydrogenase (NCBI) [Escherichia coli K12]	VIMSS16427	6.11	6.65	1.16	6.76	7.63	5.8
PdxH	pyridoxamine 5'-phosphate oxidase (NCBI) [Escherichia coli K12]	VIMSS15759	2.44	2.66	3.49	4.22	6.78	4.14
PdxJ	pyridoxal phosphate biosynthetic protein (NCBI) [Escherichia coli K12]	VIMSS16663	7.33	9.31	8.15	5.91	6.78	6.63
PdxK	pyridoxine kinase (NCBI) [Escherichia coli K12]	VIMSS16517	1.22	2.66	2.33	0.84	0.85	0.83
PepA	leucyl aminopeptidase (NCBI) [Escherichia coli K12]	VIMSS18285	6.11	3.99	4.66	5.07	7.63	4.14
PepB	putative peptidase (VIMSS) [Escherichia coli K12]	VIMSS16622	19.55	19.95	17.46	12.67	11.02	12.43
PepD	aminoacyl-histidine dipeptidase (peptidase D) (NCBI) [Escherichia coli K12]	VIMSS14376	8.55	5.32	9.31	7.6	10.17	11.6
PepN	aminopeptidase N (NCBI) [Escherichia coli K12]	VIMSS15056	7.33	6.65	6.99	13.51	11.86	16.58
PepP	proline aminopeptidase P II (NCBI) [Escherichia coli K12]	VIMSS16988	7.33	9.31	8.15	7.6	8.47	10.77
PepQ	proline dipeptidase (NCBI) [Escherichia coli K12]	VIMSS17893	14.66	7.98	13.97	14.36	10.17	9.95
PfkA	6-phosphofructokinase (NCBI) [Escherichia coli K12]	VIMSS17955	14.66	23.94	12.81	18.58	16.95	19.89
PfkB	6-phosphofructokinase II; suppressor of pfkA (VIMSS) [Escherichia coli K12]	VIMSS15841	6.11	7.98	6.99	5.91	7.63	7.46
PflA	pyruvate formate lyase activating enzyme 1 (NCBI) [Escherichia coli K12]	VIMSS15026	1.22	3.99	2.33	2.53	3.39	1.66
PflB	pyruvate formate lyase I (NCBI) [Escherichia coli K12]	VIMSS15027	18.33	18.62	16.3	16.05	13.56	17.4
Pfs	5'-methylthioadenosine/S-adenosylhomocysteine nucleosidase (NCBI) [Escherichia coli K12]	VIMSS14305	6.11	7.98	3.49	5.91	5.08	6.63
Pgi	glucose-6-phosphate isomerase (NCBI) [Escherichia coli K12]	VIMSS18053	13.44	10.64	12.81	10.98	16.1	11.6
Pgk	phosphoglycerate kinase (NCBI) [Escherichia coli K12]	VIMSS17005	78.19	82.47	79.17	75.16	77.12	68.79
Pgm	phosphoglucomutase (NCBI) [Escherichia coli K12]	VIMSS14818	23.21	21.28	13.97	13.51	15.25	13.26
PheS	phenylalanyl-tRNA synthetase alpha subunit (NCBI) [Escherichia coli K12]	VIMSS15833	12.22	15.96	11.64	13.51	14.41	12.43
PheT	phenylalanyl-tRNA synthetase beta subunit (NCBI) [Escherichia coli K12]	VIMSS15832	54.98	53.21	43.08	80.23	62.71	59.67
PhoP	DNA-binding response regulator in two-component regulatory system with PhoQ (NCBI) [Escherichia coli K12]	VIMSS15252	8.55	14.63	12.81	5.07	8.47	7.46
PhoU	negative regulator of PhoR/PhoB two-component regulatory (NCBI) [Escherichia coli K12]	VIMSS17784	0	0	0	1.69	0.85	0.83
PlsB	glycerol-3-phosphate acyltransferase (VIMSS) [Escherichia coli K12]	VIMSS18069	0	0	0	0	0.85	3.32
PmbA	predicted peptidase required for the maturation and secretion of the antibiotic peptide MccB17 (NCBI) [Escherichia coli K12]	VIMSS18260	1.22	3.99	3.49	3.38	6.78	4.14
PncB	nicotinate phosphoribosyltransferase (NCBI) [Escherichia coli K12]	VIMSS15055	0	1.33	2.33	1.69	0.85	1.66
Pnp	polynucleotide phosphorylase; cytidylate kinase activity (VIMSS) [Escherichia coli K12]	VIMSS17237	30.54	34.58	33.76	24.49	24.58	28.18
PntA	NAD(P) transhydrogenase subunit alpha (NCBI) [Escherichia coli K12]	VIMSS15724	20.77	35.91	31.43	23.65	23.73	18.23
PntB	pyridine nucleotide transhydrogenase (NCBI) [Escherichia coli K12]	VIMSS15723	9.77	11.97	10.48	5.07	5.08	7.46
PolA	DNA polymerase I (NCBI) [Escherichia coli K12]	VIMSS17904	2.44	1.33	1.16	5.07	6.78	6.63
PotA	putrescine/spermidine ABC transporter ATPase protein (NCBI) [Escherichia coli K12]	VIMSS15248	0	0	0	2.53	0	1.66
PotD	spermidine/putrescine ABC transporter periplasmic substrate-binding protein (NCBI) [Escherichia coli K12]	VIMSS15245	6.11	5.32	6.99	9.29	8.47	9.95
Ppa	inorganic pyrophosphatase (NCBI) [Escherichia coli K12]	VIMSS18251	1.22	2.66	1.16	1.69	2.54	2.49
Ppc	phosphoenolpyruvate carboxylase (NCBI) [Escherichia coli K12]	VIMSS17995	35.43	34.58	40.75	51.52	44.07	46.41
PpiA	peptidyl-prolyl cis-trans isomerase A (rotamase A) (NCBI) [Escherichia coli K12]	VIMSS17426	0	2.66	0	1.69	2.54	1.66
PpiB	peptidyl-prolyl cis-trans isomerase B (rotamase B) (NCBI) [Escherichia coli K12]	VIMSS14662	7.33	13.3	4.66	10.98	7.63	7.46
PpiD	peptidyl-prolyl cis-trans isomerase (rotamase D) (NCBI) [Escherichia coli K12]	VIMSS14579	20.77	17.29	17.46	16.89	14.41	10.77
Ppk	polyphosphate kinase (NCBI) [Escherichia coli K12]	VIMSS16600	1.22	5.32	3.49	3.38	5.93	1.66
Pps	phosphoenolpyruvate synthase (NCBI) [Escherichia coli K12]	VIMSS15821	20.77	23.94	25.61	20.27	18.64	22.38
PqiB	paraquat-inducible protein B (NCBI) [Escherichia coli K12]	VIMSS15075	0	1.33	1.16	3.38	1.69	1.66
Prc	carboxy-terminal protease for penicillin-binding protein 3 (NCBI) [Escherichia coli K12]	VIMSS15948	1.22	0	0	4.22	2.54	2.49
PREC	URSOR	TRYPSIN	2.44	2.66	4.66	5.07	4.24	2.49
PrfB	peptide chain release factor 2 (NCBI) [Escherichia coli K12]	VIMSS16971	0	5.32	5.82	2.53	0.85	2.49
PrfC	peptide chain release factor 3 (NCBI) [Escherichia coli K12]	VIMSS18396	3.67	2.66	3.49	5.91	2.54	1.66
PrkB	predicted phosphoribulokinase (NCBI) [Escherichia coli K12]	VIMSS17418	0	0	0	0	1.69	0
PrlC	oligopeptidase A (NCBI) [Escherichia coli K12]	VIMSS17559	2.44	2.66	3.49	3.38	5.08	4.97
ProA	gamma-glutamyl phosphate reductase (NCBI) [Escherichia coli K12]	VIMSS14382	9.77	6.65	8.15	8.45	6.78	5.8
ProB	gamma-glutamyl kinase (NCBI) [Escherichia coli K12]	VIMSS14381	3.67	3.99	4.66	4.22	4.24	4.14

ProQ	putative solute/DNA competence effector (NCBI) [Escherichia coli K12]	VIMSS1936604	4.89	3.99	3.49	1.69	3.39	3.32
ProS	prolyl-tRNA synthetase (NCBI) [Escherichia coli K12]	VIMSS14340	17.1	22.61	20.96	28.71	30.51	33.15
ProX	glycine betaine transporter subunit (NCBI) [Escherichia coli K12]	VIMSS16769	0	0	0	1.69	0	1.66
PrsA	ribose-phosphate pyrophosphokinase (NCBI) [Escherichia coli K12]	VIMSS15329	25.66	29.26	29.1	32.09	27.96	30.66
Psd	phosphatidylserine decarboxylase (NCBI) [Escherichia coli K12]	VIMSS18188	3.67	3.99	3.49	4.22	5.93	4.14
PspA	regulatory protein for phage-shock-protein operon (NCBI) [Escherichia coli K12]	VIMSS15424	20.77	33.25	19.79	10.13	8.47	7.46
PstS	phosphate transporter subunit (NCBI) [Escherichia coli K12]	VIMSS17788	1.22	1.33	1.16	2.53	1.69	2.49
Pta	phosphate acetyltransferase (NCBI) [Escherichia coli K12]	VIMSS16404	40.32	31.92	40.75	38.85	34.74	33.98
Pth	peptidyl-tRNA hydrolase (NCBI) [Escherichia coli K12]	VIMSS15326	1.22	1.33	1.16	3.38	2.54	2.49
PtsG	fused glucose-specific PTS enzymes: IIB component/IIC component (NCBI) [Escherichia coli K12]	VIMSS15223	1.22	1.33	0	2.53	4.24	1.66
PtsH	phosphohistidinoprotein-hexose phosphotransferase component of PTS system (Hpr) (NCBI) [Escherichia coli K12]	VIMSS16514	4.89	3.99	4.66	18.58	9.32	10.77
PtsI	PEP-protein phosphotransferase of PTS system (enzyme I) (NCBI) [Escherichia coli K12]	VIMSS16515	56.2	59.86	52.39	62.5	64.4	57.19
PtsN	sugar-specific enzyme IIA component of PTS (NCBI) [Escherichia coli K12]	VIMSS17275	3.67	6.65	2.33	4.22	5.08	4.14
PtsP	fused PTS enzyme: PEP-protein phosphotransferase (enzyme I)/GAF domain containing protein (NCBI) [Escherichia coli K12]	VIMSS16910	0	0	0	1.69	2.54	3.32
PurA	adenylosuccinate synthetase (NCBI) [Escherichia coli K12]	VIMSS18202	21.99	19.95	31.43	32.94	29.66	33.15
PurB	adenylosuccinate lyase (NCBI) [Escherichia coli K12]	VIMSS15253	11	5.32	12.81	9.29	13.56	10.77
PurC	phosphoribosylaminoimidazole-succinocarboxamidesynthase (NCBI) [Escherichia coli K12]	VIMSS16575	14.66	13.3	13.97	13.51	11.86	10.77
PurD	phosphoribosylamine--glycine ligase (NCBI) [Escherichia coli K12]	VIMSS18037	6.11	6.65	6.99	7.6	5.93	8.29
PurE	phosphoribosylaminoimidazole carboxylase catalytic subunit (NCBI) [Escherichia coli K12]	VIMSS14660	3.67	0	2.33	2.53	3.39	1.66
PurH	bifunctionalphosphoribosylaminoimidazolecarboxamide formyltransferase/IMP cyclohydrolase (NCBI) [Escherichia coli K12]	VIMSS18038	23.21	19.95	15.13	21.96	15.25	19.89
PurK	phosphoribosylaminoimidazole carboxylase (NCBI) [Escherichia coli K12]	VIMSS14659	0	0	1.16	0.84	2.54	1.66
PurL	phosphoribosylformylglycinamidine synthase (NCBI) [Escherichia coli K12]	VIMSS1936794	23.21	19.95	20.96	23.65	15.25	24.86
PurM	phosphoribosylaminoimidazole synthetase (NCBI) [Escherichia coli K12]	VIMSS16598	1.22	1.33	1.16	5.07	3.39	4.97
PurN	phosphoribosylglycinamide formyltransferase (NCBI) [Escherichia coli K12]	VIMSS16599	0	0	1.16	1.69	0.85	0.83
PurR	DNA-binding transcriptional repressor, hypoxanthine-binding (NCBI) [Escherichia coli K12]	VIMSS15779	7.33	2.66	6.99	5.07	6.78	3.32
PurT	phosphoribosylglycinamide formyltransferase 2 (NCBI) [Escherichia coli K12]	VIMSS15967	1.22	9.31	6.99	6.76	8.47	5.8
PurU	formyltetrahydrofolate deformylase (NCBI) [Escherichia coli K12]	VIMSS15352	4.89	1.33	4.66	4.22	5.08	7.46
PykA	pyruvate kinase (NCBI) [Escherichia coli K12]	VIMSS15972	18.33	23.94	17.46	30.4	22.88	24.03
PykF	pyruvate kinase (NCBI) [Escherichia coli K12]	VIMSS15795	20.77	23.94	19.79	20.27	18.64	17.4
PyrC	dihydroorotase (NCBI) [Escherichia coli K12]	VIMSS15184	1.22	1.33	0	0.84	0	2.49
PyrE	orotate phosphoribosyltransferase (NCBI) [Escherichia coli K12]	VIMSS17703	1.22	0	0	0.84	1.69	1.66
PyrF	orotidine 5'-phosphate decarboxylase (NCBI) [Escherichia coli K12]	VIMSS15401	1.22	2.66	2.33	1.69	5.08	3.32
PyrG	CTP synthetase (NCBI) [Escherichia coli K12]	VIMSS16864	13.44	11.97	11.64	16.05	16.1	15.75
PyrH	uridylate kinase (NCBI) [Escherichia coli K12]	VIMSS14317	1.22	2.66	3.49	3.38	5.93	4.14
Qor	quinone oxidoreductase, NADPH-dependent (NCBI) [Escherichia coli K12]	VIMSS18079	2.44	1.33	0	1.69	0.85	0.83
QseB	DNA-binding response regulator in two-component regulatory system with QseC (NCBI) [Escherichia coli K12]	VIMSS17101	1.22	0	2.33	0.84	0.85	0.83
RbfA	ribosome-binding factor A (NCBI) [Escherichia coli K12]	VIMSS17240	4.89	3.99	6.99	7.6	5.93	5.8
RcsF	predicted outer membrane protein, signal (NCBI) [Escherichia coli K12]	VIMSS14342	3.67	2.66	2.33	1.69	1.69	1.66
RelA	(p)ppGpp synthetase I/GTP pyrophosphokinase (NCBI) [Escherichia coli K12]	VIMSS16868	0	0	1.16	1.69	0	1.66
RfaD	ADP-L-glycero-D-mannoheptose-6-epimerase, NAD(P)-binding (NCBI) [Escherichia coli K12]	VIMSS17680	8.55	11.97	8.15	15.2	13.56	16.58
RfaE	fused heptose 7-phosphate kinase/heptose 1-phosphate adenyltransferase (NCBI) [Escherichia coli K12]	VIMSS17128	8.55	10.64	9.31	10.98	10.17	13.26
RfbA	glucose-1-phosphate thymidylyltransferase (NCBI) [Escherichia coli K12]	VIMSS16147	2.44	1.33	1.16	2.53	1.69	4.14
RfbB	dTDP-glucose 4,6 dehydratase, NAD(P)-binding (NCBI) [Escherichia coli K12]	VIMSS16149	2.44	1.33	3.49	1.69	1.69	1.66
RfbD	dTDP-4-dehydrorhamnose reductase subunit, NAD(P)-binding, of dTDP-L-rhamnose synthase (NCBI) [Escherichia coli K12]	VIMSS16148	1.22	1.33	1.16	0.84	0.85	1.66
RffA	TDP-4-oxo-6-deoxy-D-glucose transaminase (NCBI) [Escherichia coli K12]	VIMSS17842	0	0	1.16	1.69	0.85	0.83
RFP		RFP	0	0	0	1.69	0	0
RhlB	ATP-dependent RNA helicase (NCBI) [Escherichia coli K12]	VIMSS17831	3.67	3.99	1.16	5.91	7.63	5.8
Rho	transcription termination factor Rho (NCBI) [Escherichia coli K12]	VIMSS17834	47.65	27.93	41.91	51.52	50	47.24
RibB	3,4-dihydroxy-2-butanone 4-phosphate synthase (NCBI) [Escherichia coli K12]	VIMSS17117	0	0	0	1.69	1.69	0
RibC	riboflavin synthase subunit alpha (NCBI) [Escherichia coli K12]	VIMSS15783	1.22	1.33	1.16	1.69	1.69	1.66
RibD	fused diaminohydroxyphosphoribosylaminopyrimidine deaminase and 5-amino-6-(5-phosphoribosylamino) uracil reductase (NCBI) [Escherichia co	VIMSS14552	0	1.33	2.33	0.84	2.54	1.66
RibE	riboflavin synthase subunit beta (NCBI) [Escherichia coli K12]	VIMSS14553	14.66	15.96	16.3	13.51	15.25	15.75
RlmB	23S rRNA (Gm2251)-methyltransferase (NCBI) [Escherichia coli K12]	VIMSS18205	3.67	2.66	2.33	2.53	5.93	4.97
RlpA	minor lipoprotein (NCBI) [Escherichia coli K12]	VIMSS14770	1.22	3.99	1.16	1.69	0.85	1.66
RlpB	minor lipoprotein (NCBI) [Escherichia coli K12]	VIMSS14778	2.44	1.33	2.33	1.69	2.54	2.49
RluD	23S rRNA pseudouridine synthase (NCBI) [Escherichia coli K12]	VIMSS16689	1.22	1.33	1.16	2.53	1.69	3.32
Rnb	exoribonuclease II (NCBI) [Escherichia coli K12]	VIMSS15406	1.22	0	2.33	1.69	5.08	4.14
Rnc	ribonuclease III (NCBI) [Escherichia coli K12]	VIMSS16666	1.22	1.33	1.16	1.69	1.69	1.66
Rne	fused ribonucleaseE: endoribonuclease/RNA-binding protein/RNA degradosome binding protein (NCBI) [Escherichia coli K12]	VIMSS15206	13.44	14.63	18.63	13.51	14.41	10.77
Rnt	ribonuclease T (NCBI) [Escherichia coli K12]	VIMSS15773	0	0	0	0.84	0.85	1.66
Rob	DNA-binding transcriptional activator (NCBI) [Escherichia coli K12]	VIMSS18417	3.67	2.66	4.66	2.53	1.69	1.66
Rpe	ribulose-phosphate 3-epimerase (NCBI) [Escherichia coli K12]	VIMSS17449	8.55	2.66	3.49	13.51	11.02	9.95

RpiA	ribose-5-phosphate isomerase A (NCBI) [Escherichia coli K12]	VIMSS16993	8.55	10.64	9.31	9.29	10.17	9.95
RplA	50S ribosomal protein L1 (NCBI) [Escherichia coli K12]	VIMSS18015	21.99	35.91	23.28	23.65	22.03	21.55
RplB	50S ribosomal protein L2 (NCBI) [Escherichia coli K12]	VIMSS17380	29.32	27.93	25.61	43.07	44.07	39.78
RplC	50S ribosomal protein L3 (NCBI) [Escherichia coli K12]	VIMSS17383	13.44	19.95	11.64	24.49	24.58	28.18
RplD	50S ribosomal protein L4 (NCBI) [Escherichia coli K12]	VIMSS17382	12.22	17.29	16.3	26.18	27.12	27.35
RplE	50S ribosomal protein L5 (NCBI) [Escherichia coli K12]	VIMSS17371	26.88	23.94	25.61	42.23	36.44	41.44
RplF	50S ribosomal protein L6 (NCBI) [Escherichia coli K12]	VIMSS17368	20.77	27.93	24.45	26.18	33.05	29.84
RplI	50S ribosomal protein L9 (NCBI) [Escherichia coli K12]	VIMSS18228	36.65	25.27	33.76	41.38	52.54	48.07
RplJ	50S ribosomal protein L10 (NCBI) [Escherichia coli K12]	VIMSS18016	25.66	23.94	26.78	22.8	31.35	33.15
RplK	50S ribosomal protein L11 (NCBI) [Escherichia coli K12]	VIMSS18014	9.77	15.96	12.81	8.45	9.32	9.12
RplL	50S ribosomal protein L7/L12 (NCBI) [Escherichia coli K12]	VIMSS18017	46.43	50.55	62.87	30.4	34.74	36.47
RplM	50S ribosomal protein L13 (NCBI) [Escherichia coli K12]	VIMSS17302	7.33	10.64	8.15	8.45	12.71	9.95
RplN	50S ribosomal protein L14 (NCBI) [Escherichia coli K12]	VIMSS17373	9.77	5.32	12.81	9.29	10.17	9.12
RplO	50S ribosomal protein L15 (NCBI) [Escherichia coli K12]	VIMSS17364	15.88	13.3	20.96	14.36	17.8	15.75
RplP	50S ribosomal protein L16 (NCBI) [Escherichia coli K12]	VIMSS17376	13.44	18.62	15.13	16.05	18.64	19.06
RplQ	50S ribosomal protein L17 (NCBI) [Escherichia coli K12]	VIMSS17357	13.44	14.63	17.46	18.58	16.95	19.89
RplR	50S ribosomal protein L18 (NCBI) [Escherichia coli K12]	VIMSS17367	6.11	7.98	2.33	1.69	3.39	2.49
RplS	50S ribosomal protein L19 (NCBI) [Escherichia coli K12]	VIMSS16701	4.89	7.98	3.49	10.13	14.41	10.77
RplT	50S ribosomal protein L20 (NCBI) [Escherichia coli K12]	VIMSS15835	1.22	0	2.33	7.6	7.63	9.12
RplU	50S ribosomal protein L21 (NCBI) [Escherichia coli K12]	VIMSS17257	4.89	3.99	5.82	5.07	7.63	8.29
RplV	50S ribosomal protein L22 (NCBI) [Escherichia coli K12]	VIMSS17378	15.88	9.31	13.97	26.18	28.81	27.35
RplX	50S ribosomal protein L24 (NCBI) [Escherichia coli K12]	VIMSS17372	6.11	0	5.82	7.6	11.02	11.6
RplY	50S ribosomal protein L25 (NCBI) [Escherichia coli K12]	VIMSS16294	17.1	14.63	15.13	21.96	25.42	29.84
RpmA	50S ribosomal protein L27 (NCBI) [Escherichia coli K12]	VIMSS17256	6.11	1.33	5.82	5.07	4.24	3.32
RpmC	50S ribosomal protein L29 (NCBI) [Escherichia coli K12]	VIMSS17375	14.66	19.95	11.64	15.2	16.95	13.26
RpmD	50S ribosomal protein L30 (NCBI) [Escherichia coli K12]	VIMSS17365	3.67	1.33	3.49	5.91	8.47	5.8
RpmF	50S ribosomal protein L32 (NCBI) [Escherichia coli K12]	VIMSS15211	1.22	1.33	1.16	4.22	5.08	4.14
RpmG	50S ribosomal protein L33 (NCBI) [Escherichia coli K12]	VIMSS17697	7.33	1.33	4.66	6.76	6.78	4.97
RpoA	DNA-directed RNA polymerase subunit alpha (NCBI) [Escherichia coli K12]	VIMSS17358	42.76	31.92	43.08	42.23	57.62	51.38
RpoB	DNA-directed RNA polymerase subunit beta (NCBI) [Escherichia coli K12]	VIMSS18018	73.31	61.19	78	78.54	72.03	77.9
RpoC	DNA-directed RNA polymerase subunit beta' (NCBI) [Escherichia coli K12]	VIMSS18019	65.97	57.2	67.52	74.32	72.03	73.76
RpoD	RNA polymerase sigma factor (NCBI) [Escherichia coli K12]	VIMSS17143	6.11	7.98	6.99	15.2	15.25	10.77
RpoZ	DNA-directed RNA polymerase subunit omega (NCBI) [Escherichia coli K12]	VIMSS17710	3.67	2.66	3.49	5.07	4.24	4.97
RpsA	30S ribosomal protein S1 (NCBI) [Escherichia coli K12]	VIMSS15035	42.76	41.23	53.55	58.27	44.07	56.36
RpsB	30S ribosomal protein S2 (NCBI) [Escherichia coli K12]	VIMSS14315	21.99	23.94	27.94	21.11	21.19	24.86
RpsC	30S ribosomal protein S3 (NCBI) [Escherichia coli K12]	VIMSS17377	28.1	29.26	32.6	32.09	26.27	29.84
RpsD	30S ribosomal protein S4 (NCBI) [Escherichia coli K12]	VIMSS17359	26.88	35.91	24.45	26.18	30.51	30.66
RpsE	30S ribosomal protein S5 (NCBI) [Escherichia coli K12]	VIMSS17366	13.44	27.93	15.13	16.89	18.64	17.4
RpsF	30S ribosomal protein S6 (NCBI) [Escherichia coli K12]	VIMSS18225	12.22	15.96	19.79	12.67	10.17	12.43
RpsG	30S ribosomal protein S7 (NCBI) [Escherichia coli K12]	VIMSS17404	29.32	33.25	30.27	31.25	31.35	26.52
RpsH	30S ribosomal protein S8 (NCBI) [Escherichia coli K12]	VIMSS17369	2.44	3.99	3.49	9.29	7.63	4.14
RpsI	30S ribosomal protein S9 (NCBI) [Escherichia coli K12]	VIMSS17301	7.33	3.99	6.99	7.6	5.08	4.14
RpsJ	30S ribosomal protein S10 (NCBI) [Escherichia coli K12]	VIMSS17384	17.1	27.93	24.45	32.09	22.03	22.38
RpsK	30S ribosomal protein S11 (NCBI) [Escherichia coli K12]	VIMSS17360	17.1	7.98	16.3	16.89	21.19	18.23
RpsL	30S ribosomal protein S12 (NCBI) [Escherichia coli K12]	VIMSS17405	0	0	0	1.69	1.69	4.14
RpsM	30S ribosomal protein S13 (NCBI) [Escherichia coli K12]	VIMSS17361	3.67	6.65	6.99	13.51	11.02	9.12
RpsO	30S ribosomal protein S15 (NCBI) [Escherichia coli K12]	VIMSS17238	0	1.33	1.16	4.22	0.85	1.66
RpsP	30S ribosomal protein S16 (NCBI) [Escherichia coli K12]	VIMSS16704	12.22	5.32	9.31	11.82	11.86	9.95
RpsR	30S ribosomal protein S18 (NCBI) [Escherichia coli K12]	VIMSS18227	12.22	7.98	8.15	9.29	10.17	9.95
RpsS	30S ribosomal protein S19 (NCBI) [Escherichia coli K12]	VIMSS17379	3.67	1.33	3.49	5.07	8.47	6.63
RpsT	30S ribosomal protein S20 (NCBI) [Escherichia coli K12]	VIMSS14168	9.77	5.32	10.48	9.29	8.47	8.29
RpsU	30S ribosomal protein S21 (NCBI) [Escherichia coli K12]	VIMSS17141	2.44	0	2.33	0.84	2.54	2.49
RraA	ribonuclease activity regulator protein RraA (NCBI) [Escherichia coli K12]	VIMSS17968	14.66	7.98	11.64	3.38	4.24	5.8
RrmJ	23S rRNA methyltransferase (NCBI) [Escherichia coli K12]	VIMSS17250	2.44	2.66	1.16	0.84	0.85	0.83
Rsd	stationary phase protein, binds sigma 70 RNA polymerase subunit (NCBI) [Escherichia coli K12]	VIMSS18027	0	1.33	0	0.84	1.69	0.83
RseA	anti-sigma factor (NCBI) [Escherichia coli K12]	VIMSS16671	1.22	2.66	1.16	0	0	0.83
RseB	periplasmic negative regulator of sigmaE (NCBI) [Escherichia coli K12]	VIMSS16670	2.44	1.33	1.16	1.69	1.69	4.14
RstA	DNA-binding response regulator in two-component regulatory system with RstB (NCBI) [Escherichia coli K12]	VIMSS15729	0	2.66	3.49	0.84	0	1.66
RuvA	Holliday junction DNA helicase motor protein (NCBI) [Escherichia coli K12]	VIMSS15979	0	0	0	1.69	0.85	0.83
RuvB	Holliday junction DNA helicase B (NCBI) [Escherichia coli K12]	VIMSS15978	0	0	0	0.84	1.69	0.83
SdhA	succinate dehydrogenase flavoprotein subunit (NCBI) [Escherichia coli K12]	VIMSS14855	39.1	39.9	29.1	24.49	22.88	24.03
SdhB	succinate dehydrogenase, FeS subunit (NCBI) [Escherichia coli K12]	VIMSS14856	17.1	21.28	16.3	10.13	9.32	8.29

SecA	translocase (NCBI) [Escherichia coli K12]	VIMSS14244	12.22	9.31	11.64	21.11	23.73	19.89
SecB	export protein SecB (NCBI) [Escherichia coli K12]	VIMSS17670	15.88	14.63	18.63	10.13	7.63	5.8
SecD	protein export protein SecD (NCBI) [Escherichia coli K12]	VIMSS14546	15.88	9.31	11.64	10.13	13.56	10.77
SecY	protein translocase subunit SecY (NCBI) [Escherichia coli K12]	VIMSS17363	1.22	0	0	2.53	0	0
SelB	selenocysteinyl-tRNA-specific translation factor (NCBI) [Escherichia coli K12]	VIMSS17651	1.22	0	1.16	0.84	2.54	0.83
SelD	selenophosphate synthetase (NCBI) [Escherichia coli K12]	VIMSS15882	6.11	3.99	11.64	3.38	2.54	4.14
SeqA	regulatory protein for replication initiation (NCBI) [Escherichia coli K12]	VIMSS14817	3.67	3.99	1.16	2.53	2.54	3.32
SerA	D-3-phosphoglycerate dehydrogenase (NCBI) [Escherichia coli K12]	VIMSS16992	37.87	43.9	39.58	30.4	29.66	31.49
SerB	3-phosphoserine phosphatase (NCBI) [Escherichia coli K12]	VIMSS18409	0	0	0	1.69	2.54	1.66
SerC	phosphoserine aminotransferase (NCBI) [Escherichia coli K12]	VIMSS15031	40.32	35.91	43.08	41.38	29.66	31.49
SerS	seryl-tRNA synthetase (NCBI) [Escherichia coli K12]	VIMSS15017	21.99	13.3	22.12	19.42	18.64	18.23
SfcA	NAD-linked malate dehydrogenase (malic enzyme) (VIMSS) [Escherichia coli K12]	VIMSS15600	4.89	3.99	5.82	1.69	2.54	3.32
SlyA	transcriptional regulator for cryptic hemolysin (VIMSS) [Escherichia coli K12]	VIMSS15763	2.44	3.99	1.16	2.53	2.54	3.32
SlyB	outer membrane lipoprotein (NCBI) [Escherichia coli K12]	VIMSS1936555	1.22	1.33	1.16	1.69	2.54	4.14
SlyD	FKBP-type peptidyl prolyl cis-trans isomerase (rotamase) (NCBI) [Escherichia coli K12]	VIMSS17412	11	10.64	8.15	10.13	8.47	5.8
SmpA	small membrane protein A (VIMSS) [Escherichia coli K12]	VIMSS16711	2.44	2.66	2.33	2.53	0.85	2.49
SodA	superoxide dismutase, manganese (VIMSS) [Escherichia coli K12]	VIMSS17948	2.44	6.65	1.16	5.07	5.08	6.63
SodB	superoxide dismutase, Fe (NCBI) [Escherichia coli K12]	VIMSS15777	21.99	15.96	19.79	11.82	16.1	15.75
SohB	predicted inner membrane peptidase (NCBI) [Escherichia coli K12]	VIMSS15392	6.11	6.65	6.99	5.91	6.78	5.8
SolA	N-methyltryptophan oxidase, FAD-binding (NCBI) [Escherichia coli K12]	VIMSS15181	4.89	3.99	6.99	7.6	6.78	5.8
SpeA	arginine decarboxylase (NCBI) [Escherichia coli K12]	VIMSS17017	3.67	2.66	3.49	13.51	22.03	14.92
SpeB	agmatinase (NCBI) [Escherichia coli K12]	VIMSS17016	1.22	1.33	0	1.69	0.85	1.66
SpeC	ornithine decarboxylase isozyme (VIMSS) [Escherichia coli K12]	VIMSS17044	0	0	1.16	2.53	0	2.49
SpeD	S-adenosylmethionine decarboxylase proenzyme (NCBI) [Escherichia coli K12]	VIMSS14266	0	1.33	0	0.84	0.85	1.66
SpeE	spermidine synthase (NCBI) [Escherichia coli K12]	VIMSS14267	1.22	1.33	3.49	1.69	2.54	1.66
SpeG	spermidine N1-acetyltransferase (NCBI) [Escherichia coli K12]	VIMSS15705	1.22	1.33	3.49	0.84	0	0
SrmB	ATP-dependent RNA helicase (NCBI) [Escherichia coli K12]	VIMSS16675	3.67	3.99	1.16	5.07	6.78	6.63
Ssb	single-strand DNA-binding protein (NCBI) [Escherichia coli K12]	VIMSS18087	6.11	3.99	5.82	3.38	2.54	2.49
SseA	putative thiosulfate sulfurtransferase (VIMSS) [Escherichia coli K12]	VIMSS16620	6.11	3.99	6.99	5.07	3.39	4.97
SspA	stringent starvation protein A (NCBI) [Escherichia coli K12]	VIMSS17300	12.22	19.95	11.64	12.67	12.71	11.6
SspB	ClpXP protease specificity-enhancing factor (NCBI) [Escherichia coli K12]	VIMSS17299	1.22	1.33	1.16	1.69	1.69	1.66
StpA	DNA binding protein, nucleoid-associated (NCBI) [Escherichia coli K12]	VIMSS16759	8.55	6.65	10.48	15.2	14.41	10.77
SucA	alpha-ketoglutarate decarboxylase (NCBI) [Escherichia coli K12]	VIMSS14858	53.76	43.9	64.03	38	36.44	39.78
SucB	dihydrolipoamide acetyltransferase (NCBI) [Escherichia coli K12]	VIMSS14859	51.31	46.56	46.57	32.09	35.59	38.12
SucC	succinyl-CoA synthetase subunit beta (NCBI) [Escherichia coli K12]	VIMSS14860	53.76	71.83	53.55	48.14	43.22	40.61
SucD	succinyl-CoA synthetase subunit alpha (NCBI) [Escherichia coli K12]	VIMSS14861	36.65	25.27	33.76	19.42	19.49	17.4
SufI	repressor protein for FtsI (NCBI) [Escherichia coli K12]	VIMSS17093	0	0	0	1.69	1.69	0.83
SuhB	inositol monophosphatase (NCBI) [Escherichia coli K12]	VIMSS16632	1.22	1.33	0	4.22	5.08	3.32
SurA	peptidyl-prolyl cis-trans isomerase (PPIase) (NCBI) [Escherichia coli K12]	VIMSS14199	12.22	18.62	12.81	9.29	8.47	8.29
TalB	transaldolase B (NCBI) [Escherichia coli K12]	VIMSS14153	40.32	35.91	39.58	27.87	27.12	21.55
Tas	predicted oxidoreductase, NADP(H)-dependent aldo-keto reductase (NCBI) [Escherichia coli K12]	VIMSS16915	6.11	2.66	6.99	5.07	9.32	6.63
TauA	taurine transport system periplasmic protein (VIMSS) [Escherichia coli K12]	VIMSS14503	0	1.33	0	1.69	2.54	2.49
TauD	taurine dioxygenase (NCBI) [Escherichia coli K12]	VIMSS14506	2.44	1.33	2.33	2.53	3.39	4.14
Tdh	L-threonine 3-dehydrogenase (NCBI) [Escherichia coli K12]	VIMSS17677	4.89	6.65	4.66	4.22	6.78	3.32
TesB	acyl-CoA thioesterase II (NCBI) [Escherichia coli K12]	VIMSS14590	1.22	2.66	2.33	0.84	3.39	1.66
Tgt	queuine tRNA-ribosyltransferase (NCBI) [Escherichia coli K12]	VIMSS14544	0	0	0	1.69	0.85	0.83
ThiJ	4-methyl-5(beta-hydroxyethyl)-thiazole monophosphate synthesis (VIMSS) [Escherichia coli K12]	VIMSS14562	6.11	3.99	4.66	1.69	2.54	2.49
ThrA	bifunctional aspartokinase I/homeserine dehydrogenase I (NCBI) [Escherichia coli K12]	VIMSS14147	24.44	25.27	26.78	32.09	25.42	28.18
ThrC	threonine synthase (NCBI) [Escherichia coli K12]	VIMSS14149	24.44	14.63	20.96	21.11	24.58	24.86
ThrS	threonyl-tRNA synthetase (NCBI) [Escherichia coli K12]	VIMSS15838	24.44	23.94	26.78	19.42	15.25	20.72
ThyA	thymidylate synthase (NCBI) [Escherichia coli K12]	VIMSS16908	2.44	2.66	2.33	2.53	3.39	2.49
Tig	trigger factor (NCBI) [Escherichia coli K12]	VIMSS14574	46.43	58.53	43.08	55.74	52.54	62.16
TktA	transketolase 1, thiamin-binding (NCBI) [Escherichia coli K12]	VIMSS1936890	53.76	57.2	53.55	34.63	37.29	31.49
TldD	predicted peptidase (NCBI) [Escherichia coli K12]	VIMSS17315	2.44	2.66	1.16	2.53	1.69	1.66
Tmk	thymidylate kinase (NCBI) [Escherichia coli K12]	VIMSS15220	1.22	2.66	1.16	2.53	1.69	2.49
TolA	cell envelope integrity inner membrane protein TolA (NCBI) [Escherichia coli K12]	VIMSS14871	0	1.33	1.16	0.84	0.85	1.66
TolB	translocation protein TolB precursor (NCBI) [Escherichia coli K12]	VIMSS14872	9.77	15.96	9.31	5.91	7.63	7.46
TolC	outer membrane channel; specific tolerance to colicin E1; segregation of daughter chromosomes (VIMSS) [Escherichia coli K12]	VIMSS17111	11	10.64	12.81	3.38	4.24	3.32
TolQ	membrane spanning protein in TolA-TolQ-TolR complex (NCBI) [Escherichia coli K12]	VIMSS14869	1.22	1.33	1.16	1.69	3.39	0.83
TopA	DNA topoisomerase I (NCBI) [Escherichia coli K12]	VIMSS15394	7.33	3.99	3.49	2.53	1.69	1.66
TpiA	triosephosphate isomerase (NCBI) [Escherichia coli K12]	VIMSS17958	12.22	5.32	12.81	6.76	5.93	4.14
Tpx	thiol peroxidase (NCBI) [Escherichia coli K12]	VIMSS15444	24.44	29.26	24.45	21.11	20.34	17.4

TrmE	tRNA modification GTPase (NCBI) [Escherichia coli K12]	VIMSS17766	3.67	3.99	1.16	6.76	4.24	2.49
TrpA	tryptophan synthase subunit alpha (NCBI) [Escherichia coli K12]	VIMSS15380	26.88	23.94	31.43	32.09	23.73	19.89
TrpB	tryptophan synthase subunit beta (NCBI) [Escherichia coli K12]	VIMSS15381	20.77	17.29	18.63	17.74	16.1	13.26
TrpC	N-(5-phosphoribosyl)anthranilate isomerase and indole-3-glycerolphosphate synthetase (VIMSS) [Escherichia coli K12]	VIMSS15382	18.33	10.64	19.79	15.2	15.25	14.09
TrpD	bifunctional indole-3-glycerol-phosphate synthase/anthranilate phosphoribosyltransferase (NCBI) [Escherichia coli K12]	VIMSS15383	15.88	18.62	19.79	19.42	15.25	13.26
TrpE	anthranilate synthase component I (NCBI) [Escherichia coli K12]	VIMSS15384	8.55	9.31	11.64	10.98	10.17	13.26
TrpS	tryptophanyl-tRNA synthetase (NCBI) [Escherichia coli K12]	VIMSS17447	6.11	5.32	6.99	17.74	14.41	17.4
TruD	tRNA pseudouridine synthase D (NCBI) [Escherichia coli K12]	VIMSS16829	1.22	1.33	2.33	3.38	2.54	2.49
TrxA	thioredoxin 1 (VIMSS) [Escherichia coli K12]	VIMSS17832	12.22	9.31	9.31	14.36	13.56	11.6
TrxB	thioredoxin reductase, FAD/NAD(P)-binding (NCBI) [Escherichia coli K12]	VIMSS15012	29.32	26.6	20.96	21.11	22.88	17.4
TrxC	thioredoxin 2 (NCBI) [Escherichia coli K12]	VIMSS16681	2.44	3.99	1.16	0.84	1.69	1.66
Tsf	elongation factor Ts (NCBI) [Escherichia coli K12]	VIMSS14316	21.99	30.59	23.28	30.4	22.88	29.01
Ttk	putative transcriptional regulator (VIMSS) [Escherichia coli K12]	VIMSS17702	0	1.33	2.33	0	0	0
TufA	protein chain elongation factor EF-Tu (duplicate of tufB) (NCBI) [Escherichia coli K12]	VIMSS17402 (+	455.71	416.34	473.83	417.2	425.4	425.99
TyrA	fused chorismate mutase T/prephenate dehydrogenase (NCBI) [Escherichia coli K12]	VIMSS16695	64.75	55.87	60.54	67.56	67.79	65.47
TyrB	tyrosine aminotransferase, tyrosine-repressible, PLP-dependent (NCBI) [Escherichia coli K12]	VIMSS18082	19.55	17.29	16.3	15.2	16.1	14.09
TyrS	tyrosyl-tRNA synthetase (NCBI) [Escherichia coli K12]	VIMSS15758	23.21	30.59	16.3	17.74	11.86	20.72
UbiC	chorismate lyase (VIMSS) [Escherichia coli K12]	VIMSS18067	1.22	0	1.16	0	2.54	0.83
UbiD	3-octaprenyl-4-hydroxybenzoate decarboxylase (NCBI) [Escherichia coli K12]	VIMSS17889	1.22	2.66	2.33	1.69	1.69	1.66
UbiE	ubiquinone/menaquinone biosynthesis methyltransferase (NCBI) [Escherichia coli K12]	VIMSS1937152	4.89	6.65	4.66	3.38	4.24	3.32
UbiG	3-demethylubiquinone-9 3-methyltransferase (NCBI) [Escherichia coli K12]	VIMSS16339	1.22	2.66	1.16	2.53	1.69	2.49
UcpA	putative oxidoreductase (VIMSS) [Escherichia coli K12]	VIMSS16525	1.22	2.66	3.49	0	0	0
UdhA	putative oxidoreductase (VIMSS) [Escherichia coli K12]	VIMSS18001	14.66	10.64	10.48	11.82	8.47	10.77
Udk	uridine/cytidine kinase (VIMSS) [Escherichia coli K12]	VIMSS16174	0	0	1.16	1.69	0.85	0.83
Udp	uridine phosphorylase (NCBI) [Escherichia coli K12]	VIMSS17879	6.11	3.99	3.49	6.76	5.08	6.63
Ugd	UDP-glucose 6-dehydrogenase (NCBI) [Escherichia coli K12]	VIMSS16136	3.67	0	2.33	2.53	2.54	4.14
Upp	uracil phosphoribosyltransferase (VIMSS) [Escherichia coli K12]	VIMSS16597	8.55	6.65	10.48	9.29	9.32	10.77
Usg	hypothetical protein (NCBI) [Escherichia coli K12]	VIMSS16426	12.22	9.31	16.3	19.42	23.73	20.72
UshA	UDP-sugar hydrolase (NCBI) [Escherichia coli K12]	VIMSS14617	1.22	1.33	0	0.84	1.69	2.49
UspA	universal stress global response regulator (NCBI) [Escherichia coli K12]	VIMSS17556	51.31	26.6	51.22	21.11	22.88	23.21
UspE	stress-induced protein (NCBI) [Escherichia coli K12]	VIMSS15453	21.99	26.6	19.79	18.58	14.41	15.75
UspG	universal stress protein UP12 (NCBI) [Escherichia coli K12]	VIMSS14744	12.22	10.64	13.97	16.89	9.32	8.29
UvrB	excinuclease ABC subunit B (NCBI) [Escherichia coli K12]	VIMSS14904	0	0	0	3.38	5.08	0.83
VacB	putative enzyme (VIMSS) [Escherichia coli K12]	VIMSS18204	9.77	6.65	8.15	6.76	9.32	8.29
ValS	valyl-tRNA synthetase (NCBI) [Escherichia coli K12]	VIMSS18283	20.77	25.27	23.28	24.49	26.27	23.21
WzzB	regulator of length of O-antigen component of lipopolysaccharide chains (VIMSS) [Escherichia coli K12]	VIMSS16135	2.44	5.32	5.82	3.38	5.08	4.14
WzzE	putative transport protein (VIMSS) [Escherichia coli K12]	VIMSS17836	0	0	0	1.69	0.85	0.83
XthA	exonuclease III (NCBI) [Escherichia coli K12]	VIMSS15867	2.44	2.66	1.16	3.38	2.54	2.49
YaaA	hypothetical protein (NCBI) [Escherichia coli K12]	VIMSS14151	0	0	0	2.53	1.69	1.66
YadG	predicted transporter subunit: ATP-binding component of ABC superfamily (NCBI) [Escherichia coli K12]	VIMSS14273	0	1.33	0	2.53	3.39	3.32
YadR	hypothetical protein (NCBI) [Escherichia coli K12]	VIMSS14302	9.77	5.32	9.31	5.07	5.93	7.46
YaeH	hypothetical protein (NCBI) [Escherichia coli K12]	VIMSS14309	1.22	0	3.49	0.84	0.85	1.66
YaeO	orf, hypothetical protein (VIMSS) [Escherichia coli K12]	VIMSS14335	3.67	6.65	3.49	1.69	1.69	1.66
YaeP	hypothetical protein (NCBI) [Escherichia coli K12]	VIMSS1936225	7.33	9.31	4.66	4.22	4.24	3.32
YaeT	hypothetical protein (NCBI) [Escherichia coli K12]	VIMSS14323	12.22	11.97	15.13	10.13	7.63	7.46
YagU	conserved inner membrane protein (NCBI) [Escherichia coli K12]	VIMSS14425	4.89	3.99	4.66	0.84	1.69	1.66
YaiN	putative alpha helix chain (VIMSS) [Escherichia coli K12]	VIMSS14495	0	1.33	0	0	1.69	0
YajC	preprotein translocase subunit YajC (NCBI) [Escherichia coli K12]	VIMSS14545	3.67	0	3.49	2.53	3.39	4.14
YajG	putative polymerase/proteinase (VIMSS) [Escherichia coli K12]	VIMSS14572	0	0	2.33	3.38	2.54	1.66
YajQ	orf, hypothetical protein (VIMSS) [Escherichia coli K12]	VIMSS14564	11	7.98	12.81	7.6	10.17	9.12
YbaB	hypothetical protein (NCBI) [Escherichia coli K12]	VIMSS14608	3.67	2.66	2.33	9.29	6.78	5.8
YbaD	hypothetical protein (NCBI) [Escherichia coli K12]	VIMSS14551	1.22	1.33	0	2.53	2.54	1.66
YbaK	hypothetical protein (NCBI) [Escherichia coli K12]	VIMSS14618	0	1.33	1.16	1.69	1.69	0.83
YbbK	predicted protease, membrane anchored (NCBI) [Escherichia coli K12]	VIMSS14626	2.44	1.33	3.49	0.84	0	0
YbbN	putative thioredoxin-like protein (VIMSS) [Escherichia coli K12]	VIMSS14629	2.44	2.66	2.33	2.53	1.69	0.83
YbbO	short chain dehydrogenase (NCBI) [Escherichia coli K12]	VIMSS14630	2.44	1.33	1.16	2.53	1.69	2.49
YbeB	orf, hypothetical protein (VIMSS) [Escherichia coli K12]	VIMSS14774	0	0	0	1.69	2.54	2.49
YbeD	hypothetical protein (NCBI) [Escherichia coli K12]	VIMSS14768	1.22	1.33	1.16	2.53	2.54	2.49
YbeX	predicteed ion transport (NCBI) [Escherichia coli K12]	VIMSS14795	0	0	0	0.84	2.54	0.83
YbeZ	predicted protein with nucleoside triphosphate hydrolase domain (NCBI) [Escherichia coli K12]	VIMSS14797	9.77	7.98	4.66	14.36	11.02	12.43
YbfF	hypothetical protein (NCBI) [Escherichia coli K12]	VIMSS14816	0	0	0	1.69	0.85	1.66
YbgI	conserved metal-binding protein (NCBI) [Escherichia coli K12]	VIMSS14842	0	0	1.16	2.53	3.39	2.49

YbgK	predicted enzyme subunit (NCBI) [Escherichia coli K12]	VIMSS14844	2.44	0	1.16	1.69	1.69	1.66
YbiB	hypothetical protein (NCBI) [Escherichia coli K12]	VIMSS14925	6.11	5.32	4.66	10.13	8.47	9.95
YbiC	predicted dehydrogenase (NCBI) [Escherichia coli K12]	VIMSS14926	9.77	7.98	4.66	5.91	3.39	4.14
YbiS	hypothetical protein (NCBI) [Escherichia coli K12]	VIMSS14944	12.22	7.98	4.66	6.76	5.08	4.14
YbiT	fused predicted transporter subunits of ABC superfamily: ATP-binding components (NCBI) [Escherichia coli K12]	VIMSS14945	3.67	3.99	2.33	5.07	5.93	5.8
YbiV	predicted hydrolase (NCBI) [Escherichia coli K12]	VIMSS14947	0	1.33	1.16	1.69	2.54	1.66
YbjP	predicted lipoprotein (NCBI) [Escherichia coli K12]	VIMSS14990	1.22	1.33	1.16	1.69	1.69	0.83
YcaJ	recombination protein (NCBI) [Escherichia coli K12]	VIMSS15016	0	0	0	0	0.85	1.66
YcbB	predicted carboxypeptidase (NCBI) [Escherichia coli K12]	VIMSS15049	3.67	3.99	2.33	1.69	3.39	1.66
YcbL	predicted metal-binding enzyme (NCBI) [Escherichia coli K12]	VIMSS15051	2.44	1.33	1.16	0.84	0	0.83
YcbX	predicted 2Fe-2S cluster-containing protein (NCBI) [Escherichia coli K12]	VIMSS15071	0	1.33	0	2.53	1.69	2.49
YcdO	hypothetical protein (NCBI) [Escherichia coli K12]	VIMSS15141	0	0	0	0.84	0.85	1.66
YcdW	putative dehydrogenase (VIMSS) [Escherichia coli K12]	VIMSS15155	2.44	7.98	6.99	10.13	8.47	7.46
YcdX	hypothetical protein (NCBI) [Escherichia coli K12]	VIMSS15156	0	0	0	2.53	1.69	2.49
YceF	orf, hypothetical protein (VIMSS) [Escherichia coli K12]	VIMSS15209	0	0	0	1.69	1.69	0
YceH	hypothetical protein (NCBI) [Escherichia coli K12]	VIMSS15189	4.89	1.33	3.49	6.76	6.78	5.8
YcfB	orf, hypothetical protein (VIMSS) [Escherichia coli K12]	VIMSS15255	0	1.33	1.16	2.53	2.54	4.97
YcfF	orf, hypothetical protein (VIMSS) [Escherichia coli K12]	VIMSS15225	4.89	9.31	5.82	3.38	5.08	5.8
YcfM	predicted outer membrane lipoprotein (NCBI) [Escherichia coli K12]	VIMSS15227	4.89	3.99	5.82	5.91	5.93	4.97
YcfP	orf, hypothetical protein (VIMSS) [Escherichia coli K12]	VIMSS15230	3.67	3.99	2.33	1.69	0.85	0.83
YcfV	putative ATP-binding component of a transport system (VIMSS) [Escherichia coli K12]	VIMSS15239	0	1.33	1.16	1.69	1.69	2.49
YcgC	putative PTS system enzyme I (VIMSS) [Escherichia coli K12]	VIMSS15320	2.44	0	0	2.53	2.54	1.66
YcgK	hypothetical protein (NCBI) [Escherichia coli K12]	VIMSS15300	7.33	9.31	8.15	6.76	7.63	6.63
YchF	translation-associated GTPase (NCBI) [Escherichia coli K12]	VIMSS15325	7.33	6.65	9.31	7.6	6.78	9.95
YchN	hypothetical protein (NCBI) [Escherichia coli K12]	VIMSS15341	2.44	3.99	4.66	4.22	2.54	3.32
YciK	short chain dehydrogenase (NCBI) [Escherichia coli K12]	VIMSS15391	6.11	5.32	6.99	10.13	5.93	8.29
YciO	orf, hypothetical protein (VIMSS) [Escherichia coli K12]	VIMSS15387	2.44	2.66	4.66	2.53	1.69	2.49
YciT	predicted DNA-binding transcriptional regulator (NCBI) [Escherichia coli K12]	VIMSS15404	3.67	5.32	5.82	2.53	2.54	2.49
YcjG	putative muconate cycloisomerase I (EC 5.5.-.-) (VIMSS) [Escherichia coli K12]	VIMSS15445	0	0	1.16	1.69	1.69	2.49
YdcF	hypothetical protein (NCBI) [Escherichia coli K12]	VIMSS15536	0	1.33	1.16	1.69	1.69	0.83
YdcL	predicted lipoprotein (NCBI) [Escherichia coli K12]	VIMSS15552	0	0	0	0.84	0.85	1.66
YdcY	hypothetical protein (NCBI) [Escherichia coli K12]	VIMSS15567	1.22	2.66	1.16	0.84	2.54	1.66
YdfG	L-allo-threonine dehydrogenase, NAD(P)-binding (NCBI) [Escherichia coli K12]	VIMSS15661	2.44	5.32	3.49	4.22	4.24	4.97
YdgA	hypothetical protein (NCBI) [Escherichia coli K12]	VIMSS15735	6.11	3.99	5.82	3.38	4.24	4.14
YdgH	hypothetical protein (NCBI) [Escherichia coli K12]	VIMSS15725	8.55	7.98	6.99	7.6	7.63	7.46
YdhD	hypothetical protein (NCBI) [Escherichia coli K12]	VIMSS15775	7.33	10.64	6.99	8.45	7.63	6.63
YdiA	hypothetical protein (NCBI) [Escherichia coli K12]	VIMSS15822	0	0	0	2.53	4.24	4.14
YdiJ	predicted FAD-linked oxidoreductase (NCBI) [Escherichia coli K12]	VIMSS15806	2.44	3.99	4.66	3.38	1.69	1.66
YdjA	predicted oxidoreductase (NCBI) [Escherichia coli K12]	VIMSS15883	3.67	5.32	5.82	5.07	5.93	5.8
YdjN	predicted transporter (NCBI) [Escherichia coli K12]	VIMSS15847	4.89	2.66	5.82	5.91	6.78	7.46
YeaD	orf, hypothetical protein (VIMSS) [Escherichia coli K12]	VIMSS15898	0	2.66	0	1.69	0.85	1.66
YebC	hypothetical protein (NCBI) [Escherichia coli K12]	VIMSS15982	1.22	2.66	1.16	1.69	0.85	1.66
YebL	putative adhesin (VIMSS) [Escherichia coli K12]	VIMSS15975	21.99	26.6	23.28	31.25	31.35	28.18
YebR	orf, hypothetical protein (VIMSS) [Escherichia coli K12]	VIMSS15950	0	0	1.16	1.69	1.69	2.49
YecA	conserved metal-binding protein (NCBI) [Escherichia coli K12]	VIMSS16025	3.67	1.33	2.33	1.69	0.85	0.83
YecO	predicted methyltransferase (NCBI) [Escherichia coli K12]	VIMSS15988	1.22	1.33	1.16	3.38	1.69	4.14
YeeX	putative alpha helix protein (VIMSS) [Escherichia coli K12]	VIMSS16115	3.67	5.32	4.66	5.91	5.08	7.46
YeeZ	predicted epimerase, with NAD(P)-binding Rossmann-fold domain (NCBI) [Escherichia coli K12]	VIMSS16124	9.77	6.65	6.99	12.67	8.47	9.12
YegQ	predicted peptidase (NCBI) [Escherichia coli K12]	VIMSS16189	0	0	1.16	0	0	1.66
YeiG	predicted esterase (NCBI) [Escherichia coli K12]	VIMSS16263	1.22	0	0	1.69	0.85	1.66
YeiP	putative elongation factor (VIMSS) [Escherichia coli K12]	VIMSS16280	2.44	2.66	1.16	0.84	1.69	1.66
YejK	nucleoid-associated protein NdpA (NCBI) [Escherichia coli K12]	VIMSS16295	3.67	2.66	2.33	5.91	5.93	8.29
YfbF	undecaprenyl phosphate-L-Ara4FN transferase (NCBI) [Escherichia coli K12]	VIMSS16361	4.89	6.65	4.66	5.07	7.63	6.63
YfbG	hypothetical protein (NCBI) [Escherichia coli K12]	VIMSS16362	15.88	15.96	13.97	10.98	16.95	14.92
YfbQ	aspartate aminotransferase (NCBI) [Escherichia coli K12]	VIMSS16397	1.22	0	1.16	1.69	0.85	0.83
YfbT	putative phosphatase (VIMSS) [Escherichia coli K12]	VIMSS16400	2.44	5.32	4.66	5.91	10.17	5.8
YfcB	putative adenine-specific methylase (VIMSS) [Escherichia coli K12]	VIMSS16437	2.44	1.33	1.16	4.22	1.69	1.66
YfcD	predicted NUDIX hydrolase (NCBI) [Escherichia coli K12]	VIMSS16406	1.22	1.33	2.33	0.84	0	0.83
YfcH	conserved protein with NAD(P)-binding Rossmann-fold domain (NCBI) [Escherichia coli K12]	VIMSS16411	1.22	1.33	0	0.84	2.54	0.83
YfdZ	hypothetical protein (NCBI) [Escherichia coli K12]	VIMSS16484	0	0	0	0	1.69	0.83
YfeY	hypothetical protein (NCBI) [Escherichia coli K12]	VIMSS16531	2.44	0	1.16	0	1.69	0.83
YffB	hypothetical protein (NCBI) [Escherichia coli K12]	VIMSS16570	0	0	0	2.53	0	1.66

YfgA	hypothetical protein (NCBI) [Escherichia coli K12]	VIMSS16615	6.11	5.32	6.99	3.38	4.24	4.14
YfgL	protein assembly complex, lipoprotein component (NCBI) [Escherichia coli K12]	VIMSS16611	13.44	11.97	6.99	12.67	3.39	4.14
YfgM	hypothetical protein (NCBI) [Escherichia coli K12]	VIMSS16612	9.77	15.96	8.15	6.76	5.08	4.97
YfhJ	hypothetical protein (NCBI) [Escherichia coli K12]	VIMSS16623	3.67	3.99	2.33	0.84	0.85	0.83
YfhQ	predicted methyltransferase (NCBI) [Escherichia coli K12]	VIMSS16631	3.67	1.33	1.16	1.69	0.85	1.66
YfiF	predicted methyltransferase (NCBI) [Escherichia coli K12]	VIMSS16680	8.55	5.32	2.33	5.91	5.08	4.97
YfiO	predicted lipoprotein (NCBI) [Escherichia coli K12]	VIMSS16690	4.89	6.65	5.82	7.6	3.39	5.8
YgdH	hypothetical protein (NCBI) [Escherichia coli K12]	VIMSS16879	1.22	2.66	3.49	4.22	3.39	4.14
YgdK	predicted Fe-S metabolism protein (NCBI) [Escherichia coli K12]	VIMSS16895	1.22	0	2.33	3.38	3.39	3.32
YgfB	orf, hypothetical protein (VIMSS) [Escherichia coli K12]	VIMSS16989	1.22	2.66	2.33	0.84	2.54	0.83
YgfZ	putative global regulator (NCBI) [Escherichia coli K12]	VIMSS16978	7.33	9.31	3.49	8.45	8.47	7.46
YggH	tRNA(m7G46)-methyltransferase (NCBI) [Escherichia coli K12]	VIMSS17039	0	0	0	1.69	1.69	1.66
YggJ	orf, hypothetical protein (VIMSS) [Escherichia coli K12]	VIMSS17025	0	0	0	0	0	2.49
YggV	putative deoxyribonucleotide triphosphate pyrophosphatase (NCBI) [Escherichia coli K12]	VIMSS17033	1.22	0	3.49	3.38	0.85	3.32
YggW	coproporphyrinogen III oxidase (NCBI) [Escherichia coli K12]	VIMSS17034	0	0	0	0.84	0.85	1.66
YggX	hypothetical protein (NCBI) [Escherichia coli K12]	VIMSS17041	1.22	1.33	0	0.84	1.69	2.49
YgiC	predicted enzyme (NCBI) [Escherichia coli K12]	VIMSS17114	2.44	2.66	0	1.69	1.69	2.49
YgiF	predicted adenylate cyclase (NCBI) [Escherichia coli K12]	VIMSS17130	1.22	3.99	3.49	2.53	3.39	2.49
YgiN	quinol monooxygenase (NCBI) [Escherichia coli K12]	VIMSS17105	2.44	2.66	2.33	2.53	2.54	2.49
YgiW	hypothetical protein (NCBI) [Escherichia coli K12]	VIMSS17100	0	0	1.16	0.84	1.69	2.49
YgjF	G/U mismatch-specific DNA glycosylase (NCBI) [Escherichia coli K12]	VIMSS17144	1.22	0	0	0.84	1.69	1.66
YhbG	predicted transporter subunit: ATP-binding component of ABC superfamily (NCBI) [Escherichia coli K12]	VIMSS17272	3.67	3.99	4.66	5.07	2.54	5.8
YhbH	predicted ribosome-associated, sigma 54 modulation protein (NCBI) [Escherichia coli K12]	VIMSS17274	0	0	0	0.84	1.69	0.83
YhbJ	hypothetical protein (NCBI) [Escherichia coli K12]	VIMSS17276	2.44	2.66	3.49	4.22	6.78	4.97
YhbL	sigma cross-reacting protein 27A (SCRP-27A) (VIMSS) [Escherichia coli K12]	VIMSS17280	6.11	2.66	4.66	3.38	3.39	6.63
YhbS	predicted acyltransferase with acyl-CoA N-acyltransferase domain (NCBI) [Escherichia coli K12]	VIMSS17229	3.67	5.32	6.99	3.38	3.39	4.14
YhbY	predicted RNA-binding protein (NCBI) [Escherichia coli K12]	VIMSS17251	0	0	1.16	0.84	2.54	1.66
YhcB	orf, hypothetical protein (VIMSS) [Escherichia coli K12]	VIMSS17304	17.1	25.27	19.79	10.13	13.56	13.26
YhdH	predicted oxidoreductase, Zn-dependent and NAD(P)-binding (NCBI) [Escherichia coli K12]	VIMSS17323	1.22	1.33	1.16	1.69	0.85	2.49
YhgF	orf, hypothetical protein (VIMSS) [Escherichia coli K12]	VIMSS17470	1.22	2.66	0	1.69	1.69	2.49
YhhK	hypothetical protein (NCBI) [Escherichia coli K12]	VIMSS17520	0	0	0	0.84	1.69	1.66
YhhX	predicted oxidoreductase with NAD(P)-binding Rossmann-fold domain (NCBI) [Escherichia coli K12]	VIMSS17501	2.44	2.66	0	1.69	0.85	1.66
YhiI	predicted HlyD family secretion protein (NCBI) [Escherichia coli K12]	VIMSS17548	13.44	5.32	10.48	2.53	1.69	1.66
YhjJ	predicted zinc-dependent peptidase (NCBI) [Escherichia coli K12]	VIMSS17588	2.44	3.99	2.33	1.69	2.54	2.49
YiaD	predicted outer membrane lipoprotien (NCBI) [Escherichia coli K12]	VIMSS1937052	3.67	9.31	4.66	3.38	1.69	4.97
YiaE	putative dehydrogenase (VIMSS) [Escherichia coli K12]	VIMSS17613	1.22	5.32	4.66	5.07	6.78	4.97
YiaF	orf, hypothetical protein (VIMSS) [Escherichia coli K12]	VIMSS17614	2.44	1.33	3.49	3.38	3.39	3.32
YibN	predicted rhodanese-related sulfurtransferase (NCBI) [Escherichia coli K12]	VIMSS17672	7.33	3.99	5.82	7.6	5.93	7.46
YibT	hypothetical protein (NCBI) [Escherichia coli K12]	VIMSS1937066	3.67	2.66	4.66	0.84	1.69	0.83
YicC	hypothetical protein (NCBI) [Escherichia coli K12]	VIMSS17705	3.67	2.66	3.49	3.38	4.24	2.49
YicH	hypothetical protein (NCBI) [Escherichia coli K12]	VIMSS17716	2.44	5.32	3.49	2.53	3.39	1.66
YidA	predicted hydrolase (NCBI) [Escherichia coli K12]	VIMSS17756	3.67	9.31	2.33	5.07	5.93	4.14
YidC	inner membrane protein translocase component YidC (NCBI) [Escherichia coli K12]	VIMSS17765	8.55	9.31	8.15	7.6	6.78	8.29
YieF	chromate reductase, Class I, flavoprotein (NCBI) [Escherichia coli K12]	VIMSS17773	3.67	2.66	4.66	0.84	2.54	2.49
YifE	hypothetical protein (NCBI) [Escherichia coli K12]	VIMSS17817	2.44	2.66	2.33	2.53	1.69	2.49
YigB	predicted hydrolase (NCBI) [Escherichia coli K12]	VIMSS17861	0	1.33	0	4.22	2.54	3.32
YigI	orf, hypothetical protein (VIMSS) [Escherichia coli K12]	VIMSS17868	0	1.33	1.16	1.69	0.85	1.66
YihA	orf, hypothetical protein (VIMSS) [Escherichia coli K12]	VIMSS17905	2.44	1.33	2.33	2.53	2.54	2.49
YihD	hypothetical protein (NCBI) [Escherichia coli K12]	VIMSS17899	2.44	2.66	3.49	2.53	5.08	4.14
YiiM	orf, hypothetical protein (VIMSS) [Escherichia coli K12]	VIMSS17950	3.67	3.99	2.33	2.53	2.54	1.66
YiiT	stress-induced protein (NCBI) [Escherichia coli K12]	VIMSS17962	4.89	3.99	2.33	3.38	5.08	0.83
YiiU	hypothetical protein (NCBI) [Escherichia coli K12]	VIMSS17967	7.33	6.65	8.15	3.38	3.39	4.97
YijP	conserved inner membrane protein (NCBI) [Escherichia coli K12]	VIMSS17994	3.67	5.32	5.82	7.6	9.32	9.12
YjcD	predicted permease (NCBI) [Escherichia coli K12]	VIMSS18092	0	0	1.16	0	0	1.66
YjdB	orf, hypothetical protein (VIMSS) [Escherichia coli K12]	VIMSS18142	1.22	0	0	1.69	1.69	0.83
YjdC	orf, hypothetical protein (VIMSS) [Escherichia coli K12]	VIMSS18162	3.67	3.99	6.99	7.6	9.32	5.8
YjeB	predicted DNA-binding transcriptional regulator (NCBI) [Escherichia coli K12]	VIMSS18203	0	0	0	1.69	0.85	1.66
YjeE	ATPase with strong ADP affinity (NCBI) [Escherichia coli K12]	VIMSS18193	0	0	0	2.53	0.85	0.83
YjeI	orf, hypothetical protein (VIMSS) [Escherichia coli K12]	VIMSS18172	3.67	5.32	3.49	2.53	0.85	0.83
YjeR	orf, hypothetical protein (VIMSS) [Escherichia coli K12]	VIMSS18190	0	1.33	0	1.69	0.85	0.83
YjgA	hypothetical protein (NCBI) [Escherichia coli K12]	VIMSS18259	1.22	1.33	0	1.69	0	0
YjgF	orf, hypothetical protein (VIMSS) [Escherichia coli K12]	VIMSS18268	7.33	3.99	8.15	5.07	5.08	3.32

YjiA	orf, hypothetical protein (VIMSS) [Escherichia coli K12]	VIMSS18376	2.44	1.33	2.33	0.84	5.93	4.97
YjjK	fused predicted transporter subunits of ABC superfamily: ATP-binding components (NCBI) [Escherichia coli K12]	VIMSS18412	11	10.64	9.31	15.2	11.86	13.26
YkgM	50S ribosomal protein L31 (NCBI) [Escherichia coli K12]	VIMSS14434	3.67	7.98	2.33	6.76	4.24	4.97
YliA	fused predicted peptide transport subunits of ABC superfamily: ATP-binding components (NCBI) [Escherichia coli K12]	VIMSS14954	0	1.33	0	1.69	1.69	1.66
YliB	predicted peptide transporter subunit: periplasmic-binding component of ABC superfamily (NCBI) [Escherichia coli K12]	VIMSS14955	1.22	3.99	2.33	2.53	4.24	4.97
YliJ	putative transferase (VIMSS) [Escherichia coli K12]	VIMSS14963	12.22	1.33	11.64	16.05	12.71	11.6
YmbA	hypothetical protein (NCBI) [Escherichia coli K12]	VIMSS15076	0	1.33	1.16	1.69	0.85	0.83
YniA	predicted phosphotransferase/kinase (NCBI) [Escherichia coli K12]	VIMSS15843	1.22	0	2.33	1.69	2.54	1.66
YniC	predicted hydrolase (NCBI) [Escherichia coli K12]	VIMSS15845	1.22	2.66	0	0.84	1.69	1.66
YodA	conserved metal-binding protein (NCBI) [Escherichia coli K12]	VIMSS16086	1.22	1.33	1.16	0.84	1.69	3.32
YqhD	alcohol dehydrogenase, NAD(P)-dependent (NCBI) [Escherichia coli K12]	VIMSS17088	1.22	2.66	2.33	3.38	2.54	2.49
YqhE	orf, hypothetical protein (VIMSS) [Escherichia coli K12]	VIMSS17089	1.22	2.66	3.49	3.38	2.54	2.49
YraL	predicted methyltransferase (NCBI) [Escherichia coli K12]	VIMSS17219	3.67	5.32	4.66	5.91	5.08	4.14
YraM	hypothetical protein (NCBI) [Escherichia coli K12]	VIMSS17220	0	0	0	1.69	0.85	0.83
YraP	hypothetical protein (NCBI) [Escherichia coli K12]	VIMSS17223	3.67	2.66	2.33	5.91	5.93	6.63
YrbC	predicted ABC-type organic solvent transporter (NCBI) [Escherichia coli K12]	VIMSS17263	2.44	1.33	2.33	5.07	5.93	4.97
YrbD	predicted ABC-type organic solvent transporter (NCBI) [Escherichia coli K12]	VIMSS17264	2.44	1.33	2.33	2.53	1.69	4.97
YrbF	predicted toluene transporter subunit: ATP-binding component of ABC superfamily (NCBI) [Escherichia coli K12]	VIMSS17266	1.22	2.66	1.16	1.69	0.85	1.66
YrdA	putative transferase (VIMSS) [Escherichia coli K12]	VIMSS17342	2.44	1.33	3.49	5.07	4.24	4.14
YrdC	predicted ribosome maturation factor (NCBI) [Escherichia coli K12]	VIMSS17345	1.22	1.33	1.16	0.84	1.69	2.49
YrfI	orf, hypothetical protein (VIMSS) [Escherichia coli K12]	VIMSS17464	0	1.33	0	0	1.69	0
YsgA	predicted hydrolase (NCBI) [Escherichia coli K12]	VIMSS1937151	2.44	1.33	2.33	0.84	1.69	2.49
YtfP	hypothetical protein (NCBI) [Escherichia coli K12]	VIMSS18247	2.44	2.66	2.33	4.22	2.54	2.49
ZapA	protein that localizes to the cytokinetic ring (NCBI) [Escherichia coli K12]	VIMSS16990	3.67	6.65	6.99	3.38	3.39	2.49
ZipA	cell division protein ZipA (NCBI) [Escherichia coli K12]	VIMSS16511	0	2.66	0	0.84	0.85	0.83
ZnuC	high-affinity zinc transporter ATPase (NCBI) [Escherichia coli K12]	VIMSS15976	6.11	2.66	4.66	5.07	5.93	2.49
Zwf	glucose-6-phosphate 1-dehydrogenase (NCBI) [Escherichia coli K12]	VIMSS15970	12.22	9.31	15.13	16.05	13.56	15.75
