# Supplementary material for: Manipulation of the carbon storage regulator system for metabolite remodeling and biofuel production in Escherichia coli
Source: Microb Cell Fact. 2012 Jun 13;11:79. doi: 10.1186/1475-2859-11-79 (PMC3460784; doi:10.1186/1475-2859-11-79)
Supplement: Additional file 5 — Table S4. Predicted CsrA binding sites. [file 1475-2859-11-79-S5.pdf]

Table S4. Predicted CsrA binding sites

Protein ID	Description	BlatID	Gene_Coords	Strand	Motif0	Motif1	Motif8	Site_D	Site_Seq	Log_2 Fold Change
**YhiI**	predicted HlyD family secretion protein	b3487	3627558..3628625	-	Y	Y	Y	-15	CAGGGATT(0,1,8)	2.31
**GlgC**	glucose-1-phosphate adenylyltransferase	b3430	3566056..3567351	-	Y	Y	Y	-6	AAAGGAGT(0,1,8)	2.17
**TolC**	outer membrane channel; specific tolerance to colicin E1; segregation of daughter chromosomes	b3035	3176137..3177618	+	Y	Y	Y	-5	CAAGGAAT(0,1,8)	1.66
**DksA**	DNA-binding transcriptional regulator of rRNA transcription, DnaK suppressor protein	b0145	160149..160604	-	Y	Y	Y	-7	TAAGGAGA(0,1,8)	1.38
**Pck**	phosphoenolpyruvate carboxykinase	b3403	3530840..3532462	+	N	N	Y	-7	TAAGGAGC(8)	1.24
**CysD**	sulfate adenylyltransferase subunit 2	b2752	2873443..2874351	-	N	N	Y	-5	AAAGGAAC(8)	1
**Gst**	glutathionine S-transferase	b1635	1712401..1713006	+	Y	Y	Y	-6	AAAGGAGT(0,1,8)	0.96
**MinD**	membrane ATPase of the MinC-MinD-MinE system	b1175	1223772..1224584	-	Y	Y	Y	-5	CAAGGAAT(0,1,8)	0.86
**YiaD**	predicted outer membrane lipoprotien	b3552	3714570..3715229	+	Y	Y	Y	-5	TAAGGATT(0,1,8)	0.82
**PhoP**	DNA-binding response regulator in two-component regulatory system with PhoQ	b1130	1188999..1189670	-	Y	Y	Y	-8	CAGGGAGA(0,1,8)	0.78
**CysM**	cysteine synthase B (O-acetylserine sulfhydrolase B)	b2421	2536694..2537605	-	Y	Y	Y	-7	TATGGATA(0,1,8)	0.77
**SurA**	peptidyl-prolyl cis-trans isomerase (PPIase)	b0053	53416..54702	-	Y	Y	Y	-5	AATGGAAA(0,1,8)	0.75
**TolB**	translocation protein TolB precursor	b0740	776963..778255	+	Y	Y	Y	-5	AAGGGAGA(0,1,8)	0.74
**IhfB**	integration host factor subunit beta	b0912	963051..963335	+	N	N	Y	-11	TAAGGAAC(8)	0.73
**HflK**	modulator for HflB protease specific for phage lambda cII repressor	b4174	4400061..4401320	+	N	N	Y	-7	TATGGAGC(8)	0.73
**FabD**	acyl carrier protein S-malonyltransferase	b1092	1148951..1149880	+	Y	Y	Y	-6	TAAGGATT(0,1,8)	0.68
**FabI**	enoyl-(acyl carrier protein) reductase	b1288	1348275..1349063	-	Y	Y	Y	-7	TAAGGATT(0,1,8)	0.68
**MscS**	mechanosensitive channel	b2924	3066969..3067829	-	Y	Y	Y	-6	AAAGGAAT(0,1,8)	0.68
**CspC**	stress protein, member of the CspA-family	b1823	1905250..1905459	-	Y	Y	Y	-8	TAAGGAAT(0,1,8)	0.66
**NlpA**	cytoplasmic membrane lipoprotein-28	b3661	3837198..3838016	-	Y	Y	Y	-6	AAAGGATA(0,1,8)	0.57
**YjgF**	orf, hypothetical protein	b4243	4468550..4468936	-	Y	Y	Y	-5	AAAGGAGA(0,1,8)	0.53
**SucA**	alpha-ketoglutarate decarboxylase	b0726	757929..760730	+	N	N	Y	-4	AAGGGATC(8)	0.5
**YcfF**	orf, hypothetical protein	b1103	1161108..1161467	+	Y	Y	Y	-6	AAAGGAAA(0,1,8)	0.5
**UspE**	stress-induced protein	b1333	1395696..1396646	-	Y	Y	Y	-7	TAAGGAGA(0,1,8)	0.49
**Pgm**	phosphoglucomutase	b0688	712781..714421	+	Y	N	N	-6	AAAGGACA(0)	0.48
**TyrS**	tyrosyl-tRNA synthetase	b1637	1713972..1715246	-	Y	Y	Y	-6	CATGGAGA(0,1,8)	0.48
**AspC**	aspartate aminotransferase, PLP-dependent	b0928	983742..984932	-	N	N	Y	-7	AATGGAAC(8)	0.46
**SucB**	dihydrolipoamide acetyltransferase	b0727	760745..761962	+	Y	Y	Y	-5	AAAGGATA(0,1,8)	0.45
**YdgA**	hypothetical protein	b1614	1687876..1689384	+	Y	Y	Y	-7	TATGGATA(0,1,8)	0.44
**ThrS**	threonyl-tRNA synthetase	b1719	1798666..1800594	-	Y	Y	Y	-5	TAAGGATA(0,1,8)	0.44
**NlpB**	lipoprotein-34	b2477	2595853..2596887	-	Y	Y	Y	-5	TAGGGAGA(0,1,8)	0.44
**RpsF**	30S ribosomal protein S6	b4200	4423141..4423536	+	N	N	Y	-7	TAAGGAGC(8)	0.44
**PntA**	NAD(P) transhydrogenase subunit alpha	b1603	1674395..1675927	-	Y	Y	Y	-4	AAGGGAAT(0,1,8)	0.43
**SodB**	superoxide dismutase, Fe	b1656	1733402..1733983	+	Y	Y	Y	-7	AAAGGAGA(0,1,8)	0.4
**Pnp**	polynucleotide phosphorylase/polyadenylase	b3164	3307055..3309190	-	Y	Y	Y	-6	AAAGGATA(0,1,8)	0.36
**SseA**	putative thiosulfate sulfurtransferase	b2521	2650516..2651361	+	Y	Y	Y	-6	AATGGAGA(0,1,8)	0.35
**HlpA**	periplasmic chaperone	b0178	200482..200967	+	Y	Y	Y	-6	TAAGGAGT(0,1,8)	0.32
**Mdh**	malate dehydrogenase	b3236	3381352..3382290	-	Y	Y	Y	-6	TAAGGAGT(0,1,8)	0.32
**HupA**	HU, DNA-binding transcriptional regulator, alpha subunit	b4000	4198304..4198576	+	Y	Y	Y	-5	TAAGGATA(0,1,8)	0.29
**TrpB**	tryptophan synthase subunit beta	b1261	1315246..1316439	-	Y	Y	Y	-2,-7	AAAGGAAC(8),TAAGGAAA(0,1,8)	0.27
**DeoD**	purine nucleoside phosphorylase	b4384	4618906..4619625	+	Y	Y	Y	-6	AAAGGATA(0,1,8)	0.27
**CysK**	cysteine synthase A, O-acetylserine sulfhydrolase A subunit	b2414	2530431..2531402	+	Y	N	N	-5	TAAGGACA(0)	0.25
**YajQ**	orf, hypothetical protein	b0426	443907..444398	+	Y	Y	Y	-8	AAGGGAGA(0,1,8)	0.24
**ProA**	gamma-glutamyl phosphate reductase	b0243	260727..261980	+	N	N	Y	-7	TAAGGAGC(8)	0.23
**Pps**	phosphoenolpyruvate synthase	b1702	1782758..1785136	-	Y	Y	Y	-6	AAAGGATT(0,1,8)	0.2
**HslU**	ATP-dependent protease ATP-binding subunit	b3931	4118439..4119770	-	N	N	Y	-5	TAAGGATC(8)	0.16
**MaeB**	malic enzyme	b2463	2574120..2576399	-	N	N	Y	-7	AAAGGAAC(8)	0.13
**SecD**	protein export protein SecD	b0408	426871..428718	+	Y	Y	Y	-5	AAGGGAAT(0,1,8)	0.1
**UspG**	universal stress protein UP12	b0607	640662..641090	-	Y	Y	Y	-8	CAGGGAGA(0,1,8)	0.1
**YfbG**	hypothetical protein	b2255	2366061..2368043	+	Y	Y	Y	-8	CAAGGAAA(0,1,8)	0.1
**RpsG**	30S ribosomal protein S7	b3341	3471564..3472103	-	Y	N	N	-7	AACGGAGT(0)	0.06
**MoaB**	molybdopterin biosynthesis protein B	b0782	817278..817790	+	Y	Y	Y	-7	AAAGGAGA(0,1,8)	0.05
**HisC**	histidinol-phosphate aminotransferase	b2021	2090422..2091492	+	N	N	Y	-5	TAAGGAGC(8)	0.02
**AspS**	aspartyl-tRNA synthetase	b1866	1946774..1948546	-	Y	Y	Y	-5	AAGGGATA(0,1,8)	-0.02
**ArgH**	argininosuccinate lyase	b3960	4154873..4156246	+	Y	Y	Y	-8	TAAGGAAA(0,1,8)	-0.03
**PepP**	proline aminopeptidase P II	b2908	3051537..3052862	-	Y	Y	Y	-6	TAAGGAGA(0,1,8)	-0.11
**GroS**	co-chaperonin GroES	b4142	4368711..4369004	+	Y	Y	Y	-8	AAAGGAGA(0,1,8)	-0.13
**PurD**	phosphoribosylamine--glycine ligase	b4005	4202665..4203954	-	N	N	Y	-6	AATGGAGC(8)	-0.14
**Frr**	ribosome releasing factor	b0172	192872..193429	+	Y	Y	Y	-7	CAAGGATT(0,1,8)	-0.15
**Upp**	uracil phosphoribosyltransferase	b2498	2618268..2618894	-	Y	Y	Y	-7	AAAGGAGA(0,1,8)	-0.19
**RplP**	50S ribosomal protein L16	b3313	3446781..3447191	-	N	N	Y	-8	TAAGGAGC(8)	-0.19
**IscS**	cysteine desulfurase	b2530	2658339..2659553	-	Y	N	N	-11	TACGGAGT(0)	-0.21
**AceE**	pyruvate dehydrogenase subunit E1	b0114	123017..125680	+	Y	Y	Y	-6	TAAGGAAT(0,1,8)	-0.3
**Zwf**	glucose-6-phosphate 1-dehydrogenase	b1852	1932863..1934338	-	Y	Y	Y	-6	TAAGGAGA(0,1,8)	-0.31
**GlnH**	glutamine ABC transporter periplasmic protein	b0811	846481..847227	-	Y	Y	Y	-5	AAAGGAAA(0,1,8)	-0.34
**GroEL**	chaperonin GroEL	b4143	4369048..4370694	+	Y	Y	Y	-8	TAAGGAAT(0,1,8)	-0.34
**PepD**	aminoacyl-histidine dipeptidase (peptidase D)	b0237	254259..255716	-	Y	Y	Y	-7	CAAGGAGA(0,1,8)	-0.35
**PanC**	pantoate--beta-alanine ligase	b0133	147944..148795	-	Y	Y	Y	-7	TAAGGAGT(0,1,8)	-0.37
**PykA**	pyruvate kinase	b1854	1935673..1937115	+	Y	N	N	-6	AACGGAGT(0)	-0.37
**GltA**	citrate synthase	b0720	752408..753691	-	Y	Y	Y	-7	TAAGGAGA(0,1,8)	-0.38
**LeuB**	3-isopropylmalate dehydrogenase	b0073	80867..81958	-	Y	Y	Y	-8	CAAGGAAA(0,1,8)	-0.4
**Tas**	predicted oxidoreductase, NADP(H)-dependent aldo-keto reductase	b2834	2969619..2970659	+	Y	Y	Y	-6	AAAGGAAA(0,1,8)	-0.4
**LeuD**	isopropylmalate isomerase small subunit	b0071	78848..79453	-	N	N	Y	-6	TAAGGAGC(8)	-0.44
**GlmM**	phosphoglucosamine mutase	b3176	3320755..3322092	-	Y	Y	Y	-10	AAAGGAAA(0,1,8)	-0.49
**NarL**	DNA-binding response regulator in two-component regulatory system with NarX (or NarQ)	b1221	1274402..1275052	-	Y	Y	Y	-6	CAAGGAGA(0,1,8)	-0.59
**IlvB**	acetolactate synthase large subunit	b3671	3849119..3850807	-	Y	N	N	-6	AAAGGACT(0)	-0.6
**IlvE**	branched-chain amino acid aminotransferase	b3770	3950507..3951436	+	Y	Y	Y	-7	AAAGGAAT(0,1,8)	-0.66
**RplD**	50S ribosomal protein L4	b3319	3449703..3450308	-	Y	Y	Y	-6	TAAGGAGA(0,1,8)	-0.81
**YbiB**	hypothetical protein	b0800	834471..835433	+	Y	Y	Y	-6	TAAGGAGT(0,1,8)	-0.82
**GshB**	glutathione synthetase	b2947	3089900..3090850	+	Y	N	N	-7	AACGGAGA(0)	-0.97
**YaaX**	hypothetical protein	b0005	5234..5530	+	Y	Y	Y	-6	AAAGGAGT(0,1,8)	N/A
**YaaA**	hypothetical protein	b0006	5683..6459	-	Y	N	N	-5	CAAGGACT(0)	N/A
**Mog**	molybdenum cofactor biosynthesis protein	b0009	9306..9893	+	Y	N	N	-8	AACGGAAA(0)	N/A
**HokC**	toxic membrane protein, small	b4412	16751..16903	-	Y	Y	Y	-9	CAAGGAGA(0,1,8)	N/A
**NhaR**	DNA-binding transcriptional activator	b0020	18715..19620	+	Y	Y	Y	-4	CAGGGAGA(0,1,8)	N/A
**RihC**	ribonucleoside hydrolase 3	b0030	27293..28207	+	Y	Y	Y	-5	TATGGAGA(0,1,8)	N/A
**CaiD**	crotonobetainyl CoA hydratase	b0036	35377..36162	-	Y	Y	Y	-7	AATGGAGA(0,1,8)	N/A
**CaiC**	predicted crotonobetaine CoA ligase:carnitine CoA ligase	b0037	36271..37824	-	Y	Y	Y	-9	AATGGATA(0,1,8)	N/A
**FixC**	predicted oxidoreductase with FAD/NAD(P)-binding domain	b0043	44180..45466	+	N	N	Y	-5	CAGGGAGC(8)	N/A
**FixX**	predicted 4Fe-4S ferredoxin-type protein	b0044	45463..45750	+	Y	Y	Y	-10	AAAGGAGT(0,1,8)	N/A
**AraA**	L-arabinose isomerase	b0062	66835..68337	-	Y	N	N	-6	TAAGGACA(0)	N/A
**ThiP**	thiamin ABC transporter membrane protein	b0067	72911..74521	-	Y	Y	Y	-6	CATGGATT(0,1,8)	N/A
**SetA**	broad specificity sugar efflux system	b0070	77621..78799	+	N	N	Y	-3	AAGGGAAC(8)	N/A
**IlvH**	acetolactate synthase small subunit	b0078	87357..87848	+	Y	N	N	-11	AACGGAGA(0)	N/A
**FtsI**	transpeptidase involved in septal peptidoglycan synthesis (penicillin-binding protein 3)	b0084	91413..93179	+	Y	Y	Y	-11	TAAGGATA(0,1,8)	N/A
**YacG**	zinc-binding protein	b0101	111649..111846	-	Y	Y	Y	-5	TAAGGAGT(0,1,8)	N/A
**HofB**	conserved protein with nucleoside triphosphate hydrolase domain	b0107	115714..117099	-	N	N	Y	-5	TAAGGAGC(8)	N/A
**PpdD**	predicted major pilin subunit	b0108	117109..117549	-	N	N	Y	-8	CAAGGAGC(8)	N/A
**AmpD**	N-acetyl-anhydromuranmyl-L-alanine amidase	b0110	118733..119284	+	Y	Y	Y	-7	TAAGGAGA(0,1,8)	N/A
**AmpE**	predicted inner membrane protein	b0111	119281..120135	+	Y	Y	Y	-5	CAAGGAGA(0,1,8)	N/A
**SpeD**	S-adenosylmethionine decarboxylase proenzyme	b0120	134788..135582	-	Y	Y	Y	-11	TAAGGAGA(0,1,8)	N/A
**CueO**	multicopper oxidase (laccase)	b0123	137083..138633	+	Y	Y	Y	-6	TAAGGAAA(0,1,8)	N/A
**YadH**	predicted transporter subunit: membrane component of ABC superfamily	b0128	143702..144472	+	Y	Y	Y	-7	CAAGGAGA(0,1,8)	N/A
**YadN**	predicted fimbrial-like adhesin protein	b0141	156299..156883	-	Y	Y	Y	-4	AAAGGATT(0,1,8)	N/A
**SfsA**	sugar fermentation stimulation protein A	b0146	160782..161486	-	Y	Y	Y	-7	CAAGGATT(0,1,8)	N/A
**IspU**	undecaprenyl pyrophosphate synthase	b0174	194903..195664	+	Y	Y	Y	-10	CAGGGAAT(0,1,8)	N/A
**YaeR**	orf, hypothetical protein	b0187	211877..212266	+	Y	Y	Y	-5	AAAGGAGA(0,1,8)	N/A

**YaeQ**	hypothetical protein	b0190	214291..214836	+	Y	Y	Y	-7	AAAGGAGT(0,1,8)	N/A	
**YaeF**	orf, hypothetical protein	b0193	216179..217003	-	Y	Y	Y	-8	CATGGATT(0,1,8)	N/A	
**GmhB**	hypothetical protein	b0200	222833..223408	+	N	N	Y	-9	TAAGGAGC(8)	N/A	
**YafV**	predicted C-N hydrolase family amidase, NAD(P)-binding	b0219	239419..240189	-	Y	Y	Y	-9	AAAGGAGT(0,1,8)	N/A	
**YafK**	hypothetical protein	b0224	245065..245805	-	Y	Y	Y	-8	CAAGGAAT(0,1,8)	N/A	
**YafM**	hypothetical protein	b0228	247637..248134	+	Y	Y	Y	-5	AAAGGAGT(0,1,8)	N/A	
**YafO**	predicted toxin of the YafO-YafN toxin-antitoxin system	b0233	252301..252699	+	Y	Y	Y	-5	TAAGGAAT(0,1,8)	N/A	
**YkfB**	CP4-6 prophage; predicted protein	b0250	264844..265311	-	Y	Y	Y	-6	AAAGGAGA(0,1,8)	N/A	
**YafY**	CP4-6 prophage; predicted DNA-binding transcriptional regulator	b0251	265334..265777	-	Y	N	N	-8	CACGGAGA(0)	N/A	
**PerR**	CP4-6 prophage; predicted DNA-binding transcriptional regulator	b0254	268513..269406	-	Y	Y	Y	-6	AAAGGATT(0,1,8)	N/A	
**AfuC**	putative ATP-binding component of a transport system	b0262	276980..278026	-	N	N	Y	-6	AATGGAGC(8)	N/A	
**YkgA**	putative ARAC-type regulatory protein	b0300	315674..316360	-	Y	N	N	-6	AACGGAGA(0)	N/A	
**YkgE**	predicted oxidoreductase	b0306	320832..321551	+	Y	Y	Y	-8	AATGGAGT(0,1,8)	N/A	
**BetI**	probably transcriptional repressor of bet genes	b0313	327971..328558	-	Y	Y	Y	-8	AATGGAGT(0,1,8)	N/A	
**PrpD**	2-methylcitrate dehydratase	b0334	350439..351890	+	Y	Y	Y	-9	TAAGGAAA(0,1,8)	N/A	
**LacA**	galactoside O-acetyltransferase	b0342	360473..361084	-	Y	N	N	-8	AACGGAGT(0)	N/A	
**LacY**	galactoside permease	b0343	361150..362403	-	Y	Y	Y	-7	TAAGGAAA(0,1,8)	N/A	
**MhpD**	2-keto-4-pentenoate hydratase	b0350	371339..372148	+	Y	Y	Y	-5	TAAGGAAT(0,1,8)	N/A	
**YaiS**	orf, hypothetical protein	b0364	383283..383840	-	Y	Y	Y	-9	AATGGAGT(0,1,8)	N/A	
**TauD**	taurine dioxygenase	b0368	387019..387870	+	Y	Y	Y	-7	CATGGAGA(0,1,8)	N/A	
**AmpH**	beta-lactamase/D-alanine carboxypeptidase	b0376	394354..395511	-	Y	N	N	-7	TATGGACA(0)	N/A	
**YaiW**	predicted DNA-binding transcriptional regulator	b0378	397096..398190	+	Y	Y	Y	-5	AAAGGAGA(0,1,8)	N/A	
**YaiB**	hypothetical protein	b0382	400610..400870	+	Y	Y	Y	-8	TAAGGAAA(0,1,8)	N/A	
**PhoA**	alkaline phosphatase	b0383	400971..402386	+	Y	Y	Y	-8	CATGGAGA(0,1,8)	N/A	
**YajF**	possible NAGC-like transcriptional regulator	b0394	409368..410276	+	Y	Y	Y	-6	TAAGGAGA(0,1,8)	N/A	
**SbcD**	exonuclease, dsDNA, ATP-dependent	b0398	414974..416176	-	N	N	Y	-5	CAGGGAAC(8)	N/A	
**ThiJ**	4-methyl-5(beta-hydroxyethyl)-thiazole monophosphate synthesis	b0424	442275..442865	-	Y	Y	Y	-7	AAAGGAGA(0,1,8)	N/A	
**YbaV**	hypothetical protein	b0442	463161..463532	+	Y	Y	Y	-8	CAAGGAGA(0,1,8)	N/A	
**YbaY**	glycoprotein/polysaccharide metabolism	b0453	474603..475175	+	Y	Y	Y	-6	TAAGGAGA(0,1,8)	N/A	
**YlaB**	orf, hypothetical protein	b0457	476291..477841	-	Y	Y	Y	-6	TAAGGATA(0,1,8)	N/A	
**YbaM**	hypothetical protein	b0466	489334..489495	-	Y	Y	Y	-5	AATGGAGA(0,1,8)	N/A	
**Aes**	acetyl esterase	b0476	498238..499197	-	Y	Y	Y	-7	AAAGGAGT(0,1,8)	N/A	
**YbaL**	predicted transporter with NAD(P)-binding Rossmann-fold domain	b0478	500786..502462	-	Y	Y	Y	-5	AAAGGAGA(0,1,8)	N/A	
**UshA**	UDP-sugar hydrolase	b0480	504138..505790	+	Y	Y	Y	-6	CAGGGAGA(0,1,8)	N/A	
**CopA**	copper transporter	b0484	508099..510603	-	Y	Y	Y	-6	AAAGGAGT(0,1,8)	N/A	
**YbaS**	predicted glutaminase	b0485	510865..511797	+	Y	Y	Y	-10	AAAGGAGT(0,1,8)	N/A	
**YbbJ**	conserved inner membrane protein	b0488	513625..514083	-	Y	N	N	-6	AACGGACT(0)	N/A	
**YbbL**	predicted transporter subunit: ATP-binding component of ABC superfamily	b0490	515143..515820	+	N	N	Y	-5	AAGGGAAC(8)	N/A	
**YbbC**	hypothetical protein	b0498	526805..527173	+	Y	Y	Y	-5	TAAGGAGA(0,1,8)	N/A	
**AllA**	ureidoglycolate hydrolase	b0505	531675..532157	+	Y	N	N	-6	CAAGGACA(0)	N/A	
**Hyi**	hydroxypyruvate isomerase	b0508	534934..535710	+	Y	Y	Y	-7	AAGGGAGA(0,1,8)	N/A	
**AllB**	allantoinase	b0512	538371..539732	+	Y	Y	Y	-6	CAGGGAGT(0,1,8)	N/A	
**GlxK**	glycerate kinase II	b0514	541112..542257	+	Y	Y	Y	-5	AAAGGATT(0,1,8)	N/A	
**YlbA**	hypothetical protein	b0515	542485..543270	-	Y	Y	Y	-6	TAAGGAGT(0,1,8)	N/A	
**FdrA**	membrane protein FdrA	b0518	545904..547571	+	Y	Y	Y	-5	TAAGGAGT(0,1,8)	N/A	
**YbcF**	predicted carbamate kinase	b0521	549662..550555	+	Y	Y	Y	-4	CAAGGAAA(0,1,8)	N/A	
**CysS**	cysteinyl-tRNA synthetase	b0526	553834..555219	+	Y	N	N	-6	AACGGAAT(0)	N/A	
**SfmF**	predicted fimbrial-like adhesin protein	b0534	562553..563068	+	Y	N	N	-1	TACGGAGA(0)	N/A	
**YbcW**	DLP12 prophage; predicted protein	b0559	579103..579309	+	N	N	Y	-7	CATGGAGC(8)	N/A	
**OmpT**	DLP12 prophage; outer membrane protease VII (outer membrane protein 3b)	b0565	583903..584856	-	Y	Y	Y	-7	AATGGAGA(0,1,8)	N/A	
**YbdG**	predicted mechanosensitive channel	b0577	602639..603886	-	Y	Y	Y	-5	AAAGGAGA(0,1,8)	N/A	
**NfnB**	dihydropteridine reductase, NAD(P)H-dependent, oxygen-insensitive	b0578	603994..604647	-	Y	Y	Y	-5	CATGGAGT(0,1,8)	N/A	
**YbdF**	hypothetical protein	b0579	604741..605109	-	N	N	Y	-5	AAAGGAAC(8)	N/A	
**EntE**	2,3-dihydroxybenzoate-AMP ligase component of enterobactin synthase multienzyme complex	b0594	625293..626903	+	N	N	Y	-5	TAAGGAGC(8)	N/A	
**CstA**	carbon starvation protein	b0598	629117..631222	+	Y	N	N	-7	CACGGATA(0)	N/A	
**YbdH**	predicted oxidoreductase	b0599	631612..632700	-	Y	N	N	-8	TAAGGACA(0)	N/A	
**YbdM**	hypothetical protein	b0601	633970..634599	-	Y	Y	Y	-10	AAAGGAAA(0,1,8)	N/A	
**DsbG**	thiol:disulfide interchange protein	b0604	637050..637796	-	Y	N	N	-6	AAAGGACA(0)	N/A	
**Rnk**	nucleoside diphosphate kinase regulator	b0610	642780..643190	-	Y	Y	Y	-5	CATGGAGT(0,1,8)	N/A	
**CitA**	sensory histidine kinase in two-component regulatory system with citB	b0619	651458..653116	+	Y	Y	Y	-4	CAAGGAAA(0,1,8)	N/A	
**LipB**	protein of lipoate biosynthesis	b0630	660860..661501	-	Y	N	N	-8	CACGGAGA(0)	N/A	
**YbeQ**	orf, hypothetical protein	b0644	674793..675770	-	Y	Y	Y	-6	TAAGGAAA(0,1,8)	N/A	
**YbeR**	hypothetical protein	b0645	675934..676641	+	Y	Y	Y	-7	TAAGGATA(0,1,8)	N/A	
**DjlB**	predicted chaperone	b0646	676638..678065	+	Y	Y	Y	-11	TAAGGATA(0,1,8)	N/A	
**YbeU**	predicted tRNA ligase	b0648	678731..679438	+	Y	Y	Y	-9	AAGGGATT(0,1,8)	N/A	
**RihA**	ribonucleoside hydrolase 1	b0651	682700..683635	-	N	N	Y	-5	CAAGGAGC(8)	N/A	
**GltI**	glutamate and aspartate transporter subunit	b0655	686062..686970	-	Y	Y	Y	-6	AAAGGAGT(0,1,8)	N/A	
**AsnB**	asparagine synthetase B	b0674	696736..698400	-	Y	N	N	-6	AACGGAGA(0)	N/A	
**YbfP**	hypothetical protein	b0689	714635..715129	+	Y	Y	Y	-6	CAAGGAAA(0,1,8)	N/A	
**YbfG**	orf, hypothetical protein	b0691	715611..715820	-	N	N	Y	-9	CAAGGAGC(8)	N/A	
**YbfD**	hypothetical protein	b0706	737315..738076	+	Y	Y	Y	-6	TAAGGAGA(0,1,8)	N/A	
**Nei**	endonuclease VIII/ 5-formyluracil/5-hydroxymethyluracil DNA glycosylase	b0714	745158..745949	+	Y	Y	Y	-6	AAAGGATA(0,1,8)	N/A	
**YbgP**	predicted assembly protein	b0717	748202..748930	-	Y	Y	Y	-6	TAAGGAAA(0,1,8)	N/A	
**YbgQ**	putative outer membrane protein	b0718	748945..751392	-	Y	N	N	-3	TATGGACA(0)	N/A	
**YbgD**	predicted fimbrial-like adhesin protein	b0719	751452..752018	-	Y	N	N	-6	AAAGGACT(0)	N/A	
**MngB**	alpha-mannosidase	b0732	767201..769834	+	Y	Y	Y	-6	AAAGGAAA(0,1,8)	N/A	
**CydA**	cytochrome d terminal oxidase, polypeptide subunit I	b0733	770681..772249	+	Y	Y	Y	-5	CAAGGAGT(0,1,8)	N/A	
**YbgT**	hypothetical protein	b4515	773419..773532	+	N	N	Y	-6	TAAGGAGC(8)	N/A	
**Pal**	peptidoglycan-associated outer membrane lipoprotein	b0741	778290..778811	+	Y	Y	Y	-8	AAAGGAAT(0,1,8)	N/A	
**GalM**	galactose-1-epimerase (mutarotase)	b0756	787020..788060	-	N	N	Y	-7	CAAGGAGC(8)	N/A	
**GalT**	galactose-1-phosphate uridylyltransferase	b0758	789206..790252	-	N	N	Y	-5	TAAGGAAC(8)	N/A	
**GalE**	UDP-galactose-4-epimerase	b0759	790262..791278	-	N	N	Y	-6	AATGGAGC(8)	N/A	
**ModB**	molybdate ABC transporter permease protein	b0764	795085..795774	+	Y	N	N	-13	TACGGATT(0)	N/A	
**YbhE**	6-phosphogluconolactonase	b0767	797809..798804	+	N	N	Y	-5	AAAGGAGC(8)	N/A	
**YbhM**	conserved inner membrane protein	b0787	820016..820729	+	Y	Y	Y	-10	TAAGGATA(0,1,8)	N/A	
**YbhR**	predicted transporter subunit: membrane component of ABC superfamily	b0792	824225..825331	-	Y	Y	Y	-6	TAGGGAGA(0,1,8)	N/A	
**YbhS**	predicted transporter subunit: membrane component of ABC superfamily	b0793	825342..826475	-	N	N	Y	-7	TAAGGAGC(8)	N/A	
**YbhG**	hypothetical protein	b0795	828197..829195	-	Y	Y	Y	-6	AAAGGAGT(0,1,8)	N/A	
**YbiH**	putative transcriptional regulator	b0796	829195..829866	-	Y	N	N	-6	AATGGACT(0)	N/A	
**YbiN**	orf, hypothetical protein	b0807	841555..842481	+	Y	Y	Y	-6	AAAGGAGA(0,1,8)	N/A	
**YliC**	predicted peptide transporter subunit: membrane component of ABC superfamily	b0831	870190..871110	+	Y	Y	Y	-4	CAGGGAGT(0,1,8)	N/A	
**DacC**	D-alanyl-D-alanine carboxypeptidase (penicillin-binding protein 6a)	b0839	879950..881152	+	Y	Y	Y	-15	AATGGATA(0,1,8)	N/A	
**PotH**	putrescine transporter subunit: membrane component of ABC superfamily	b0856	895357..896310	+	N	N	Y	-5	TAAGGAGC(8)	N/A	
**YbjP**	predicted lipoprotein	b0865	903175..903690	-	Y	Y	Y	-6	TAAGGAAA(0,1,8)	N/A	
**YbjQ**	hypothetical protein	b0866	903816..904139	+	Y	Y	Y	-7	CAAGGAGA(0,1,8)	N/A	
**YbjT**	putative dTDP-glucose enzyme	b0869	906075..907505	-	Y	Y	Y	-6	TAAGGAGA(0,1,8)	N/A	
**LtaE**	L-allo-threonine aldolase, PLP-dependent	b0870	907516..908517	-	Y	N	N	-5	TAAGGACA(0)	N/A	
**Hcr**	HCP oxidoreductase, NADH-dependent	b0872	910405..911373	-	Y	Y	Y	-7	TAAGGAGT(0,1,8)	N/A	
**YbjE**	putative surface protein	b0874	913181..914080	-	Y	N	N	-9	CAAGGACT(0)	N/A	
**B878**	putative membrane protein	b0878	918458..919573	+	Y	Y	Y	-14	TATGGAGT(0,1,8)	N/A	
**DmsB**	dimethyl sulfoxide reductase, anaerobic, subunit B	b0895	942637..943254	+	Y	Y	Y	-6	TAAGGAGT(0,1,8)	N/A	
**YcaC**	predicted hydrolase	b0897	944154..944780	-	Y	Y	Y	-6	AAAGGAGA(0,1,8)	N/A	
**Cmk**	cytidylate kinase	b0910	960424..961107	+	Y	Y	Y	-6	TAAGGAGA(0,1,8)	N/A	
**YcbC**	conserved inner membrane protein	b0920	971845..972624	-	N	N	Y	-9	AAAGGAAC(8)	N/A	
**MukF**	condesin subunit F	b0922	973542..974864	+	Y	Y	Y	-5	CAAGGATA(0,1,8)	N/A	
**SsuE**	NAD(P)H-dependent FMN reductase	b0937	996160..996735	-	Y	Y	Y	-5	TAAGGAGA(0,1,8)	N/A	
**Uup**	fused predicted transporter subunits of ABC superfamily: ATP-binding components	b0949	1009187..1011094	+	Y	Y	Y	-5	AAAGGAAT(0,1,8)	N/A	
**PqiA**	paraquat-inducible membrane protein A	b0950	1011224..1012477	+	Y	Y	Y	-5	TAAGGAGT(0,1,8)	N/A	
**YccR**	hypothetical protein	b0959	1020361..1020990	+	Y	Y	Y	-4	AAAGGAAT(0,1,8)	N/A	
**YccX**	predicted acylphosphatase	b0968	1029287..1029565	+	Y	Y	Y	-7,-13	TAAGGAGA(0,1,8),TAAGGATA(0,1,8)	N/A	
**HyaD**	protein involved in processing of HyaA and HyaB proteins	b0975	1034993..1035580	+	N	N	Y	-5	CAAGGAGC(8)	N/A	
**AppB**	cytochrome bd-II oxidase, subunit II	b0979	1038519..1039655	+	Y	Y	Y	-6	AAAGGAGA(0,1,8)	N/A	
**YccB**	hypothetical protein	b4592	1039668..1039760	+	N	N	Y	-8	TAAGGAGC(8)	N/A	

**YmcC**	predicted outer membrane lipoprotein	b0986	1047911..1048555	-	Y	Y	Y	-5	CAAGGATT(0,1,8)	N/A
**YmcD**	orf, hypothetical protein	b0987	1048662..1048967	-	Y	Y	Y	-5	AAAGGAAA(0,1,8)	N/A
**CspH**	stress protein, member of the CspA-family	b0989	1050186..1050398	-	Y	Y	Y	-5	TAAGGAAT(0,1,8)	N/A
**YccM**	predicted 4Fe-4S membrane protein	b0992	1051512..1052585	-	Y	N	N	-10	AACGGACT(0)	N/A
**YcdG**	putative transport protein	b1006	1067734..1069062	-	Y	Y	Y	-7	TAAGGAGA(0,1,8)	N/A
**B1007**	orf, hypothetical protein	b1007	1069083..1069577	-	N	N	Y	-6	TAAGGAGC(8)	N/A
**YcdI**	hypothetical protein	b1008	1069588..1070178	-	Y	Y	Y	-5	TAAGGAAT(0,1,8)	N/A
**YcdK**	hypothetical protein	b1010	1070996..1071382	-	Y	Y	Y	-7	TAAGGAGT(0,1,8)	N/A
**YcdO**	hypothetical protein	b1018	1081466..1082593	+	Y	Y	Y	-9	AAGGGATA(0,1,8)	N/A
**PhoH**	conserved protein with nucleoside triphosphate hydrolase domain	b1020	1084215..1085279	+	Y	Y	Y	-7	TATGGAGA(0,1,8)	N/A
**YcdR**	predicted enzyme associated with biofilm formation	b1023	1087062..1089080	-	Y	Y	Y	-4	TAAGGATA(0,1,8)	N/A
**YcdS**	predicted outer membrane protein	b1024	1089089..1091512	-	Y	Y	Y	-9	CATGGAGT(0,1,8)	N/A
**YcdY**	hypothetical protein	b1035	1098863..1099417	+	Y	Y	Y	-8	AATGGAAA(0,1,8)	N/A
**CsgG**	outer membrane lipoprotein	b1037	1100074..1100907	-	Y	Y	Y	-7	TAAGGAAA(0,1,8)	N/A
**YmdC**	putative synthase	b1046	1105578..1106999	+	Y	Y	Y	-9	CAAGGAGA(0,1,8)	N/A
**MsyB**	acidic protein suppresses mutants lacking function of protein export	b1051	1113030..1113404	-	Y	Y	Y	-7	AAGGGAGA(0,1,8)	N/A
**YceI**	hypothetical protein	b1056	1117124..1117699	-	Y	Y	Y	-8	TATGGAGT(0,1,8)	N/A
**YceH**	hypothetical protein	b1067	1125380..1126027	+	Y	Y	Y	-6	TAAGGAGA(0,1,8)	N/A
**FlgD**	flagellar basal body rod modification protein D	b1075	1131077..1131772	+	Y	Y	Y	-6	AAAGGAGA(0,1,8)	N/A
**FlgK**	flagellar hook-associated protein K	b1082	1137601..1139244	+	N	N	Y	-5	TAAGGAAC(8)	N/A
**PabC**	4-amino-4-deoxychorismate lyase	b1096	1152523..1153332	+	N	N	Y	-5	TAAGGAGC(8)	N/A
**Tmk**	thymidylate kinase	b1098	1154347..1154988	+	Y	Y	Y	-4	TAAGGAAA(0,1,8)	N/A
**NagZ**	beta-hexosaminidase	b1107	1163318..1164343	+	Y	Y	Y	-6	TAAGGAGA(0,1,8)	N/A
**YcfS**	hypothetical protein	b1113	1168635..1169597	-	Y	Y	Y	-5	TATGGATT(0,1,8)	N/A
**NagK**	N-acetyl-D-glucosamine kinase	b1119	1177816..1178727	+	N	N	Y	-5	CAAGGAGC(8)	N/A
**IntE**	e14 prophage; predicted integrase	b1140	1198902..1200029	-	Y	N	N	-8	AACGGATT(0)	N/A
**YmfI**	orf, hypothetical protein	b1143	1200720..1201061	+	Y	Y	Y	-5	CAAGGAAA(0,1,8)	N/A
**N/A**	N/A	N/A	1203393..1204760	+	Y	Y	Y	-13	CAGGGAAA(0,1,8)	N/A
**B1171**	orf, hypothetical protein	b1171	1221528..1221857	-	Y	Y	Y	-5	TAAGGAAT(0,1,8)	N/A
**DsbB**	reoxidizes DsbA protein following formation of disulfide bond in P-ring of flagella.	b1185	1231723..1232253	-	Y	Y	Y	-6	CAGGGAAA(0,1,8)	N/A
**NhaB**	NhaB sodium/proton transporter	b1186	1232399..1233940	-	Y	Y	Y	-6	AAGGGAAA(0,1,8)	N/A
**FadR**	fatty acid metabolism regulator	b1187	1234161..1234880	+	Y	Y	Y	-8	TATGGAAA(0,1,8)	N/A
**DadX**	alanine racemase	b1190	1238102..1239172	+	Y	Y	Y	-5	TAAGGAAA(0,1,8)	N/A
**LdcA**	L,D-carboxypeptidase A	b1192	1241389..1242303	-	Y	Y	Y	-5	CAAGGAAT(0,1,8)	N/A
**MltE**	murein transglycosylase E	b1193	1242403..1243014	+	Y	Y	Y	-7	TAAGGATA(0,1,8)	N/A
**TreA**	periplasmic trehalase	b1197	1244902..1246599	-	Y	Y	Y	-8	AAAGGAGA(0,1,8)	N/A
**DhaM**	fused predicted dihydroxyacetone-specific PTS enzymes: HPr component/EI component	b1198	1246919..1248337	-	Y	Y	Y	-3	TAAGGAAT(0,1,8)	N/A
**DhaL**	dihydroxyacetone kinase, C-terminal domain	b1199	1248348..1248980	-	Y	Y	Y	-6	TAAGGAGA(0,1,8)	N/A
**LolB**	outer membrane lipoprotein LolB precursor	b1209	1262100..1262723	-	Y	N	N	-11	CAAGGACT(0)	N/A
**ChaA**	calcium/sodium:proton antiporter	b1216	1269972..1271072	-	Y	Y	Y	-7	AAGGGATA(0,1,8)	N/A
**ChaB**	cation transport regulator	b1217	1271342..1271572	+	Y	Y	Y	-6	AAAGGAGT(0,1,8)	N/A
**YchJ**	hypothetical protein	b1233	1287897..1288355	-	Y	Y	Y	-7	CAGGGAGA(0,1,8)	N/A
**OppD**	oligopeptide transporter ATP-binding component	b1246	1302778..1303791	+	Y	Y	Y	-7	TAAGGAGT(0,1,8)	N/A
**Cls**	cardiolipin synthetase	b1249	1305209..1306669	-	Y	Y	Y	-9	AAAGGATT(0,1,8)	N/A
**YciI**	orf, hypothetical protein	b1251	1308593..1308889	-	Y	Y	Y	-8	CAAGGAGT(0,1,8)	N/A
**YciB**	intracellular septation protein A	b1254	1310375..1310914	-	Y	N	N	-7	TACGGAAT(0)	N/A
**YciC**	hypothetical protein	b1255	1310944..1311687	-	Y	Y	Y	-5	CAAGGAGA(0,1,8)	N/A
**YciN**	hypothetical protein	b1273	1328441..1328692	-	Y	Y	Y	-6	TAAGGAGT(0,1,8)	N/A
**PgpB**	phosphatidylglycerophosphatase B	b1278	1337354..1338118	+	Y	Y	Y	-5	AAAGGAGA(0,1,8)	N/A
**YmiB**	expressed protein	b4672	1344820..1344924	+	N	N	Y	-4	AAAGGAAC(8)	N/A
**YciW**	putative oxidoreductase	b1287	1347004..1348131	-	N	N	Y	-5	CAAGGAGC(8)	N/A
**SapF**	predicted antimicrobial peptide transporter subunit	b1290	1349852..1350658	-	Y	Y	Y	-10	CATGGAGA(0,1,8)	N/A
**SapC**	predicted antimicrobial peptide transporter subunit	b1292	1351652..1352542	-	Y	Y	Y	-4	TAAGGAAT(0,1,8)	N/A
**PuuR**	DNA-binding transcriptional repressor	b1299	1359935..1360492	+	Y	Y	Y	-8	TAAGGAAA(0,1,8)	N/A
**PspB**	phage shock protein B	b1305	1366825..1367049	+	Y	Y	Y	-5	TAAGGAGT(0,1,8)	N/A
**YcjM**	putative polysaccharide hydrolase	b1309	1368240..1369919	+	Y	N	N	-7	CACGGAAA(0)	N/A
**YcjN**	predicted sugar transporter subunit: periplasmic-binding component of ABC superfamily	b1310	1369933..1371225	+	N	N	Y	-6	AAAGGAAC(8)	N/A
**YcjQ**	predicted oxidoreductase, Zn-dependent and NAD(P)-binding	b1313	1372987..1374039	+	Y	Y	Y	-6	CAAGGAGT(0,1,8)	N/A
**B1314**	putative transient receptor potential locus	b1314	1374058..1374846	+	Y	Y	Y	-8	AAAGGAAT(0,1,8)	N/A
**OmpG**	outer membrane porin	b1319	1379971..1380876	+	N	N	Y	-5	CAAGGAAC(8)	N/A
**YcjG**	putative muconate cycloisomerase I (EC 5.5.-.-)	b1325	1386954..1387919	+	Y	Y	Y	-5	CAAGGAGT(0,1,8)	N/A
**YcjZ**	predicted DNA-binding transcriptional regulator	b1328	1390015..1390914	+	Y	Y	Y	-7	CATGGAGA(0,1,8)	N/A
**YnaJ**	predicted inner membrane protein	b1332	1395389..1395646	+	Y	Y	Y	-6	CAAGGATT(0,1,8)	N/A
**RecT**	Rac prophage; recombination and repair protein	b1349	1412008..1412817	-	Y	Y	Y	-7	TAAGGAAT(0,1,8)	N/A
**RacC**	Rac prophage; predicted protein	b1351	1415512..1415787	-	Y	Y	Y	-6	TAAGGAGA(0,1,8)	N/A
**YdaD**	orf, hypothetical protein	b1352	1416032..1416253	-	Y	Y	Y	-6	AATGGAGA(0,1,8)	N/A
**OmpN**	outer membrane pore protein N, non-specific	b1377	1433784..1434917	-	Y	Y	Y	-7	AAAGGATT(0,1,8)	N/A
**FeaB**	phenylacetaldehyde dehydrogenase	b1385	1445543..1447042	+	Y	Y	Y	-5	TAAGGAAA(0,1,8)	N/A
**PaaB**	predicted multicomponent oxygenase/reductase subunit for phenylacetic acid degradation	b1389	1452892..1453179	+	Y	Y	Y	-7	TAAGGAGA(0,1,8)	N/A
**YdbD**	orf, hypothetical protein	b1407	1473168..1475474	+	Y	Y	Y	-5	CATGGAGT(0,1,8)	N/A
**B1408**	probable enzyme	b1408	1475645..1476250	+	Y	Y	Y	-5	CAAGGATT(0,1,8)	N/A
**AzoR**	acyl carrier protein phosphodiesterase	b1412	1480279..1480884	-	Y	Y	Y	-5	TAAGGAAA(0,1,8)	N/A
**YdcF**	hypothetical protein	b1414	1485259..1486059	+	N	N	Y	-15	AAGGGAAC(8)	N/A
**HokB**	toxic polypeptide, small	b4428	1489946..1490095	-	Y	Y	Y	-7	CAAGGAGA(0,1,8)	N/A
**YdcH**	orf, hypothetical protein	b1426	1496675..1496899	+	Y	Y	Y	-5	CAGGGAGT(0,1,8)	N/A
**RimL**	ribosomal-protein-L7/L12-serine acetyltransferase	b1427	1496962..1497501	+	Y	Y	Y	-14	CATGGATT(0,1,8)	N/A
**YdcY**	hypothetical protein	b1446	1515672..1515905	+	Y	Y	Y	-5	TAAGGAGA(0,1,8)	N/A
**YncE**	hypothetical protein	b1452	1521331..1522392	+	Y	Y	Y	-5	AAGGGAGT(0,1,8)	N/A
**AnsP**	L-asparagine permease	b1453	1522505..1524004	-	Y	Y	Y	-4	CAGGGAGA(0,1,8)	N/A
**YdcD**	hypothetical protein	b1457	1527946..1528428	+	Y	Y	Y	-5	AAGGGAGT(0,1,8)	N/A
**YdcC**	hypothetical protein	b1460	1529840..1530976	+	Y	Y	Y	-6	TAAGGAGA(0,1,8)	N/A
**YdcE**	4-oxalocrotonate tautomerase	b1461	1531076..1531309	+	N	N	Y	-7	AAAGGAGC(8)	N/A
**NhoA**	N-hydroxyarylamine O-acetyltransferase	b1463	1532048..1532893	+	Y	Y	Y	-7	AAGGGAAA(0,1,8)	N/A
**YddL**	predicted lipoprotein	b1472	1543762..1544052	-	Y	Y	Y	-8	AAAGGATT(0,1,8)	N/A
**B1481**	orf, hypothetical protein	b1481	1554089..1554304	-	Y	N	N	-7	CACGGAGA(0)	N/A
**GadB**	glutamate decarboxylase B, PLP-dependent	b1493	1568669..1570069	-	Y	Y	Y	-6	TAAGGAGT(0,1,8)	N/A
**YddB**	predicted porin protein	b1495	1573271..1575643	-	Y	N	N	-10	TACGGAGA(0)	N/A
**YdeO**	predicted DNA-binding transcriptional acfivator	b1499	1580950..1581711	-	Y	Y	Y	-5	TAAGGAGA(0,1,8)	N/A
**YdeS**	predicted fimbrial-like adhesin protein	b1504	1586333..1586863	-	N	N	Y	-9	TAAGGAAC(8)	N/A
**N/A**	N/A	N/A	1592133..1596110	-	N	N	Y	-7	TATGGAGC(8)	N/A
**LsrB**	AI2 transporter	b1516	1603075..1604097	+	Y	Y	Y	-6	AAAGGATA(0,1,8)	N/A
**LsrG**	autoinducer-2 (AI-2) modifying protein LsrG	b1518	1605023..1605313	+	Y	Y	Y	-7	TAAGGAGA(0,1,8)	N/A
**UxaB**	tagaturonate reductase	b1521	1607253..1608704	-	N	N	Y	-6	AAGGGAAC(8)	N/A
**B1525**	putative aldehyde dehydrogenase	b1525	1611339..1612727	-	Y	Y	Y	-8	AAAGGAGA(0,1,8)	N/A
**YdeH**	hypothetical protein	b1535	1620984..1621874	-	Y	Y	Y	-5	AAAGGAGT(0,1,8)	N/A
**YdeI**	hypothetical protein	b1536	1622129..1622521	-	Y	Y	Y	-5	TAAGGAGT(0,1,8)	N/A
**YdfZ**	hypothetical protein	b1541	1627239..1627442	+	Y	Y	Y	-9	AAAGGAGA(0,1,8)	N/A
**YdfI**	predicted mannonate dehydrogenase	b1542	1627477..1628937	-	Y	Y	Y	-5	TAAGGAAT(0,1,8)	N/A
**CspB**	Qin prophage; cold shock protein	b1557	1639363..1639578	-	Y	Y	Y	-6	TAAGGAAT(0,1,8)	N/A
**CspF**	Qin prophage; cold shock protein	b1558	1639879..1640091	+	Y	Y	Y	-5	TAAGGAAT(0,1,8)	N/A
**FlxA**	Qin prophage; predicted protein	b1566	1644429..1644761	+	Y	Y	Y	-6	AAAGGAGA(0,1,8)	N/A
**RspB**	predicted oxidoreductase, Zn-dependent and NAD(P)-binding	b1580	1650920..1651939	-	Y	Y	Y	-7	TAAGGAGT(0,1,8)	N/A
**RspA**	predicted dehydratase	b1581	1651951..1653165	-	Y	Y	Y	-6	CAAGGAAT(0,1,8)	N/A
**YnfB**	hypothetical protein	b1583	1653832..1654173	+	Y	Y	Y	-6	CAGGGAAT(0,1,8)	N/A
**SpeG**	spermidine N1-acetyltransferase	b1584	1654208..1654768	+	Y	Y	Y	-5,-14	TAAGGACA(0),TAAGGAAT(0,1,8)	N/A
**B1586**	orf, hypothetical protein	b1586	1655589..1655894	+	N	N	Y	-6	TAAGGAGC(8)	N/A
**YnfE**	oxidoreductase subunit	b1587	1656093..1658519	+	Y	Y	Y	-6	CAGGGAGT(0,1,8)	N/A
**YnfG**	oxidoreductase, Fe-S subunit	b1589	1661014..1661631	+	Y	Y	Y	-6	TAAGGAGT(0,1,8)	N/A
**DgsA**	DNA-binding transcriptional repressor	b1594	1665368..1666588	-	Y	Y	Y	-6	TAGGGAGT(0,1,8)	N/A
**YnfM**	predicted transporter	b1596	1667723..1668976	+	Y	Y	Y	-5	CAAGGATT(0,1,8)	N/A
**FolM**	short chain dehydrogenase	b1606	1679000..1679722	+	Y	Y	Y	-6	AAAGGAGA(0,1,8)	N/A
**ManA**	mannose-6-phosphate isomerase	b1613	1686600..1687775	+	Y	Y	Y	-5	CAGGGATT(0,1,8)	N/A
**MalI**	DNA-binding transcriptional repressor	b1620	1696176..1697204	-	Y	Y	Y	-12	CAGGGAGT(0,1,8)	N/A

**MalY**	bifunctional beta-cystathionase, PLP-dependent/ regulator of maltose regulon	b1622	1698981..1700153	+	Y	Y	Y	-5	TAAGGATA(0,1,8)	N/A
**Nth**	DNA glycosylase and apyrimidinic (AP) lyase (endonuclease III)	b1633	1709547..1710182	+	Y	Y	Y	-6	CAGGGAAT(0,1,8)	N/A
**PdxY**	pyridoxine kinase	b1636	1713050..1713913	-	N	N	Y	-1	CAGGGAAC(8)	N/A
**SlyA**	transcriptional regulator for cryptic hemolysin	b1642	1718414..1718848	-	Y	Y	Y	-6	TAAGGAGA(0,1,8)	N/A
**YdhI**	predicted inner membrane protein	b1643	1719049..1719285	+	Y	Y	Y	-8	AAAGGAAT(0,1,8)	N/A
**YnhF**	hypothetical protein	b4602	1735480..1735569	-	Y	Y	Y	-8	AAAGGAGA(0,1,8)	N/A
**YdhQ**	hypothetical protein	b1664	1742895..1744151	-	Y	Y	Y	-6	AAAGGAAT(0,1,8)	N/A
**YdhU**	predicted cytochrome	b1670	1747587..1748372	-	Y	Y	Y	-5	AAAGGAAA(0,1,8)	N/A
**YdhV**	predicted oxidoreductase	b1673	1749752..1751854	-	Y	Y	Y	-6	AAAGGAAA(0,1,8)	N/A
**SufC**	cysteine desulfurase ATPase component	b1682	1759790..1760536	-	Y	Y	Y	-5	TAAGGAAT(0,1,8)	N/A
**YdiN**	predicted transporter	b1691	1770536..1771801	+	N	N	Y	-2	CAAGGAGC(8)	N/A
**YdiB**	quinate/shikimate 5-dehydrogenase, NAD(P)-binding	b1692	1771813..1772679	+	Y	Y	Y	-6	AAAGGAGT(0,1,8)	N/A
**YdiP**	predicted DNA-binding transcriptional regulator	b1696	1776414..1777325	-	Y	Y	Y	-13	CATGGAAT(0,1,8)	N/A
**YdiR**	predicted electron transfer flavoprotein, FAD-binding	b1698	1778425..1779363	+	Y	N	N	-8	TACGGAGA(0)	N/A
**YdiS**	predicted oxidoreductase with FAD/NAD(P)-binding domain	b1699	1779419..1780708	+	N	N	Y	-8	AAGGGAGC(8)	N/A
**YdiT**	predicted 4Fe-4S ferredoxin-type protein	b1700	1780705..1780998	+	N	N	Y	-10	AAGGGAGC(8)	N/A
**BtuC**	vtamin B12-transporter permease	b1711	1792196..1793176	-	Y	N	N	-3	AACGGATA(0)	N/A
**YdiY**	hypothetical protein	b1722	1803349..1804107	-	Y	N	N	-9	TACGGACT(0)	N/A
**YniB**	predicted inner membrane protein	b1726	1806721..1807257	-	Y	Y	Y	-5	CAAGGAGA(0,1,8)	N/A
**YniC**	predicted hydrolase	b1727	1807404..1808072	+	Y	Y	Y	-8	AAAGGAGA(0,1,8)	N/A
**KatE**	hydroperoxidase HPII(III) (catalase)	b1732	1811891..1814152	+	Y	Y	Y	-9	TAAGGAGA(0,1,8)	N/A
**ChbF**	cryptic phospho-beta-glucosidase, NAD(P)-binding	b1734	1815172..1816524	-	Y	Y	Y	-5	AAGGGAGA(0,1,8)	N/A
**ChbR**	DNA-binding transcriptional dual regulator	b1735	1816629..1817471	-	Y	Y	Y	-3	TAAGGAGT(0,1,8)	N/A
**B1754**	orf, hypothetical protein	b1754	1834097..1835263	+	Y	Y	Y	-5	TAAGGAGA(0,1,8)	N/A
**YnjD**	predicted transporter subunit: ATP-binding component of ABC superfamily	b1756	1836771..1837424	+	Y	N	N	-7	CAAGGACT(0)	N/A
**B1758**	putative cytochrome oxidase	b1758	1838807..1839427	-	Y	Y	Y	-5	TAAGGAGT(0,1,8)	N/A
**YnjH**	hypothetical protein	b1760	1839887..1840159	-	Y	Y	Y	-6	TAAGGAGT(0,1,8)	N/A
**B1762**	orf, hypothetical protein	b1762	1841855..1842895	-	Y	Y	Y	-4	CAGGGAAT(0,1,8)	N/A
**YdjB**	orf, hypothetical protein	b1768	1849911..1850552	+	Y	Y	Y	-6	TAAGGAGA(0,1,8)	N/A
**YeaD**	orf, hypothetical protein	b1780	1861874..1862758	+	Y	N	N	-6	AAAGGACT(0)	N/A
**MipA**	scaffolding protein for murein synthesizing machinery	b1782	1863750..1864496	-	Y	Y	Y	-9	TAAGGAAT(0,1,8)	N/A
**YeaL**	conserved inner membrane protein	b1789	1872376..1872822	+	Y	Y	Y	-6	CAAGGAAA(0,1,8)	N/A
**YeaO**	orf, hypothetical protein	b1792	1874933..1875280	+	Y	Y	Y	-10	AAGGGAGA(0,1,8)	N/A
**YeaY**	predicted lipoprotein	b1806	1887975..1888556	-	Y	Y	Y	-7	AAAGGAGT(0,1,8)	N/A
**YoaC**	predicted protein	b1810	1892157..1892456	+	Y	Y	Y	-6	TAAGGATA(0,1,8)	N/A
**YobG**	hypothetical protein	b1826	1906647..1906790	-	Y	N	N	-6	AACGGAGA(0)	N/A
**YebV**	orf, hypothetical protein	b1836	1919804..1920040	+	Y	Y	Y	-9	AATGGAGA(0,1,8)	N/A
**YobA**	hypothetical protein	b1841	1922619..1922993	-	Y	Y	Y	-8	AAAGGAAT(0,1,8)	N/A
**YebF**	predicted protein	b1847	1928058..1928414	-	Y	Y	Y	-6	TATGGAGA(0,1,8)	N/A
**YebB**	orf, hypothetical protein	b1862	1944275..1944877	+	Y	Y	Y	-7	AATGGAAT(0,1,8)	N/A
**RuvC**	Holliday junction resolvase	b1863	1944879..1945400	-	Y	N	N	-7	AACGGAGA(0)	N/A
**YecN**	orf, hypothetical protein	b1869	1950290..1950685	+	Y	Y	Y	-5	AAGGGAGT(0,1,8)	N/A
**CutC**	copper homeostasis protein	b1874	1956544..1957290	-	Y	Y	Y	-5	TAAGGAGT(0,1,8)	N/A
**YecT**	orf, hypothetical protein	b1877	1959996..1960484	+	Y	Y	Y	-7	TAAGGAAT(0,1,8)	N/A
**CheB**	chemotaxis-specific methylesterase	b1883	1965476..1966525	-	Y	Y	Y	-5	TAAGGATT(0,1,8)	N/A
**YecG**	universal stress protein	b1895	1977777..1978205	+	N	N	Y	-6	TAAGGAAC(8)	N/A
**AraH**	fused L-arabinose transporter subunits of ABC superfamily: membrane components	b4460	1980578..1981564	-	Y	Y	Y	-3	TAAGGAGA(0,1,8)	N/A
**AraG**	fused L-arabinose transporter subunits of ABC superfamily: ATP-binding components	b1900	1981579..1983093	-	Y	N	N	-5	CACGGAGA(0)	N/A
**YecI**	predicted ferritin-like protein	b1902	1984949..1985452	+	Y	Y	Y	-7	TAAGGATA(0,1,8)	N/A
**YecA**	conserved metal-binding protein	b1908	1988978..1989643	-	Y	Y	Y	-6	CAAGGAAA(0,1,8)	N/A
**FliD**	flagellar capping protein	b1924	2001896..2003302	+	Y	Y	Y	-5	AAAGGAAA(0,1,8)	N/A
**AmyA**	cytoplasmic alpha-amylase	b1927	2004180..2005667	+	Y	Y	Y	-7	AATGGAGT(0,1,8)	N/A
**YedF**	hypothetical protein	b1930	2007503..2007736	+	Y	Y	Y	-5	TAAGGAGA(0,1,8)	N/A
**YedL**	predicted acyltransferase	b1932	2008624..2009103	+	Y	N	N	-5	TACGGAAT(0)	N/A
**HchA**	chaperone protein HchA	b1967	2033859..2034710	+	Y	Y	Y	-6	TAAGGAAT(0,1,8)	N/A
**YedX**	hypothetical protein	b1970	2036980..2037393	+	Y	Y	Y	-6	CAAGGATA(0,1,8)	N/A
**Amn**	AMP nucleosidase	b1982	2053085..2054539	+	N	N	Y	-12	TATGGAAC(8)	N/A
**ErfK**	conserved protein with NAD(P)-binding Rossmann-fold domain	b1990	2060415..2061347	-	Y	N	N	-3	AAGGGACA(0)	N/A
**Flu**	CP4-44 prophage; antigen 43 (Ag43) phase-variable biofilm formation autotransporter	b2000	2069563..2072682	+	Y	Y	Y	-6	TAAGGAAA(0,1,8)	N/A
**B2001**	orf, hypothetical protein	b2001	2072803..2074335	+	Y	N	N	-5	CACGGAGT(0)	N/A
**YeeU**	CP4-44 prophage; antitoxin of the YeeV-YeeU toxin-antitoxin system	b2004	2075136..2075504	+	Y	Y	Y	-6	TAAGGATT(0,1,8)	N/A
**YeeA**	conserved inner membrane protein	b2008	2077557..2078615	-	Y	N	N	-7	TACGGAGT(0)	N/A
**SbcB**	exonuclease I	b2011	2080780..2082207	+	Y	N	N	-8	AACGGATT(0)	N/A
**YeeF**	putative amino acid/amine transport protein	b2014	2083728..2085086	-	Y	Y	Y	-7	AAAGGAGA(0,1,8)	N/A
**YoeB**	toxin of the YoeB-YefM toxin-antitoxin system	b4539	2087235..2087489	-	Y	N	N	-8,-14	AAAGGACA(0),AACGGAAA(0)	N/A
**HisH**	imidazole glycerol phosphate synthase subunit HisH	b2023	2092559..2093149	+	Y	Y	Y	-7	AAAGGAGT(0,1,8)	N/A
**WbbH**	O-antigen polymerase	b2035	2104084..2105250	-	Y	N	N	-2	TACGGATT(0)	N/A
**RfbA**	glucose-1-phosphate thymidylyltransferase	b2039	2108162..2109043	-	Y	Y	Y	-7	AAAGGAAT(0,1,8)	N/A
**RfbB**	dTDP-glucose 4,6 dehydratase, NAD(P)-binding	b2041	2110000..2111085	-	Y	Y	Y	-8	TATGGAAT(0,1,8)	N/A
**WcaF**	predicted acyl transferase	b2054	2126364..2126912	-	Y	Y	Y	-5	TAAGGAAA(0,1,8)	N/A
**Dcd**	deoxycytidine triphosphate deaminase	b2065	2139658..2140239	-	Y	Y	Y	-8	AAAGGAGA(0,1,8)	N/A
**AlkA**	3-methyl-adenine DNA glycosylase II	b2068	2144716..2145564	-	Y	Y	Y	-5	TAAGGAGA(0,1,8)	N/A
**YegJ**	hypothetical protein	b2071	2149209..2149670	+	Y	Y	Y	-12	AAGGGATT(0,1,8)	N/A
**YegL**	hypothetical protein	b2073	2150493..2151152	-	Y	Y	Y	-5	AAAGGATT(0,1,8)	N/A
**IbsA**	toxic membrane protein	b4667	2151373..2151432	-	Y	Y	Y	-6	TAAGGAAA(0,1,8)	N/A
**IbsB**	toxic membrane protein	b4668	2151705..2151761	-	Y	Y	Y	-6	TAAGGAAA(0,1,8)	N/A
**MdtB**	multidrug efflux system, subunit B	b2075	2153287..2156409	+	N	N	Y	-10	AAAGGAGC(8)	N/A
**BaeS**	sensory histidine kinase in two-component regulatory system with BaeR	b2078	2160900..2162303	+	N	N	Y	-6	AAAGGAGC(8)	N/A
**B2080**	orf, hypothetical protein	b2080	2163213..2163545	+	Y	Y	Y	-5	CAAGGAGT(0,1,8)	N/A
**GatB**	galactitol-specific enzyme IIB component of PTS	b2093	2172304..2172588	-	Y	N	N	-5	AAAGGACT(0)	N/A
**GatZ**	D-tagatose 1,6-bisphosphate aldolase 2, subunit	b2095	2173081..2174343	-	Y	Y	Y	-5	CAAGGAAA(0,1,8)	N/A
**YehD**	predicted fimbrial-like adhesin protein	b2111	2189702..2190244	-	N	N	Y	-6	TAAGGAAC(8)	N/A
**YehE**	hypothetical protein	b2112	2190537..2190818	-	Y	Y	Y	-8	CAGGGATA(0,1,8)	N/A
**Mrp**	putative ATPase	b2113	2191081..2192190	-	Y	Y	Y	-5	AAAGGAGT(0,1,8)	N/A
**YehI**	hypothetical protein	b2118	2198301..2201933	+	Y	Y	Y	-5	TAAGGAAA(0,1,8)	N/A
**YehR**	orf, hypothetical protein	b2123	2209247..2209708	+	Y	Y	Y	-6	AATGGAGT(0,1,8)	N/A
**YohO**	hypothetical protein	b4542	2213679..2213786	+	Y	Y	Y	-6	TAAGGAGT(0,1,8)	N/A
**Dld**	D-lactate dehydrogenase, FAD-binding, NADH independent	b2133	2220207..2221922	+	Y	Y	Y	-6	CAAGGAGT(0,1,8)	N/A
**YohD**	orf, hypothetical protein	b2136	2223823..2224401	+	Y	Y	Y	-12	AAGGGAGA(0,1,8)	N/A
**N/A**	N/A	N/A	2225345..2226433	-	N	N	Y	-9	TATGGAGC(8)	N/A
**YohP**	expressed protein	b4679	2227004..2227087	+	N	N	Y	-5	AAAGGAAC(8)	N/A
**YohK**	predicted inner membrane protein	b2142	2229041..2229736	+	N	N	Y	-7	AAAGGATC(8)	N/A
**YeiS**	predicted inner membrane protein	b2145	2231622..2231861	+	Y	Y	Y	-7	CAAGGAAA(0,1,8)	N/A
**YeiG**	predicted esterase	b2154	2241932..2242768	+	N	N	Y	-6	CAAGGAGC(8)	N/A
**CirA**	ferric iron-catecholate outer membrane transporter	b2155	2242800..2244791	-	Y	Y	Y	-7	CATGGAGA(0,1,8)	N/A
**YeiH**	conserved inner membrane protein	b2158	2247739..2248788	+	Y	Y	Y	-6	AAAGGAGA(0,1,8)	N/A
**YeiM**	predicted nucleoside transporter	b2164	2254107..2255357	-	Y	Y	Y	-7	AAGGGAAA(0,1,8)	N/A
**Spr**	predicted peptidase, outer membrane lipoprotein	b2175	2268001..2268567	+	Y	Y	Y	-10	AAGGGAAA(0,1,8)	N/A
**Rtn**	hypothetical protein	b2176	2268748..2270304	+	Y	N	N	-2	AACGGAAT(0)	N/A
**YejA**	orf, hypothetical protein	b2177	2270386..2272200	+	Y	Y	Y	-11	AAAGGAAA(0,1,8)	N/A
**YejB**	predicted oligopeptide transporter subunit	b2178	2272201..2273295	+	Y	Y	Y	-5	CAGGGAGA(0,1,8)	N/A
**YejK**	nucleoid-associated protein NdpA	b2186	2280962..2281969	-	Y	Y	Y	-8	AAAGGAGA(0,1,8)	N/A
**YejM**	predicted hydrolase, inner membrane	b2188	2282398..2284158	+	Y	Y	Y	-14	AAGGGAAA(0,1,8)	N/A
**Ada**	fused DNA-binding transcriptional dual regulator/O6-methylguanine-DNA methyltransferase	b2213	2307363..2308427	-	N	N	Y	-6	CAGGGAGC(8)	N/A
**AtoC**	fused response regulator of ato operon, in two-component system with AtoS: response regulator	b2220	2319888..2321273	+	Y	Y	Y	-10	CAGGGAAA(0,1,8)	N/A
**YfaP**	hypothetical protein	b2225	2325389..2326165	-	Y	N	N	-7	AATGGACT(0)	N/A
**YfaA**	orf, hypothetical protein	b2230	2332978..2334666	-	Y	Y	Y	-7	CAAGGAAT(0,1,8)	N/A
**UbiG**	3-demethylubiquinone-9 3-methyltransferase	b2232	2337589..2338311	+	Y	N	N	-11	TACGGAGT(0)	N/A
**YfaU**	predicted 2,4-dihydroxyhept-2-ene-1,7-dioic acid aldolase	b2245	2356064..2356867	-	N	N	Y	-4	TAAGGAAC(8)	N/A
**B2246**	putative transport protein	b2246	2356885..2358174	-	N	N	Y	-6	TAAGGAGC(8)	N/A
**YfaW**	predicted enolase	b2247	2358231..2359436	-	Y	Y	Y	-6	AATGGAGA(0,1,8)	N/A
**YfaO**	predicted NUDIX hydrolase	b2251	2362576..2363001	+	Y	Y	Y	-7	TAAGGAAT(0,1,8)	N/A
**YfbH**	hypothetical protein	b2256	2368040..2368930	+	Y	N	N	-8	TACGGATA(0)	N/A

**MenC**	O-succinylbenzoate synthase	b2261	2373022..2373984	-	Y	N	N	-6	AACGGAAT(0)	N/A
**MenB**	naphthoate synthase	b2262	2373984..2374841	-	Y	N	N	-5	AAAGGACA(0)	N/A
**MenF**	isochorismate hydroxymutase 2, menaquinone biosynthesis	b2265	2377370..2378665	-	Y	Y	Y	-8	CAGGGAGA(0,1,8)	N/A
**ElaB**	hypothetical protein	b2266	2378744..2379049	-	Y	Y	Y	-7	AATGGAGA(0,1,8)	N/A
**ElaA**	predicted acyltransferase with acyl-CoA N-acyltransferase domain	b2267	2379104..2379565	-	Y	Y	Y	-6	AAAGGAGA(0,1,8)	N/A
**ElaD**	predicted enzyme	b2269	2380735..2381946	+	N	N	Y	-3	AAAGGAGC(8)	N/A
**YfbK**	hypothetical protein	b2270	2382017..2383744	-	Y	Y	Y	-5	AAAGGATT(0,1,8)	N/A
**YfbO**	predicted protein	b2274	2386657..2387079	+	Y	Y	Y	-5	CAGGGAAT(0,1,8)	N/A
**NuoM**	NADH dehydrogenase subunit M	b2277	2389534..2391063	-	Y	Y	Y	-11	CAAGGAAT(0,1,8)	N/A
**NuoJ**	NADH dehydrogenase subunit J	b2280	2393364..2393918	-	Y	Y	Y	-7	TAAGGAGA(0,1,8)	N/A
**YfbS**	predicted transporter	b2292	2407542..2409374	-	Y	Y	Y	-5	CAAGGATA(0,1,8)	N/A
**YfcC**	putative S-transferase	b2298	2415103..2416623	+	Y	Y	Y	-10	AAGGGAAT(0,1,8)	N/A
**FolX**	D-erythro-7,8-dihydroneopterin triphosphate 2'-epimerase and dihydroneopterin aldolase	b2303	2419347..2419709	+	Y	N	N	-8	AACGGAGA(0)	N/A
**Flk**	predicted flagella assembly protein	b2321	2435972..2436967	+	Y	Y	Y	-5	TAAGGATA(0,1,8)	N/A
**YfcS**	predicted periplasmic pilus chaperone	b2336	2449606..2450358	-	Y	Y	Y	-6	TAAGGAAT(0,1,8)	N/A
**N/A**	N/A	N/A	2451287..2453023	-	Y	N	N	-10	AACGGATA(0)	N/A
**YfcV**	predicted fimbrial-like adhesin protein	b2339	2453105..2453668	-	Y	N	N	-5	TAAGGACA(0)	N/A
**YfdF**	hypothetical protein	b2345	2461034..2462092	+	Y	Y	Y	-7	AATGGAAT(0,1,8)	N/A
**VacJ**	predicted lipoprotein	b2346	2462274..2463029	-	Y	Y	Y	-6	CAGGGAGA(0,1,8)	N/A
**YfdK**	CPS-53 (KpLE1) prophage; conserved protein	b2354	2469099..2469539	-	Y	Y	Y	-6	TATGGAGT(0,1,8)	N/A
**YfdM**	orf, hypothetical protein	b2356	2470134..2470409	-	Y	Y	Y	-15	CAGGGAAT(0,1,8)	N/A
**EmrY**	predicted multidrug efflux system	b2367	2478660..2480198	-	Y	N	N	-7	AATGGACA(0)	N/A
**EvgA**	DNA-binding response regulator in two-component regulatory system with EvgS	b2369	2481777..2482391	+	Y	Y	Y	-6	AAGGGAAT(0,1,8)	N/A
**YpdG**	predicted enzyme IIC component of PTS	b2386	2504669..2505916	-	Y	Y	Y	-7	CAGGGATA(0,1,8)	N/A
**YfeO**	hypothetical protein	b2389	2507652..2508908	+	Y	Y	Y	-6	TAAGGAAT(0,1,8)	N/A
**YfeC**	orf, hypothetical protein	b2398	2516489..2516833	+	Y	Y	Y	-3	CAAGGAGA(0,1,8)	N/A
**XapR**	DNA-binding transcriptional activator	b2405	2519615..2520499	-	N	N	Y	-9	TATGGAGC(8)	N/A
**XapB**	xanthosine transporter	b2406	2520751..2522007	-	Y	Y	Y	-6	CAAGGAAA(0,1,8)	N/A
**XapA**	purine nucleoside phosphorylase	b2407	2522067..2522900	-	Y	Y	Y	-4	AAAGGATA(0,1,8)	N/A
**YfeN**	conserved outer membrane protein	b2408	2523149..2523913	+	Y	Y	Y	-7	TAGGGAAT(0,1,8)	N/A
**CysZ**	putative sulfate transport protein CysZ	b2413	2529485..2530246	+	N	N	Y	-12	AAAGGAGC(8)	N/A
**PdxK**	pyridoxine kinase	b2418	2534408..2535259	-	Y	Y	Y	-10	CAAGGAGA(0,1,8)	N/A
**YfeK**	hypothetical protein	b2419	2535364..2535738	+	Y	Y	Y	-11	CAAGGATA(0,1,8)	N/A
**UcpA**	putative oxidoreductase	b2426	2541854..2542645	-	Y	Y	Y	-9	CAAGGAGA(0,1,8)	N/A
**MurP**	N-acetylmuramic acid phosphotransfer permease	b2429	2544695..2546119	+	Y	Y	Y	-6	CAAGGAAT(0,1,8)	N/A
**EutH**	predicted inner membrane protein	b2452	2564903..2566129	-	Y	Y	Y	-8	CAGGGAGT(0,1,8)	N/A
**EutJ**	predicted chaperonin, ethanolamine utilization protein	b2454	2567523..2568359	-	Y	Y	Y	-6	TAAGGAGT(0,1,8)	N/A
**TalA**	transaldolase A	b2464	2576688..2577638	+	Y	Y	Y	-8	CAAGGAGT(0,1,8)	N/A
**TktB**	transketolase 2, thiamin-binding	b2465	2577658..2579661	+	Y	N	N	-6	CACGGAGT(0)	N/A
**YffB**	hypothetical protein	b2471	2589269..2589625	+	Y	Y	Y	-5	AAAGGATT(0,1,8)	N/A
**YpfH**	orf, hypothetical protein	b2473	2591094..2591792	-	Y	Y	Y	-6	TAAGGAGT(0,1,8)	N/A
**GcvR**	transcriptional regulation of gcv operon	b2479	2597928..2598500	+	Y	Y	Y	-8	AAAGGAAT(0,1,8)	N/A
**HyfA**	hydrogenase 4 Fe-S subunit	b2481	2599223..2599840	+	N	N	Y	-5	CAAGGAGC(8)	N/A
**HyfC**	hydrogenase 4 membrane subunit	b2483	2601869..2602816	+	Y	Y	Y	-6	TAAGGAAA(0,1,8)	N/A
**HyfD**	hydrogenase 4 membrane subunit	b2484	2602833..2604272	+	N	N	Y	-12	TAAGGAGC(8)	N/A
**HyfE**	hydrogenase 4, membrane subunit	b2485	2604284..2604934	+	Y	Y	Y	-7	TAAGGAGA(0,1,8)	N/A
**HyfF**	NADH dehydrogenase subunit N	b2486	2604939..2606519	+	Y	Y	Y	-6	AAGGGATA(0,1,8)	N/A
**HyfH**	hydrogenase 4, Fe-S subunit	b2488	2608186..2608731	+	Y	N	N	-5	TAAGGACA(0)	N/A
**HyfI**	hydrogenase 4, Fe-S subunit	b2489	2608728..2609486	+	N	N	Y	-5	TAAGGAGC(8)	N/A
**HyfR**	putative 2-component regulator, interaction with sigma 54	b2491	2609922..2611934	+	Y	N	N	-5	TAAGGACT(0)	N/A
**Ppk**	polyphosphate kinase	b2501	2621066..2623132	+	Y	N	N	-11	AACGGAGT(0)	N/A
**YfgG**	hypothetical protein	b2504	2627312..2627503	+	N	N	Y	-6	CAAGGAAC(8)	N/A
**YfgH**	predicted outer membrane lipoprotein	b2505	2627814..2628332	+	Y	Y	Y	-2	CAAGGAAT(0,1,8)	N/A
**YfhR**	predicted peptidase	b2534	2662412..2663266	+	Y	Y	Y	-5	CAAGGAAA(0,1,8)	N/A
**YphA**	predicted inner membrane protein	b2543	2671368..2671790	+	Y	Y	Y	-5	AAGGGATT(0,1,8)	N/A
**YphB**	hypothetical protein	b2544	2671838..2672710	-	Y	Y	Y	-7	TAAGGAGT(0,1,8)	N/A
**GlnB**	regulatory protein P-II for glutamine synthetase	b2553	2685092..2685430	-	Y	Y	Y	-4	CAAGGAAT(0,1,8)	N/A
**ShoB**	toxic membrane protein	b4687	2698139..2698219	-	Y	Y	Y	-6	TAAGGAAA(0,1,8)	N/A
**PheL**	pheA gene leader peptide	b2598	2735621..2735668	+	Y	Y	Y	-6	TAAGGAAA(0,1,8)	N/A
**YfiL**	orf, hypothetical protein	b2602	2739382..2739747	+	Y	Y	Y	-6	AAAGGAGA(0,1,8)	N/A
**YfiB**	predicted outer membrane lipoprotein	b2605	2741647..2742129	+	Y	Y	Y	-8	CAAGGATA(0,1,8)	N/A
**YfjD**	predicted inner membrane protein	b4461	2746796..2748082	+	Y	Y	Y	-6	AAAGGAGT(0,1,8)	N/A
**SmpA**	small membrane protein A	b2617	2751627..2751968	+	Y	Y	Y	-10	AAAGGAAT(0,1,8)	N/A
**IntA**	CP4-57 prophage; integrase	b2622	2754181..2755422	+	Y	Y	Y	-7	CAGGGAGA(0,1,8)	N/A
**YfjO**	orf, hypothetical protein	b2631	2765006..2765377	+	Y	N	N	-7	CAAGGACT(0)	N/A
**B2636**	orf, hypothetical protein	b2636	2768703..2769146	+	Y	Y	Y	-6	CAGGGAAA(0,1,8)	N/A
**YfjT**	CP4-57 prophage; predicted protein	b2637	2769170..2769637	+	Y	Y	Y	-6	AAAGGAGA(0,1,8)	N/A
**YfjW**	CP4-57 prophage; predicted inner membrane protein	b2642	2771340..2773043	+	Y	Y	Y	-5	CATGGATT(0,1,8)	N/A
**YfjX**	CP4-57 prophage; predicted antirestriction protein	b2643	2773941..2774399	+	Y	N	N	-8	CACGGAGA(0)	N/A
**YfjY**	CP4-57 prophage; predicted DNA repair protein	b2644	2774408..2774890	+	Y	Y	Y	-4	TAAGGATA(0,1,8)	N/A
**YgaF**	orf, hypothetical protein	b2660	2788004..2789272	+	Y	Y	Y	-8	AAAGGAAT(0,1,8)	N/A
**YqaE**	predicted membrane protein	b2666	2794892..2795050	-	Y	Y	Y	-5	TAGGGAGT(0,1,8)	N/A
**YgaP**	predicted inner membrane protein with hydrolase activity	b2668	2795542..2796066	+	Y	Y	Y	-5	TAAGGAGT(0,1,8)	N/A
**YgaW**	predicted inner membrane protein	b2670	2797186..2797635	+	N	N	Y	-7	TAAGGAGC(8)	N/A
**NrdH**	glutaredoxin-like protein	b2673	2798745..2798990	+	Y	N	N	-9	AACGGAAA(0)	N/A
**NrdE**	ribonucleotide-diphosphate reductase alpha subunit	b2675	2799370..2801514	+	Y	Y	Y	-10	AAAGGAGT(0,1,8)	N/A
**NrdF**	ribonucleotide-diphosphate reductase beta subunit	b2676	2801524..2802483	+	Y	Y	Y	-5	TAAGGAAT(0,1,8)	N/A
**ProV**	glycine betaine transporter subunit	b2677	2802837..2804039	+	Y	Y	Y	-12	AAAGGAAT(0,1,8)	N/A
**ProX**	glycine betaine transporter subunit	b2679	2805154..2806146	+	Y	Y	Y	-5	AAAGGAAT(0,1,8)	N/A
**YgaH**	predicted inner membrane protein	b2683	2808366..2808701	+	N	N	Y	-6	CAAGGAGC(8)	N/A
**CsrA**	carbon storage regulator	b2696	2816983..2817168	-	N	N	Y	-6	CAAGGAGC(8)	N/A
**RecX**	RecA regulator RecX	b2698	2820161..2820661	-	Y	Y	Y	-5	TAAGGATA(0,1,8)	N/A
**SrlD**	3-ketoacyl-(acyl-carrier-protein) reductase	b2705	2825759..2826538	+	Y	Y	Y	-6	TAAGGAGT(0,1,8)	N/A
**GutM**	DNA-binding transcriptional activator of glucitol operon	b2706	2826643..2827002	+	Y	Y	Y	-5	TAAGGAAA(0,1,8)	N/A
**HycF**	formate hydrogenlyase complex iron-sulfur protein	b2720	2842232..2842774	-	Y	Y	Y	-5	TAAGGAAT(0,1,8)	N/A
**MutS**	DNA mismatch repair protein	b2733	2855115..2857676	+	N	N	Y	-14	CAGGGAAC(8)	N/A
**YgbJ**	predicted dehydrogenase, with NAD(P)-binding Rossmann-fold domain	b2736	2859452..2860360	+	Y	Y	Y	-5	CAAGGAGA(0,1,8)	N/A
**YgbM**	hypothetical protein	b2739	2862258..2863034	+	Y	Y	Y	-8	TAAGGAGT(0,1,8)	N/A
**Iap**	aminopeptidase in alkaline phosphatase isozyme conversion	b2753	2874603..2875640	+	Y	N	N	-5	TAAGGACT(0)	N/A
**YgcJ**	hypothetical protein	b2758	2879073..2880164	-	Y	Y	Y	-8	TAAGGAAA(0,1,8)	N/A
**YgcP**	predicted anti-terminator regulatory protein	b2768	2892218..2892793	+	Y	Y	Y	-5	TAAGGATA(0,1,8)	N/A
**YgcW**	putative oxidoreductase	b2774	2897510..2898295	-	Y	N	N	-13	TACGGAAA(0)	N/A
**YqcG**	expressed protein	b4682	2903579..2903719	+	Y	Y	Y	-9	AAGGGAGT(0,1,8)	N/A
**RelA**	(p)ppGpp synthetase I/GTP pyrophosphokinase	b2784	2909439..2911673	-	Y	Y	Y	-6	AAAGGAGA(0,1,8)	N/A
**GudD**	(D)-glucarate dehydratase 1	b2787	2916067..2917407	-	Y	N	N	-6	AAAGGACA(0)	N/A
**YqcA**	flavodoxin	b2790	2920557..2921006	-	Y	Y	Y	-8	CAGGGAGA(0,1,8)	N/A
**YgdH**	hypothetical protein	b2795	2924330..2925694	+	Y	Y	Y	-6	TAAGGAGT(0,1,8)	N/A
**FucO**	L-1,2-propanediol oxidoreductase	b2799	2929887..2931038	-	Y	Y	Y	-4	CAAGGAGA(0,1,8)	N/A
**B2809**	orf, hypothetical protein	b2809	2940940..2941167	-	Y	Y	Y	-6	TAAGGAGA(0,1,8)	N/A
**PpdB**	hypothetical protein	b2825	2961175..2961738	-	Y	Y	Y	-10	CAAGGAGA(0,1,8)	N/A
**Aas**	2-acyl-glycerophospho-ethanolamine acyltransferase	b2836	2971877..2974036	-	Y	Y	Y	-5	AAGGGAGT(0,1,8)	N/A
**YqeH**	orf, hypothetical protein	b2846	2985558..2986190	+	Y	N	N	-15	TACGGATA(0)	N/A
**YqeL**	expressed protein	b4683	2987841..2987921	-	Y	Y	Y	-9	CAAGGAGA(0,1,8)	N/A
**YgeI**	hypothetical protein	b2853	2991660..2991878	+	N	N	Y	-4	AAGGGAGC(8)	N/A
**YgeX**	diaminopropionate ammonia-lyase	b2871	3005532..3006728	+	Y	Y	Y	-5	AAAGGATA(0,1,8)	N/A
**YgfJ**	hypothetical protein	b2877	3013182..3013760	+	N	N	Y	-5	AATGGAAC(8)	N/A
**YgfK**	predicted oxidoreductase, Fe-S subunit	b2878	3014082..3017180	+	Y	Y	Y	-6	TAAGGAGA(0,1,8)	N/A
**YgfM**	predicted oxidoreductase	b2880	3018562..3019341	+	Y	Y	Y	-5	TAAGGAAA(0,1,8)	N/A
**YgfO**	putative transport protein	b2882	3022373..3023773	+	Y	Y	Y	-7	AAAGGAGT(0,1,8)	N/A
**GuaD**	guanine deaminase	b2883	3023788..3025107	+	Y	Y	Y	-3	AAAGGAGT(0,1,8)	N/A
**YgfU**	putative permease	b2888	3029389..3030837	+	Y	Y	Y	-8	AAAGGAGA(0,1,8)	N/A
**YqfG**	expressed protein	b4684	3030839..3030964	+	Y	Y	Y	-6	CATGGAGA(0,1,8)	N/A
**VisC**	hypothetical protein	b2906	3049137..3050339	-	Y	Y	Y	-6	TAAGGAAT(0,1,8)	N/A

**YqfE**	hypothetical protein	b2915	3057403..3057633	-	Y	Y	Y	-8	CAGGGATT(0,1,8)	N/A
**YgfG**	putative enzyme	b2919	3062015..3062800	+	Y	Y	Y	-5	AAAGGAAT(0,1,8)	N/A
**YggE**	hypothetical protein	b2922	3065362..3066102	-	Y	N	N	-8	CACGGAGA(0)	N/A
**YggC**	hypothetical protein	b2928	3071998..3072711	-	Y	Y	Y	-8	AAAGGAAT(0,1,8)	N/A
**CmtB**	predicted mannitol-specific enzyme IIA component of PTS	b2934	3076909..3077352	-	Y	Y	Y	-5	TAAGGATA(0,1,8)	N/A
**EndA**	DNA-specific endonuclease I	b2945	3088369..3089076	+	Y	N	N	-6	CACGGAGT(0)	N/A
**YggT**	predicted inner membrane protein	b2952	3093842..3094408	+	Y	Y	Y	-4,-13	AAAGGAAC(8),TAAGGAAT(0,1,8)	N/A
**GlcG**	hypothetical protein	b2977	3121849..3122253	-	Y	Y	Y	-7	AAAGGAAT(0,1,8)	N/A
**YghT**	predicted protein with nucleoside triphosphate hydrolase domain	b2986	3132153..3132845	+	Y	Y	Y	-6	AAAGGATA(0,1,8)	N/A
**HybO**	hydrogenase 2, small subunit	b2997	3143165..3144283	-	Y	N	N	-9	AACGGAAT(0)	N/A
**YghZ**	aldo-keto reductase	b3001	3145919..3146959	+	Y	Y	Y	-5	AAAGGAGA(0,1,8)	N/A
**YghA**	oxidoreductase	b3003	3147684..3148568	+	Y	Y	Y	-7	AAGGGAGA(0,1,8)	N/A
**YqhD**	alcohol dehydrogenase, NAD(P)-dependent	b3011	3153377..3154540	+	N	N	Y	-6	AAGGGAGC(8)	N/A
**YqhG**	hypothetical protein	b3013	3155672..3156598	+	Y	N	N	-13	AACGGAGA(0)	N/A
**YqhH**	predicted outer membrane lipoprotein	b3014	3156649..3156906	+	N	N	Y	-6	AAGGGAAC(8)	N/A
**SufI**	repressor protein for FtsI	b3017	3159279..3160691	-	N	N	Y	-5	CATGGAGC(8)	N/A
**YgiS**	predicted transporter subunit: periplasmic-binding component of ABC superfamily	b3020	3164133..3165740	-	Y	Y	Y	-4	TAAGGAAT(0,1,8)	N/A
**YgiT**	predicted DNA-binding transcriptional regulator	b3021	3165873..3166268	-	Y	Y	Y	-7	TAAGGAGA(0,1,8)	N/A
**YgiW**	hypothetical protein	b3024	3167306..3167698	-	Y	Y	Y	-8	AAGGGAGT(0,1,8)	N/A
**CpdA**	cyclic 3',5'-adenosine monophosphate phosphodiesterase	b3032	3174028..3174855	-	Y	N	N	-5	TAAGGACA(0)	N/A
**YgiD**	predicted dioxygenase	b3039	3179641..3180429	-	Y	Y	Y	-6	TAAGGATA(0,1,8)	N/A
**YgiE**	zinc transporter ZupT	b3040	3180572..3181345	+	Y	Y	Y	-6	TAAGGAGT(0,1,8)	N/A
**B3042**	orf, hypothetical protein	b3042	3182862..3183152	+	Y	Y	Y	-6	CAGGGAAA(0,1,8)	N/A
**YgiL**	predicted fimbrial-like adhesin protein	b3043	3183436..3183987	+	Y	Y	Y	-4	AAAGGAAT(0,1,8)	N/A
**YqiJ**	predicted inner membrane protein	b3050	3190230..3190859	+	Y	N	N	-13	AATGGACA(0)	N/A
**YqiK**	hypothetical protein	b3051	3190886..3192547	+	Y	Y	Y	-8	AAAGGAAA(0,1,8)	N/A
**IbsE**	toxic membrane protein	b4666	3193163..3193222	+	Y	Y	Y	-6	TAAGGAAA(0,1,8)	N/A
**GlnE**	fused deadenylyltransferase/adenylyltransferase for glutamine synthetase	b3053	3194823..3197663	-	Y	Y	Y	-10	CAAGGATA(0,1,8)	N/A
**YgjF**	G/U mismatch-specific DNA glycosylase	b3068	3212989..3213495	-	Y	Y	Y	-5	AAAGGAGT(0,1,8)	N/A
**YgjJ**	hypothetical protein	b3079	3225823..3226893	+	Y	Y	Y	-7	CATGGATT(0,1,8)	N/A
**YgjK**	predicted glycosyl hydrolase	b3080	3226910..3229261	+	Y	N	N	-9	TAAGGACT(0)	N/A
**YgjQ**	predicted thioredoxin-like	b3086	3234562..3235254	+	Y	Y	Y	-7	TAAGGATA(0,1,8)	N/A
**YgjR**	predicted NAD(P)-binding dehydrogenase	b3087	3235333..3236319	+	Y	Y	Y	-12	TATGGAGT(0,1,8)	N/A
**Alx**	predicted inner membrane protein, part of terminus	b3088	3236602..3237567	+	N	N	Y	-5	TAAGGAAC(8)	N/A
**SstT**	sodium:serine/threonine symporter	b3089	3237966..3239210	+	N	N	Y	-6	AAAGGATC(8)	N/A
**YgjV**	conserved inner membrane protein	b3090	3239215..3239766	-	Y	Y	Y	-5	TAAGGATT(0,1,8)	N/A
**YqjB**	hypothetical protein	b3096	3246461..3246844	+	Y	Y	Y	-14	TATGGAAA(0,1,8)	N/A
**YhaL**	orf, hypothetical protein	b3107	3253065..3253229	+	Y	Y	Y	-6	CAGGGAGA(0,1,8)	N/A
**YhaC**	hypothetical protein	b3121	3266437..3267624	+	Y	Y	Y	-6	AAAGGATA(0,1,8)	N/A
**SohA**	predicted regulator	b3129	3275024..3275359	+	Y	N	N	-8	AAAGGACA(0)	N/A
**AgaR**	DNA-binding transcriptional dual regulator	b3131	3275878..3276687	-	Y	N	N	-5	AAAGGACT(0)	N/A
**AgaS**	tagatose-6-phosphate ketose/aldose isomerase	b3136	3279998..3281152	+	Y	Y	Y	-6	TAAGGATT(0,1,8)	N/A
**YraH**	predicted fimbrial-like adhesin protein	b3142	3285448..3286032	+	Y	Y	Y	-10	CAAGGATA(0,1,8)	N/A
**YraK**	predicted fimbrial-like adhesin protein	b3145	3289363..3290454	+	Y	Y	Y	-6	TAAGGATA(0,1,8)	N/A
**YraO**	DnaA initiator-associating factor for replication initiation	b3149	3293831..3294421	+	Y	Y	Y	-4	TAAGGATT(0,1,8)	N/A
**YraP**	hypothetical protein	b3150	3294431..3295006	+	Y	Y	Y	-5	TAAGGAGA(0,1,8)	N/A
**YraR**	orf, hypothetical protein	b3152	3296233..3296868	-	N	N	Y	-5	AAAGGAGC(8)	N/A
**YhbT**	predicted lipid carrier protein	b3157	3298774..3299298	-	Y	Y	Y	-8	CAGGGAGT(0,1,8)	N/A
**SecG**	protein-export membrane protein	b3175	3320195..3320527	-	N	N	Y	-10	CAAGGAAC(8)	N/A
**FolP**	7,8-dihydropteroate synthase	b3177	3322085..3322933	-	Y	Y	Y	-6	CAGGGATA(0,1,8)	N/A
**ObgE**	GTPase involved in cell partioning and DNA repair	b3183	3328604..3329776	-	Y	N	N	-6	TACGGAGA(0)	N/A
**YhbE**	conserved inner membrane protein	b3184	3329792..3330757	-	Y	Y	Y	-6	CAGGGAAT(0,1,8)	N/A
**SfsB**	DNA-binding transcriptional activator of maltose metabolism	b3188	3332931..3333209	+	Y	Y	Y	-9	TAAGGAGT(0,1,8)	N/A
**YrbE**	predicted toluene transporter subunit: membrane component of ABC superfamily	b3194	3336488..3337270	-	Y	Y	Y	-11	CAGGGAGT(0,1,8)	N/A
**KdsC**	3-deoxy-D-manno-octulosonate 8-phosphate phosphatase	b3198	3340295..3340861	+	Y	Y	Y	-8	CAAGGATA(0,1,8)	N/A
**GltF**	periplasmic protein	b3214	3359198..3359962	+	Y	Y	Y	-8	TAAGGATA(0,1,8)	N/A
**YhcG**	hypothetical protein	b3220	3365849..3366976	+	Y	N	N	-5	AAGGGACA(0)	N/A
**YhcH**	hypothetical protein	b3221	3367036..3367500	-	Y	Y	Y	-7	CAGGGAGA(0,1,8)	N/A
**DcuD**	predicted transporter	b3227	3372891..3374258	+	Y	N	N	-7	CACGGAGT(0)	N/A
**YhcM**	conserved protein with nucleoside triphosphate hydrolase domain	b3232	3376892..3378019	-	Y	Y	Y	-13	CAAGGAAT(0,1,8)	N/A
**YhdP**	conserved membrane protein, predicted transporter	b4472	3390480..3394280	-	Y	Y	Y	-6	CAAGGAGT(0,1,8)	N/A
**CafA**	bundles of cytoplasmic filaments	b3247	3394348..3395817	-	Y	Y	Y	-4	AAGGGATA(0,1,8)	N/A
**YhdT**	conserved inner membrane protein	b3257	3405397..3405639	+	Y	N	N	-5	CAGGGACA(0)	N/A
**YhdJ**	putative methyltransferase	b3262	3409675..3410559	+	Y	Y	Y	-5	AAGGGAGT(0,1,8)	N/A
**YhdY**	putative transport system permease protein	b3270	3419347..3420450	+	Y	Y	Y	-5	TAAGGAGT(0,1,8)	N/A
**Smf**	hypothetical protein	b4473	3430458..3431582	-	Y	Y	Y	-6	CAGGGAGA(0,1,8)	N/A
**Fmt**	methionyl-tRNA formyltransferase	b3288	3432236..3433183	+	Y	Y	Y	-10	TAAGGATA(0,1,8)	N/A
**MscL**	large-conductance mechanosensitive channel	b3291	3436046..3436456	+	Y	Y	Y	-6	TAGGGAGA(0,1,8)	N/A
**RpmJ**	50S ribosomal protein L36	b3299	3440640..3440756	-	Y	N	N	-7	TACGGAGA(0)	N/A
**RplX**	50S ribosomal protein L24	b3309	3445475..3445789	-	N	N	Y	-6	TAAGGAGC(8)	N/A
**YheF**	putative general protein secretion protein	b3325	3454399..3456351	+	Y	N	N	-6	AATGGACT(0)	N/A
**GspG**	pseudopilin, cryptic, general secretion pathway	b3328	3459045..3459482	+	Y	Y	Y	-5	TAAGGAAA(0,1,8)	N/A
**YheK**	putative general protein secretion protein	b3333	3461944..3463107	+	N	N	Y	-4	TAAGGATC(8)	N/A
**PshM**	putative general secretion	b3334	3463104..3463565	+	N	N	Y	-9	TAAGGAAC(8)	N/A
**GspO**	bifunctional prepilin leader peptidase/ methylase	b3335	3463565..3464242	+	N	N	Y	-8	CAGGGAGC(8)	N/A
**ChiA**	periplasmic endochitinase	b3338	3465182..3467875	-	Y	Y	Y	-4	AAGGGATT(0,1,8)	N/A
**TufA**	protein chain elongation factor EF-Tu (duplicate of tufB)	b3339	3468167..3469351	-	Y	Y	Y	-7	TAAGGAAT(0,1,8)	N/A
**YheO**	orf, hypothetical protein	b3346	3473740..3474462	-	Y	Y	Y	-11	AAAGGAGT(0,1,8)	N/A
**PrkB**	predicted phosphoribulokinase	b3355	3482512..3483381	+	Y	Y	Y	-5	CATGGAGT(0,1,8)	N/A
**PpiA**	peptidyl-prolyl cis-trans isomerase A (rotamase A)	b3363	3489747..3490319	-	Y	Y	Y	-5	TAAGGAAA(0,1,8)	N/A
**NirC**	nitrite transporter	b3367	3495025..3495831	+	Y	Y	Y	-6	AAAGGATA(0,1,8)	N/A
**YhfL**	conserved secreted peptide	b3369	3497470..3497637	+	Y	Y	Y	-5	TAGGGAAT(0,1,8)	N/A
**FrlC**	predicted isomerase	b4474	3500362..3501192	+	Y	Y	Y	-7	CAAGGAGT(0,1,8)	N/A
**YhfU**	orf, hypothetical protein	b3378	3505370..3505723	-	Y	N	N	-6	TAAGGACA(0)	N/A
**Rpe**	ribulose-phosphate 3-epimerase	b3386	3512404..3513081	-	Y	Y	Y	-6	CAAGGAGA(0,1,8)	N/A
**HofQ**	predicted fimbrial transporter	b3391	3517487..3518725	-	Y	Y	Y	-6	CAAGGAGA(0,1,8)	N/A
**YrfD**	orf, hypothetical protein	b3395	3519994..3520773	-	Y	Y	Y	-9	TAAGGAGA(0,1,8)	N/A
**GreB**	transcription elongation factor GreB	b3406	3534834..3535310	+	Y	Y	Y	-8	AAGGGAAT(0,1,8)	N/A
**RtcA**	RNA 3'-terminal-phosphate cyclase	b4475	3553855..3554871	-	Y	Y	Y	-5	AAAGGATA(0,1,8)	N/A
**RtcR**	sigma 54-dependent transcriptional regulator of rtcBA expression	b3422	3556290..3557888	+	Y	Y	Y	-7	TAAGGATA(0,1,8)	N/A
**GlpR**	DNA-binding transcriptional repressor	b3423	3557870..3558628	-	Y	Y	Y	-6	CAGGGATT(0,1,8)	N/A
**YzgL**	orf, hypothetical protein	b3427	3561747..3562028	-	Y	Y	Y	-6	AATGGAAA(0,1,8)	N/A
**YhgN**	predicted antibiotic transporter	b3434	3573094..3573687	+	Y	Y	Y	-8	CAGGGAAT(0,1,8)	N/A
**YhhZ**	hypothetical protein	b3442	3579886..3581064	+	Y	N	N	-9	AAAGGACT(0)	N/A
**LivG**	leucine/isoleucine/valine transporter subunit	b3455	3591462..3592229	-	Y	Y	Y	-7	AAAGGAGA(0,1,8)	N/A
**YhhL**	conserved inner membrane protein	b3466	3603002..3603271	+	Y	Y	Y	-6	CAAGGAGA(0,1,8)	N/A
**YhhN**	conserved inner membrane protein	b3468	3603774..3604400	+	N	N	Y	-5	CAAGGAAC(8)	N/A
**NikE**	nickel transporter subunit	b3480	3615799..3616605	+	N	N	Y	-8	TATGGAGC(8)	N/A
**RhsB**	rhsB element core protein RshB	b3482	3617215..3621450	+	Y	Y	Y	-6	AAAGGATT(0,1,8)	N/A
**YhhH**	orf, hypothetical protein	b3483	3621422..3621805	+	Y	Y	Y	-6	AAAGGATT(0,1,8)	N/A
**YhhI**	predicted transposase	b3484	3622401..3623537	+	Y	Y	Y	-6	TAAGGAGA(0,1,8)	N/A
**Gor**	glutathione reductase	b3500	3644322..3645674	+	Y	N	N	-8	TAAGGACA(0)	N/A
**YhiS**	hypothetical protein	b3504	3649314..3650096	+	Y	Y	Y	-4	TAAGGAAT(0,1,8)	N/A
**Slp**	outer membrane protein induced after carbon starvation	b3506	3651984..3652550	+	Y	Y	Y	-6	TAAGGATA(0,1,8)	N/A
**GadE**	acid-responsive regulator of gadA and gadBC	b3512	3656389..3656916	+	N	N	Y	-6	TAAGGAGC(8)	N/A
**MdtE**	multidrug resistance efflux transporter	b3513	3657255..3658412	+	Y	N	N	-4	CAGGGACT(0)	N/A
**GadW**	DNA-binding transcriptional activator	b3515	3661913..3662641	-	Y	Y	Y	-4	AAGGGATA(0,1,8)	N/A
**GadA**	glutamate decarboxylase A, PLP-dependent	b3517	3664203..3665603	-	Y	Y	Y	-6	TAAGGAGT(0,1,8)	N/A
**DctA**	C4-dicarboxylate transport protein	b3528	3680184..3681470	-	Y	N	N	-5	AAAGGACA(0)	N/A
**YhjR**	hypothetical protein	b3535	3694020..3694208	-	N	N	Y	-8	TAGGGATC(8)	N/A
**YiaE**	putative dehydrogenase	b3553	3715333..3716307	+	Y	Y	Y	-6	AATGGAGA(0,1,8)	N/A
**YiaF**	orf, hypothetical protein	b3554	3716357..3717067	-	Y	Y	Y	-7	AAAGGAGT(0,1,8)	N/A
**InsJ**	IS150 protein InsA	b3557	3718703..3719224	+	Y	Y	Y	-11	AATGGAAT(0,1,8)	N/A

**YiaA**	orf, hypothetical protein	b3562	3724947..3725384	-	Y	Y	Y	-7	AAAGGAAT(0,1,8)	N/A
**YiaB**	orf, hypothetical protein	b3563	3725430..3725771	-	Y	Y	Y	-6	TATGGAGA(0,1,8)	N/A
**XylB**	xylulokinase	b3564	3725940..3727394	-	N	N	Y	-8	TAAGGAAC(8)	N/A
**XylA**	xylose isomerase	b3565	3727466..3728788	-	Y	Y	Y	-6	TATGGAGT(0,1,8)	N/A
**MalS**	periplasmic alpha-amylase precursor	b3571	3735520..3737550	+	Y	N	N	-9	TAAGGACT(0)	N/A
**YiaI**	predicted hydrogenase, 4Fe-4S ferredoxin-type component	b3573	3739132..3739605	-	Y	Y	Y	-5	AAAGGAGT(0,1,8)	N/A
**YiaM**	predicted transporter	b3577	3742351..3742824	+	Y	Y	Y	-5	CAAGGAAT(0,1,8)	N/A
**YiaN**	predicted transporter	b3578	3742827..3744104	+	N	N	Y	-7	CAAGGAGC(8)	N/A
**YiaO**	predicted transporter	b3579	3744117..3745103	+	Y	Y	Y	-6	AAAGGAAA(0,1,8)	N/A
**SgbH**	3-keto-L-gulonate 6-phosphate decarboxylase	b3581	3746600..3747262	+	N	N	Y	-8	TAAGGAGC(8)	N/A
**YiaT**	hypothetical protein	b3584	3749151..3749891	-	Y	Y	Y	-8	AATGGAAT(0,1,8)	N/A
**AldB**	aldehyde dehydrogenase B (lactaldehyde dehydrogenase)	b3588	3752996..3754534	-	Y	Y	Y	-6	CAAGGAGA(0,1,8)	N/A
**YibF**	predicted glutathione S-transferase	b3592	3759370..3759978	-	Y	N	N	-5	AAAGGACT(0)	N/A
**YibT**	hypothetical protein	b4554	3774194..3774403	-	Y	N	N	-8	TACGGAGA(0)	N/A
**DinD**	DNA-damage-inducible protein	b3645	3815783..3816607	+	Y	Y	Y	-6	CATGGAGT(0,1,8)	N/A
**YicG**	orf, hypothetical protein	b3646	3816897..3817514	+	Y	Y	Y	-6	TAGGGAGA(0,1,8)	N/A
**GltS**	glutamate transporter	b3653	3825483..3826688	-	Y	Y	Y	-5	AAAGGAGT(0,1,8)	N/A
**YicI**	predicted alpha-glucosidase	b3656	3830242..3832560	-	N	N	Y	-5	TAAGGAAC(8)	N/A
**YicJ**	putative permease	b3657	3832570..3833952	-	Y	Y	Y	-6	CAAGGAAA(0,1,8)	N/A
**YicO**	orf, hypothetical protein	b3664	3840478..3841812	-	Y	N	N	-6	AATGGACA(0)	N/A
**YidH**	conserved inner membrane protein	b3676	3853983..3854330	-	N	N	Y	-5	AAAGGAGC(8)	N/A
**DgoA**	2-dehydro-3-deoxy-6-phosphogalactonate aldolase	b4477	3871018..3871635	-	N	N	Y	-8	TAAGGAGC(8)	N/A
**DgoK**	2-oxo-3-deoxygalactonate kinase	b3693	3871619..3872497	-	Y	Y	Y	-5	AAAGGAAA(0,1,8)	N/A
**DgoR**	predicted DNA-binding transcriptional regulator	b4479	3872494..3873183	-	Y	N	N	-5	CAAGGACT(0)	N/A
**YidX**	predicted lipoproteinC	b3696	3873461..3874117	+	Y	Y	Y	-5	AAAGGATT(0,1,8)	N/A
**YieE**	predicted phosphopantetheinyl transferase	b3712	3891904..3892653	+	N	N	Y	-6	CATGGAGC(8)	N/A
**YieH**	predicted hydrolase	b3715	3894797..3895462	+	Y	Y	Y	-4	AAAGGAAA(0,1,8)	N/A
**YieI**	predicted inner membrane protein	b3716	3895529..3895996	+	Y	Y	Y	-6	AAGGGAGT(0,1,8)	N/A
**YieL**	predicted xylanase	b3719	3897431..3898600	-	Y	Y	Y	-7	AAAGGAAT(0,1,8)	N/A
**BglH**	carbohydrate-specific outer membrane porin, cryptic	b3720	3898627..3900243	-	Y	Y	Y	-3	AAGGGATA(0,1,8)	N/A
**BglB**	cryptic phospho-beta-glucosidase B	b3721	3900312..3901724	-	Y	Y	Y	-6	AAAGGAGT(0,1,8)	N/A
**RbsK**	ribokinase	b3752	3935317..3936246	+	Y	N	N	-9	TATGGACA(0)	N/A
**YifN**	conserved protein (pseudogene)	b3777	3958265..3958483	-	Y	Y	Y	-10	TAAGGAGA(0,1,8)	N/A
**RffG**	dTDP-glucose 4,6-dehydratase	b3788	3970545..3971612	+	Y	Y	Y	-10	AAAGGAGT(0,1,8)	N/A
**AslB**	predicted regulator of arylsulfatase activity	b3800	3980981..3982216	+	N	N	Y	-5	CAAGGAGC(8)	N/A
**HemY**	predicted protoheme IX synthesis protein	b3802	3984709..3985905	-	Y	Y	Y	-7	CAAGGAGA(0,1,8)	N/A
**HemD**	uroporphyrinogen-III synthetase	b3804	3987111..3987851	-	Y	N	N	-10	AACGGAGA(0)	N/A
**YigF**	conserved inner membrane protein	b3817	4000442..4000822	-	Y	Y	Y	-5	TATGGAGT(0,1,8)	N/A
**PldA**	outer membrane phospholipase A	b3821	4002885..4003754	+	Y	N	N	-10	TACGGAGA(0)	N/A
**YsgA**	predicted hydrolase	b3830	4013377..4014192	-	Y	N	N	-6	TACGGAGA(0)	N/A
**TatD**	DNase, magnesium-dependent	b4483	4021577..4022359	+	Y	Y	Y	-6	TATGGAGT(0,1,8)	N/A
**RfaH**	transcriptional activator RfaH	b3842	4022356..4022844	-	Y	N	N	-10	AACGGATA(0)	N/A
**FadA**	acetyl-CoA acetyltransferase	b3845	4025632..4026795	-	Y	Y	Y	-5	TAAGGAGT(0,1,8)	N/A
**YihD**	hypothetical protein	b3858	4040092..4040361	+	N	N	Y	-7	TAAGGAGC(8)	N/A
**YihF**	putative GTP-binding protein	b3861	4042222..4043652	+	Y	Y	Y	-7	CAGGGAAT(0,1,8)	N/A
**PolA**	DNA polymerase I	b3863	4044989..4047775	+	Y	N	N	-3	CACGGACA(0)	N/A
**YihL**	predicted DNA-binding transcriptional regulator	b3872	4058470..4059180	+	Y	Y	Y	-6	AAAGGATT(0,1,8)	N/A
**OmpL**	predicted outer membrane porin L	b3875	4061626..4062318	-	Y	N	N	-5	AATGGACT(0)	N/A
**YihO**	predited transporter	b3876	4062386..4063789	-	Y	Y	Y	-5	TAAGGAGT(0,1,8)	N/A
**YihP**	predicted transporter	b3877	4063832..4065217	-	Y	Y	Y	-7	AAGGGAGA(0,1,8)	N/A
**YihQ**	alpha-glucosidase	b3878	4065263..4067299	-	Y	Y	Y	-7	TATGGAGA(0,1,8)	N/A
**YihT**	predicted aldolase	b3881	4069796..4070674	-	Y	Y	Y	-7	AAAGGAAT(0,1,8)	N/A
**YihV**	putative kinase	b3883	4071762..4072658	+	Y	Y	Y	-5	TAAGGAAT(0,1,8)	N/A
**FdhE**	formate dehydrogenase accessory protein FdhE	b3891	4078322..4079251	-	Y	N	N	-11	AACGGAAA(0)	N/A
**FdoH**	formate dehydrogenase-O, Fe-S subunit	b3893	4079880..4080782	-	Y	N	N	-7	AACGGAGA(0)	N/A
**FdoG**	formate dehydrogenase-O, large subunit	b3894	4080795..4083845	-	N	N	Y	-6	CAAGGAGC(8)	N/A
**FrvA**	predicted enzyme IIA component of PTS	b3900	4090400..4090846	-	Y	Y	Y	-8	TATGGAGT(0,1,8)	N/A
**YiiL**	L-rhamnose mutarotase	b3901	4091147..4091461	-	N	N	Y	-5	TAAGGAGC(8)	N/A
**RhaA**	L-rhamnose isomerase	b3903	4092746..4094005	-	N	N	Y	-8	AAAGGAGC(8)	N/A
**Cdh**	CDP-diacylglycerol pyrophosphatase	b3918	4107953..4108708	+	Y	Y	Y	-6	CAGGGAAA(0,1,8)	N/A
**YiiR**	conserved inner membrane protein	b3921	4110338..4110778	+	N	N	Y	-5	CAAGGAAC(8)	N/A
**MetB**	cystathionine gamma-synthase	b3939	4126695..4127855	+	N	N	Y	-9	CAGGGAAC(8)	N/A
**GldA**	glycerol dehydrogenase, (NAD)	b3945	4135955..4137058	-	N	N	Y	-5	AAAGGAGC(8)	N/A
**FsaB**	fructose-6-phosphate aldolase 2	b3946	4137069..4137731	-	Y	Y	Y	-5	AAAGGAAA(0,1,8)	N/A
**YijO**	predicted DNA-binding transcriptional regulator	b3954	4145489..4146340	-	Y	Y	Y	-4	AAGGGAAT(0,1,8)	N/A
**FabR**	DNA-binding transcriptional repressor	b3963	4159147..4159794	+	Y	Y	Y	-12	CAAGGATT(0,1,8)	N/A
**HtrC**	heat shock protein	b3989	4187809..4188348	+	N	N	Y	-5	TAAGGAAC(8)	N/A
**HemE**	uroporphyrinogen decarboxylase	b3997	4195739..4196803	+	N	N	Y	-8	TAAGGAAC(8)	N/A
**Nfi**	endonuclease V (deoxyinosine 3'endoduclease)	b3998	4196813..4197484	+	Y	Y	Y	-5	TAAGGAGT(0,1,8)	N/A
**YjaA**	hypothetical protein	b4011	4211257..4211640	+	Y	Y	Y	-4	AAGGGAAT(0,1,8)	N/A
**YjaB**	predicted acetyltransferase	b4012	4211703..4212146	-	Y	Y	Y	-5	AAAGGAGA(0,1,8)	N/A
**AceA**	isocitrate lyase	b4015	4215132..4216436	+	N	N	Y	-9	TATGGAGC(8)	N/A
**YjbB**	predicted transporter	b4020	4225754..4227385	+	Y	Y	Y	-11	TAAGGAGA(0,1,8)	N/A
**YjbE**	hypothetical protein	b4026	4233929..4234171	+	Y	Y	Y	-6	AAGGGAAA(0,1,8)	N/A
**MalG**	maltose transporter subunit	b4032	4240649..4241539	-	Y	Y	Y	-9	AAGGGAGA(0,1,8)	N/A
**MalK**	fused maltose transport subunit, ATP-binding component of ABC superfamily/regulatory protein	b4035	4244807..4245922	+	Y	Y	Y	-6	AAGGGAGA(0,1,8)	N/A
**UbiC**	chorismate lyase	b4039	4250529..4251026	+	Y	N	N	-8	AACGGAGA(0)	N/A
**Alr**	alanine racemase	b4053	4263805..4264884	+	N	N	Y	-5	CAAGGAAC(8)	N/A
**AphA**	acid phosphatase/phosphotransferase, class B, non-specific	b4055	4267437..4268150	+	Y	Y	Y	-4	TAGGGAAA(0,1,8)	N/A
**YjbQ**	hypothetical protein	b4056	4268261..4268677	+	N	N	Y	-6	CAAGGAGC(8)	N/A
**YjcE**	predicted cation/proton antiporter	b4065	4278003..4279652	+	N	N	Y	-3	CAGGGAAC(8)	N/A
**YjcF**	hypothetical protein	b4066	4279806..4281098	-	Y	Y	Y	-5	TAAGGATA(0,1,8)	N/A
**Acs**	acetyl-coenzyme A synthetase	b4069	4283436..4285394	-	Y	Y	Y	-9	CAAGGAGA(0,1,8)	N/A
**NrfB**	formate-dependent nitrite reductase; a penta-haeme cytochrome c	b4071	4287268..4287834	+	Y	Y	Y	-5	AATGGAGT(0,1,8)	N/A
**NrfC**	formate-dependent nitrite reductase, 4Fe4S subunit	b4072	4287831..4288502	+	N	N	Y	-5	TAAGGAGC(8)	N/A
**YjcP**	predicted outer membrane factor of efflux pump	b4080	4297587..4299053	-	N	N	Y	-7	CAAGGAGC(8)	N/A
**YjcS**	orf, hypothetical protein	b4083	4302635..4304620	-	Y	Y	Y	-6	AATGGAGA(0,1,8)	N/A
**AlsE**	allulose-6-phosphate 3-epimerase	b4085	4305806..4306501	-	Y	Y	Y	-6	TAAGGAAT(0,1,8)	N/A
**RpiR**	transcriptional repressor of rpiB expression	b4089	4310124..4311014	-	Y	N	N	-11	CAAGGACA(0)	N/A
**PhnG**	carbon-phosphorus lyase complex subunit	b4101	4319267..4319719	-	N	N	Y	-6	TATGGAGC(8)	N/A
**YjdL**	predicted transporter	b4130	4352977..4354434	-	Y	Y	Y	-5	TAAGGAGA(0,1,8)	N/A
**YjeJ**	hypothetical protein	b4145	4371388..4372257	-	Y	Y	Y	-7	TAGGGAGA(0,1,8)	N/A
**Blc**	outer membrane lipoprotein (lipocalin)	b4149	4375212..4375745	-	Y	Y	Y	-6	TAAGGAAA(0,1,8)	N/A
**AmpC**	beta-lactamase/D-alanine carboxypeptidase	b4150	4375834..4376967	-	Y	Y	Y	-10	TATGGAAA(0,1,8)	N/A
**FrdD**	fumarate reductase subunit D	b4151	4377030..4377389	-	N	N	Y	-6	TAAGGAGC(8)	N/A
**FrdC**	fumarate reductase subunit C	b4152	4377400..4377795	-	Y	Y	Y	-6	TAAGGAGT(0,1,8)	N/A
**YjeP**	predicted mechanosensitive channel	b4159	4384070..4387393	-	Y	Y	Y	-8	AAAGGAAA(0,1,8)	N/A
**Hfq**	RNA-binding protein Hfq	b4172	4398311..4398619	+	Y	Y	Y	-8	TAAGGAAA(0,1,8)	N/A
**YjeT**	conserved inner membrane protein	b4176	4402409..4402606	+	Y	Y	Y	-5	TAAGGATA(0,1,8)	N/A
**YjfI**	hypothetical protein	b4181	4408156..4408557	+	Y	Y	Y	-6	TATGGAAT(0,1,8)	N/A
**YjfK**	hypothetical protein	b4183	4409325..4409984	+	Y	Y	Y	-5	TAAGGATA(0,1,8)	N/A
**YjfN**	orf, hypothetical protein	b4188	4414040..4414315	-	N	N	Y	-6	AATGGAGC(8)	N/A
**YjfP**	predicted hydrolase	b4190	4414975..4415724	+	Y	Y	Y	-6	AAAGGAGA(0,1,8)	N/A
**UlaB**	L-ascorbate-specific enzyme IIB component of PTS	b4194	4419416..4419721	+	Y	Y	Y	-8	TAAGGAGT(0,1,8)	N/A
**UlaE**	L-xylulose 5-phosphate 3-epimerase	b4197	4420869..4421723	+	N	N	Y	-5	TAAGGAGC(8)	N/A
**FklB**	FKBP-type 22KD peptidyl-prolyl cis-trans isomerase (rotamase)	b4207	4426958..4427578	+	Y	Y	Y	-5	AAAGGAAA(0,1,8)	N/A
**YtfM**	predicted outer membrane protein and surface antigen	b4220	4440405..4442138	+	Y	Y	Y	-14	AAAGGATA(0,1,8)	N/A
**Ppa**	inorganic pyrophosphatase	b4226	4447145..4447675	-	Y	Y	Y	-6	AAAGGAAA(0,1,8)	N/A
**YtfT**	predicted sugar transporter subunit: membrane component of ABC superfamily	b4230	4450594..4451619	+	Y	Y	Y	-6	TAAGGAGA(0,1,8)	N/A
**YjfF**	putative transport system permease protein	b4231	4451606..4452601	+	Y	Y	Y	-9	AAAGGAGT(0,1,8)	N/A
**Fbp**	fructose-1,6-bisphosphatase	b4232	4452634..4453632	-	Y	Y	Y	-6	CAGGGAAA(0,1,8)	N/A
**Mpl**	UDP-N-acetylmuramate:L-alanyl-gamma-D-glutamyl- meso-diaminopimelate ligase	b4233	4453808..4455181	+	Y	Y	Y	-8	AAAGGATA(0,1,8)	N/A
**YjgA**	hypothetical protein	b4234	4455337..4455888	-	N	N	Y	-6	TAAGGAGC(8)	N/A

**NrdD**	anaerobic ribonucleoside triphosphate reductase	b4238	4458545..4460683	-	N	N	Y	-5	TATGGAGC(8)	N/A
**YjgI**	predicted oxidoreductase with NAD(P)-binding Rossmann-fold domain	b4249	4471363..4472076	-	Y	Y	Y	-5	CAAGGAGT(0,1,8)	N/A
**YjgK**	orf, hypothetical protein	b4252	4472885..4473337	+	Y	Y	Y	-8	TAAGGAGT(0,1,8)	N/A
**YjgL**	orf, hypothetical protein	b4253	4473460..4475274	+	Y	Y	Y	-6	CAAGGAGT(0,1,8)	N/A
**YjgN**	conserved inner membrane protein	b4257	4477753..4478949	+	Y	Y	Y	-5	CAGGGAAT(0,1,8)	N/A
**YjgQ**	orf, hypothetical protein	b4262	4485341..4486423	+	N	N	Y	-7	AAAGGAGC(8)	N/A
**IdnO**	gluconate 5-dehydrogenase	b4266	4490610..4491374	-	Y	Y	Y	-7	AAAGGAAT(0,1,8)	N/A
**YjgZ**	KpLE2 phage-like element; predicted protein	b4277	4499283..4499612	+	Y	Y	Y	-13	CAGGGAGA(0,1,8)	N/A
**YjhH**	putative lyase/synthase	b4298	4522128..4523033	-	Y	Y	Y	-9	AAAGGAAA(0,1,8)	N/A
**SgcA**	KpLE2 phage-like element; predicted phosphotransferase enzyme IIA component	b4302	4525572..4526003	-	Y	Y	Y	-10	CATGGAGT(0,1,8)	N/A
**SgcQ**	KpLE2 phage-like element; predicted nucleoside triphosphatase	b4303	4526134..4526940	-	Y	Y	Y	-8	TAAGGAGT(0,1,8)	N/A
**SgcX**	putative lyase/synthase	b4305	4528553..4529674	-	Y	N	N	-6	TACGGAGA(0)	N/A
**YjhQ**	KpLE2 phage-like element; predicted acetyltransferase	b4307	4531262..4531807	-	Y	Y	Y	-7	TAAGGAGA(0,1,8)	N/A
**FimB**	tyrosine recombinase/inversion of on/off regulator of fimA	b4312	4538980..4539582	+	Y	Y	Y	-5	AAGGGAAA(0,1,8)	N/A
**FimA**	major type 1 subunit fimbrin (pilin)	b4314	4541138..4541686	+	Y	Y	Y	-6	AAAGGAAA(0,1,8)	N/A
**UxuR**	DNA-binding transcriptional repressor	b4324	4552599..4553372	+	Y	N	N	-8	AACGGACA(0)	N/A
**IadA**	isoaspartyl dipeptidase	b4328	4556377..4557549	-	Y	Y	Y	-5	CAAGGAGT(0,1,8)	N/A
**YjiG**	conserved inner membrane protein	b4329	4557562..4558023	-	N	N	Y	-7	CAAGGAGC(8)	N/A
**YjiK**	conserved protein	b4333	4560766..4561626	-	Y	Y	Y	-4	AAGGGATT(0,1,8)	N/A
**YjiL**	putative enzyme	b4334	4561945..4562712	-	Y	Y	Y	-5	TAAGGAGT(0,1,8)	N/A
**YjiO**	multidrug efflux system protein	b4337	4565310..4566542	-	Y	Y	Y	-9	CAAGGAGT(0,1,8)	N/A
**YjiR**	fused predicted DNA-binding transcriptional regulator/predicted aminotransferase	b4340	4568185..4569597	-	Y	Y	Y	-6	TAAGGAAT(0,1,8)	N/A
**YjiT**	orf, hypothetical protein	b4342	4570437..4571954	+	N	N	Y	-6	TAAGGAGC(8)	N/A
**HsdR**	endonuclease R	b4350	4581272..4584784	-	Y	Y	Y	-8	AATGGATT(0,1,8)	N/A
**YjiA**	orf, hypothetical protein	b4352	4585932..4586888	-	Y	Y	Y	-5	AATGGAGA(0,1,8)	N/A
**YtjA**	predicted protein	b4568	4610151..4610312	+	Y	Y	Y	-6	AAAGGAGA(0,1,8)	N/A
**YjjI**	hypothetical protein	b4380	4613538..4615088	-	Y	Y	Y	-6	AAAGGAAA(0,1,8)	N/A
**DeoB**	phosphopentomutase	b4383	4617626..4618849	+	Y	N	N	-5	TACGGAGA(0)	N/A
**LplA**	lipoate-protein ligase A	b4386	4621124..4622140	-	Y	Y	Y	-6	AAAGGAAA(0,1,8)	N/A
**YtjC**	phosphoglycerate mutase	b4395	4631820..4632467	+	Y	N	N	-7	TACGGAAA(0)	N/A
**CreD**	inner membrane protein	b4400	4636201..4637553	+	Y	Y	Y	-6	AAAGGAGA(0,1,8)	N/A
